# Renewable energy, information and communication technology, financial development, natural resources, institutional quality, and agricultural productivity in Asian countries

**DOI:** 10.1371/journal.pone.0355560

**Published:** 2026-08-14

**Authors:** Tinh Tran Phu Do, Hue Trinh Hoang Hong, Duyen Thi My Thi

**Affiliations:** 1 University of Economics and Law, Ho Chi Minh City, Vietnam; 2 Vietnam National University, Ho Chi Minh City, Vietnam; Ural Federal University named after the first President of Russia B N Yeltsin Institute of Physics and Technology: Ural'skij federal'nyj universitet imeni pervogo Prezidenta Rossii B N El'cina Fiziko-tehnologiceskij institut, RUSSIAN FEDERATION

## Abstract

This study highlights the nexus between renewable energy consumption (REC), information and communication technologies (ICT), financial development (FD), natural resources (NR), institutional quality (IQ), and agricultural productivity (AGP) in 29 Asian countries for the 2000–2021 period. The fully modified least-squares (FMOLS) and dynamic least-squares (DOLS) estimation are used for the empirical analysis. The Driscoll and Kraay estimation (DSK) and the generalised method of moments (GMM) are used for robustness checks. The empirical results indicate that REC, ICT, FD, IQ have a significant positive impact on AGP. Specifically, a unit increase in REC, ICT, FD and IQ lead to a 6.097%, 2.292%, 0.688% and 0.002% points increase in AGP, respectively. Meanwhile, NR has a significant negative influence on AGP. Specifically, a unit increase in NR leads to 0.226% points reduce in AGP, implying that the resource curse persists in Asian countries. The results of the causality test illustrated bidirectional causality between FD, IQ, and AGP. Compared with previous studies in the Asian region, this study provides additional empirical evidence on the impact of REC and ICT on AGP, and the interaction of these two factors on agricultural development in the region. The research results suggest that managers need to issue policies to enhance sustainable AGP through the development of renewable energy sources, the application of ICT in agricultural production, and capital incentives for green projects to promote sustainable agricultural growth for Asian countries.

## 1. Introduction

In Asia, agriculture plays a vital role in the livelihoods of its people. Over 60% of the population, approximately 2.2 billion individuals, rely on agriculture for their income. However, the agricultural sector’s contribution to the region’s gross domestic product (GDP) has been declining, falling from an average of 11.1% in 2000 to 7.1% in 2023 [1]. Climate change is threatening agricultural production, and thus, the livelihoods and food security of billions of people who depend on agriculture in the Asian region. Agriculture is highly vulnerable to climate change because of its dependence on the climate and weather. According to the Food and Agriculture Organization (FAO), Asia’s total emissions from agricultural activities in 2018 reached 3.3 GtCO2e, which was nearly one-third of the global share from agriculture. China and India were significant emitters from agricultural production in 2018, with each country contributing around 650 million tonnes (Mt) of CO2eq annually [2]. To make agriculture more sustainable, it is essential to ensure food security, enhance the value of agricultural products, and decrease carbon emissions from the agricultural sector.

Countries are committed to achieving sustainable development goals, by halving emissions by 2030, and moving towards zero emissions by 2050. Therefore, innovation in agricultural production activities is urgent to realize the goal of reducing emissions, increasing agricultural productivity, ensuring food security and income for current and future generations, towards the goal of sustainable development. According to [3], renewable energy helps increase agricultural productivity (AGP) by providing a clean, stable source of energy for activities such as irrigation, drying agricultural products, lighting, and operating modern equipment, thereby reducing production costs, improving efficiency and promoting sustainable agricultural development. Therefore, considering the impact of renewable energy consumption (REC) on agricultural development in the Asian region is urgent in the current context. According to [4], information and communication technologies (ICT) increases AGP because it helps optimize production processes and increase resource efficiency. Renewable energy provides clean, economical electricity for agricultural activities, while ICT provides monitoring and automatic control solutions, optimizes resource use and enhances adaptability to climate change, contributing to building a modern, efficient and sustainable agricultural system. Integrating ICT into agricultural production might allow farmers to better adapt to climate change through more modern production models. Moreover, the application of technology helps optimize resource use, increase productivity and reduce greenhouse gas emissions in agricultural production, while financial and institutional development plays an important role in investing, coordinating and creating a favorable legal corridor for the application of these technologies, thereby improving agricultural productivity in a sustainable manner [5]. This study examines between renewable energy consumption (REC), information and communication technologies (ICT), financial development (FD), natural resources (NR), institutional quality (IQ), and agricultural productivity (AGP). Based on the research findings, the authors will propose several policy implications to promote agricultural growth towards the goal of sustainable agricultural development in this region.

This study makes several contributions to the existing literature. Although previous studies have assessed the relationship between AGP and its determinants for various regions. There is still a lack of research on the case of Asian countries. Specifically, studies have not yet emphasized the decisive role of REC and ICT on AGP in Asia. Thus, this study will explore how ICT facilitates the use of mobile technology to share knowledge and access weather information, aiding farmers in making informed decisions to enhance productivity and promote sustainable agriculture [6]. Furthermore, renewable energy plays an important role in enhancing sustainable AGP. Because it helps reduce emissions from agricultural production activities, allowing agricultural products to be more environmentally friendly, towards sustainable agriculture [3]. The interaction between REC and ICT can have a positive impact on AGP as they can help reduce environmental impacts, increase production efficiency and improve climate change adaptation. However, there is currently a lack of research examining this interaction on agricultural production in Asian regions. Through this study, we will provide more empirical evidence on this interaction. From there, the authors will suggest some policy implications to enhance AGP in the region. Furthermore, financial resources are considered extremely important for farmers to invest in agricultural production activities, enabling them to invest in technology and equipment for smart farming. The combination of financial development, information and communication technology (ICT), and renewable energy provides a strong impetus for sustainable agriculture. Finance drives investment in high technology, ICT optimizes production processes promoting smart farming models, and the consumption of renewable energy such as solar and biomass reduces operating costs, thus facilitating a green transformation of the agricultural sector, increasing value and productivity. This study will provide further empirical evidence on this relationship, thereby suggesting some key policy implications toward the Sustainable Development Goals (SDGs) for countries in the Asian region, specifically Goal 2.4.

The authors use recent econometric methods to analyze the model. First, we tested the cross-sectional dependence (CSD). Because if the research skips this step, the results may be distorted [7]. The CSD exists in the panel data, the athors applied the second-generation root tests for the analysis, and the panel cointegration tests are also applied. The fully modified least-squares (FMOLS) and dynamic least-squares (DOLS) methods were used for empirical analysis. The Driscoll-Kraay standard error (DK)’ approach and the generalised method of moments (GMM)-system estimator were used to check robustness.

The structure of this study is as follows: Section 2 provides a brief overview of the literature; Section 3 presents the methodology and data; Section 4 illustrates the outcomes and discussions; and Section 5 concludes the paper.

## 2. Literature review

### 2.1. Renewable energy and the agricultural productivity

The world is facing a major challenge of climate change, partly caused by carbon emissions. To counter this risk, renewable energy should be considered for its contribution to Sustainable Development Goals (SDGs) for global countries. Several studies have examined the role of REC in enhancing sustainable agricultural productivity. For instance, [3] examined the link between REC and AGP in 123 nations during the period 1995–2019. They show that REC, foreign direct investment (FDI), and financial inclusion has a positive influence on AGP, whereas carbon dioxide emission (CO_2_E) show the opposite. Research suggests that farmers should reduce the consumption of non-renewable energy sources for agricultural production and switch to renewable energy. Additionally, [8] investigated the link between AGP and REC in BRICS from 2000 to 2020. The outcomes indicate that REC, CO2E, and FDI positively affect AGP, whereas access to financial inclusion has no impact on AGP. [9] assessed the influence of climate change and FD on agricultural production in ASEAN-4 from 1990–2016. They found that climate change hurt gricultural production, whereas renewable energy, human capital (HC), and institutional quality (IQ) positively affected AGP. [10] evaluated the impact of REC and NR on AGP in ASEAN countries. They found that REC positively affected the AGP, whereas the forest area, CO2E, and NR negatively affected AGP. Similarly, [11] found that REC increases AGP in Middle East and North African nations. This study suggests that governments should target the development of renewable energy sources, particularly in the agricultural sector. REC boosts agricultural productivity was also found in the study of [4,12–15].

Generally, some studies examined the nexus between REC and sustainable agricultural development, the outcomes of this influence in Asia still need to be clarified. This study will provide further empirical evidence on the impact of REC on AGP in Asian countries, thereby suggesting policy implications to promote sustainable agriculture in this region.

### 2.2. Information and communication technologies (ICT) and the agricultural productivity

The effective use of ICT to improve access to appropriate agricultural information and services holds great promise for transforming farmers’ productivity and livelihoods. [16] examined the effect of ICT on agriculture sector performances (ASP) in sub-Saharan Africa (SSA) for the period of 1995–2017 by employing panel autoregressive distributed lag (ARDL) approach. The results show that ICT has a positive impact on ASP in the long run. However, there is no evidence that this effect persists in the short term. Similarly, [4] examined the effect of RE, ICT and agricultural credit (AC) for the sustainable AGP in developing nations for the 2000–2021 period by employing the Driscoll-Kraay and Quantile Regression to check robustness. They indicate that ICT have a positive impact on AGP. The moderating effect of the interaction between ICT and renewable energy is negative and weak. [6] investigated the mobile phones to support agricultural business management in Indonesia. The results show that mobile phone usage allows farmers to enhance sales, get market information, and acces to advanced agricultural technology. This helps increase profits for farmers. The study suggests encouraging farmers to use mobile phones to access information for agricultural business activities. According to [17], mobile networks are increasingly popular, making them easily accessible to users, which makes it easier for farmers to collect and share agricultural knowledge with each other. Similarly, according to [18], mobile phones enable farmers to easily exchange market information, receive weather updates, obtain agricultural extension advice, and learn about new farming technologies, thereby enhancing agricultural productivity. Similar results were also found in the study of [19,20].

In short, several studies have investigated the impact of ICT on AGP, but this nexus still needs to be clarified in the Asian region. In addition, this study also examines the interaction effect of REC and ICT on AGP. Thereby, the authors will suggest some policy implications for the goal of sustainable AGP in the region for the coming time.

### 2.3. Financial development (FD) and the agricultural productivity

Investing financial resources for agricultural development is necessary, it allows farmers to convert to a more modern agricultural production model by applying modern technology, which helps the agricultural sector increase productivity and product quality. The important role of financial development in economic development has been proven by many studies, its role in the agricultural sector also needs to be clarified. [21] indicated that commercial loans to the farming sector significantly affect production in the country. Accordingly, the study suggests that commercial banks should support low-interest loans to farmers. Additionally, [22] examined the effect of financial development on AGP in South Asia and found the inverted U-shaped result of FD on agricultural production. Similar results were also found in the study of [9] for 4 ASEAN nations. They suggest that governments must adopt flexible financial and agricultural policies so that farmers can benefit and AGP can be increased. On the contrary, according to [23] digital inclusive finance reduces households’ agricultural output. However, Chandio, Jiang [24] examined the influence of global climate change, financial development (FD), and technical progress on cereal production in Pakistan from 1977 to 2014. They found that CO2 emissions negatively affect cereal production, whereas FD and technical progress have a positive influence on cereal production. Research suggests that financial development is necessary for sustainable AGP. Similar results were also found in the studies of [25–27], they showed that financial development enhances AGP.

In general, the important role of financial development in economic development has been proven by many studies, its role in the agricultural sector also needs to be clarified. Some studies show that financial development boosts AGP, however some studies claim that financial development reduces AGP [23]. The authors will provide further empirical evidence on this impact, in the case of Asian countries. Thereby, propose some policy implications to promote sustainable AGP through financial instruments.

### 2.4. Natural resources (NR) and the agricultural productivity

The demand for fossil energy is increasing to serve economic growth, which leads to the overuse of natural resources and also causes large emissions. Recently, NR and environmental pollution have attracted much attention from researchers. Much of the literature focuses on the impact of NR on economic growth, emissions, and poverty. Few studies have examined the impact of NR on the agricultural sector. [10] show that CO_2_E, forest area, and NR decrease AGP. Similarly, [28] explored that resource windfalls reduce AGP in Sub-Saharan Africa, confirming the resource curse of agriculture in these countries. In the case of 25 oil-exporting nations, [29] studied the impact of oil prices on agriculture and showed that oil prices harmed on AGP. Implying that Dutch disease exists in these countries. In another research, [30] examined the impact of oil rents on AGP in the Middle East and North Africa (MENA) nations. They discovered a negative link between oil rents and AGP, showing that the oil industry boom is associated with a contraction in agriculture. Likewise, [31] discovered a negative nexus between NR abundance and AGP in Asia-Pacific Economic Cooperation (APEC) countries, implying that natural resource decreases AGP. This confirms the existence of a resource curse in these countries, due to an over-concentration in the resource sector, leading to the neglect of other sectors, increasing dependence on commodity price fluctuations. Poor governance, corruption and political instability also exacerbate this situation, preventing resources from being used effectively to promote sustainable growth.

In summary, several studies have examined the role of NR in economic growth, but few studies have examined the impact of NR on agriculture sector, particularly in Asia. Some studies have found that NR has a negative impact on AGP [10,31]. The authors will provide further empirical evidence on this relationship in Asian countries, thereby suggesting some policy implications for promoting AGP.

### 2.5. Institutional quality (IQ) and the agricultural productivity

IQ plays a fundamental role in determining the sustainable and efficient development of agriculture by creating a transparent business environment, protecting property rights, and promoting investment. Good institutions help reduce transaction costs, encourage the adoption of high technology, and optimize resource management. [32] emphasized that IQ plays a crucial role in determining the growth of agricultural value added in East Africa. The research suggests that strengthening and improving the operational efficiency of institutions is necessary to promote sustainable agricultural growth. However, [33] demonstrates the negligible role of institutional quality in achieving food security in Africa. The authors recommend that Africa should reposition its focus on institutional quality, emphasizing sustainable food security as part of regional poverty reduction efforts. [34] emphasized that in Zimbabwe, the political and economic institutions supporting tobacco production are weak and below optimal levels for promoting production. The research suggests that Zimbabwe needs to strengthen institutions supporting production, allocate land to farmers, and encourage the adoption of sustainable green agriculture. [35] emphasized that the interaction between IQ and agricultural credit has a significant impact on agricultural output. The study recommends prioritizing efforts to improve IQ to create a favorable environment for agricultural growth.

### 2.6. Research gap

Although previous studies have assessed the relationship between AGP and its determinants for various regions. There is still a lack of research on the case of Asian countries. Specifically, studies have not yet emphasized the decisive role of REC and ICT on AGP in Asia. Therefore, this study will explore how ICT supports farmers in making informed decisions to improve productivity and promote sustainable agriculture [6]. Furthermore, renewable energy plays an important role in enhancing sustainable AGP. Because it helps reduce emissions from agricultural production activities, allowing agricultural products to be more environmentally friendly, towards sustainable agriculture [3]. The interaction between REC and ICT can have a positive impact on AGP as they can help reduce environmental impacts, increase production efficiency and improve climate change adaptation. However, there is currently a lack of research examining this interaction on agricultural production in Asian regions. Through this study, we will provide more empirical evidence on this interaction. From there, the authors will suggest some policy implications to enhance AGP in the region. Furthermore, financial resources are considered extremely important for farmers to invest in agricultural production activities, enabling them to invest in technology and equipment for smart farming. The combination of financial development, information and communication technology (ICT), renewable energy, natural resources and institutional quality provide a strong impetus for sustainable agriculture. Finance drives investment in high technology, ICT optimizes production processes promoting smart farming models, and the consumption of renewable energy such as solar and biomass reduces operating costs, thus facilitating a green transformation of the agricultural sector, increasing value and productivity. Strong institutions can promote the sustainable use of resources, reduce depletion, thereby increasing productivity and ensuring food security. Good resource planning and management, along with the right policies, are key to maintaining sustainable productivity. This study will provide further empirical evidence on this relationship, thereby suggesting some key policy implications toward the Sustainable Development Goals (SDGs) for countries in the Asian region, specifically Goal 2.4.

In terms of applied methods, there are also differences from previous studies, for example, [4] applied the ‘Driscoll-Kraay for empirical analysis and the Method of Moments Quantile Regression is used to check robustness. In our study, modern econometric methods were applied, namely, the fully modified least-squares (FMOLS) and dynamic least-squares (DOLS) methods. Because they are capable of solving the endogeneity problem and serial correlation errors in time series models, they help to estimate the long-run relationship reliably. In addition, the authors used The Driscoll-Kraay (DSK) estimator and the generalized method of moments (GMM) to check the robustness. The DSK estimate is appropriate for the empirical analysis in the case of large cross-sections (29) and small time periods (22), as in this study, and solves the problems of heteroscedasticity, autocorrelation, and the cross-sectional dependence (CSD) in empirical models [36]. Besides, in order to address the endogeneity problem in the model, the authors used GMM estimation to check the robustness. Furthermore, our study also test the causal relationship between variables using Pairwise Dumitrescu Hurlin Panel Causality Tests.

## 3. Methodology and data

### 3.1. Data and variables

This study utilizes a panel dataset comprising 29 Asian countries, with data spanning the period from 2000 to 2021. Data is collected from the World Bank and the International Monetary Fund (IMF). In this research, AGP is the dependent variable, and REC, ICT, NR, FD and IQ are explanatory variables. Table 1 provides details of all the variables and their sources.

**Table 1:** Describe variables.

Variables	Measurement	Data Source	References
Agricultural productivity (AGP)	The natural logarithm of agriculture, forestry, and fishing, value added (current LCU)	WDI	[4]
Renewable energy consumption (REC)	The natural logarithm of renewable energy consumption (% of total final energy consumption)	WDI	[3]
Information and communication technologies (ICT)	The natural logarithm of mobile cellular subscriptions	WDI	[4]
Natural resources (NR)	The natural logarithm of total natural resources rents (% of GDP)	WDI	[37]
Financial development (FD)	The natural logarithm of financial development index	IMF	[9]
Institutional quality (IQ)	Voice and Accountability	WDI	[32]

In the context of increasing climate change, which threatens food security, it is necessary to examine the nexus among REC, ICT, FD, NR, IQ and AGP in the Asian region. The research results will provide empirical evidence, thereby suggesting policy suggestions to promote AGP in the region. AGP can be driven by renewable energy through reduced dependence on fossil fuels, ICT through its ability to optimize processes and improve efficiency. Moreover, the application of technology helps optimize resource use, increase productivity and reduce greenhouse gas emissions in agricultural production, while financial and institutional development plays an important role in investing, coordinating and creating a favorable legal corridor for the application of these technologies, thereby improving agricultural productivity in a sustainable manner [5]. According to [4], REC and ICT promote sustainable AGP. Because using energy from clean sources such as solar, wind, biomass helps reduce greenhouse gas emissions, limit environmental pollution and protect natural resources. Besides, ICT provides solutions such as smart irrigation systems, data-based farm management, helping farmers make more accurate decisions about planting, caring, and harvesting. In addition, with wide coverage, ease of use, multi-function and low cost, mobile devices, especially phones, can solve the biggest challenge of farmers in connecting to the market, promoting the commercialization of agricultural products, helping the agricultural industry develop more strongly and sustainably. However, the interactive impact between REC and ICT on agricultural production is negative [4]. Therefore, our study also provides further empirical evidence on this interaction effect on AGP in the Asian region. Furthermore, financial development plays a vital role in enhancing AGP as it provides the necessary capital to invest in environmentally friendly farming methods, modern technology and capacity building for farmers, thereby ensuring long-term economic benefits, improving farmers’ lives and protecting natural resources. [24] stated that FD positively affect AGP, they suggested that the Pakistan government should invest financial resources in sustainable agricultural development. However, [23] indicated that digital inclusive finance reduces households’ agricultural output. Thus, it is necessary to consider the impact of financial development on AGP in Asian countries. From the research results, the authors will suggest policy implications to promote AGP in this region. Moreover, institutional quality, particularly voice and accountability mechanisms, plays an important role in promoting AGP by allowing stakeholders to have a voice in policymaking, holding institutions accountable for their actions, and ensuring that agriculture initiatives are designed and implemented effectively, reflect real needs, and aim for sustainable outcomes. However, [37] agrued that institutional quality negatively affect AGP, they suggest that governments need to enact stricter regulations towards sustainable AGP. Farmers’ voices help policymakers better understand the challenges and opportunities in agriculture production, thereby building more appropriate and effective policies for sustainable development. Therefore, this study will examine the impact of IQ, specifically voice and accountability, on AGP, thereby suggesting solutions towards sustainable agricultural growth in the Asian region.

### 3.2. Theoretical framework and study hypothesis

The factors influencing agricultural production are presented based on the production function, where productivity is expressed in terms of functional inputs. Theoretically, agricultural productivity depends on capital (K) and labor (L). However, according to [38], the production function can be expanded using more input variables. The nexus between inputs and outputs in agricultural production can be presented based on the Cobb–Douglas production function was developed by [38] as follows:


$$\begin{array}{c} {\text{Y}}_{\text{it}}={\theta \text{K}}_{{\text{it}}^{\alpha }}\;{\text{L}}_{{\text{it}}^{\beta }}{\text{E}}_{{\text{it}}^{\varepsilon }} \end{array}$$ (1)

Where Y is output, is measured by AGP, K is capital, and L is labor, and E is other input. This study examined the effect of REC, ICT, NR, FD and IQ on AGP. When we calculate the natural logarithm of Equation (1) and incorporate REC, ICT, NR, FD and IQ, following the studies [36,37] as mentioned in Equation (2).


$$\begin{array}{c} {lnAGP}_{i,t}=\alpha_{\text{i},\text{t}}+{lnREC}_{i,t}+{lnICT}_{i,t}+{lnNR}_{i,t}{+{lnFD}_{i,t}+{IQ}_{i,t}+\varepsilon }_{\text{i},\text{t}} \end{array}$$ (2)

Furthermore, Equation (2) is widened by introducing the interaction of (LnREC*LnICT) to examine the moderating influence of REC with ICT on AGP, following the studies [4,36] as mentioned in Equation (3).


$$\begin{array}{c} {lnAGP}_{i,t}=\alpha_{\text{i},\text{t}}+{lnREC}_{i,t}+{lnICT}_{i,t}+\text{lnREC}*\text{lnICT}+{lnNR}_{i,t}{+{lnFD}_{i,t}+{IQ}_{i,t}+\varepsilon }_{\text{i},\text{t}} \end{array}$$ (3)

Asian countries face great challenges from climate change, which affects agricultural production and threatens national food security. Improving AGP while reducing emissions is the path towards sustainable agricultural development and green growth. The agricultural sector in developing countries plays an important role in creating jobs and ensuring national food security. On the other hand, this sector is also responsible for climate change, Asia’s total emissions from agricultural activities in 2018 reached 3.3 GtCO2e, which was nearly one-third of the global share from agriculture [2]. Therefore, to achieve the goal of sustainable agricultural development, it is necessary to use environmentally friendly inputs. According to [3] stated that REC increases agricultural productivity and helps reduce CO_2_E emissions [39].

The increased consumption of renewable energy has a positive impact on agricultural productivity through reduced production costs, improved resource efficiency, and the promotion of sustainable development. Solar, wind, and biomass power systems provide a stable energy supply for irrigation, processing, and storage of agricultural products, thereby improving quality and yield. In addition, renewable energy contributes to reducing greenhouse gas emissions, improving soil and water environments, and creating favorable conditions for long-term agricultural production. [40] emphasized that renewable energy is a crucial factor in ensuring food and energy security, while also enhancing agricultural productivity through the integration of green technologies into production. Thus, in this research, we assess the role of REC in enhancing AGP in the Asia region.

**Hypothesis 1**. REC significantly increases AGP in Asian countries

ICT is increasingly becoming a key factor in improving agricultural productivity. Through the provision of timely market information, accurate weather forecasts, and digital extension services, ICT helps farmers make more effective production decisions, minimize risks, and increase income. Furthermore, early warning systems and digital financial services support farmers in disease prevention, access to capital and crop insurance, contributing to sustainable production. [41] showed that the adoption of ICT significantly improved the technical efficiency of small-scale vegetable farmers in China. Similarly, [42] emphasized that ICT in extension services helps bridge the information gap and enhance agricultural productivity. According to [4], ICT helps improve agricultural productivity and research suggests that developing countries need to integrate ICT in agricultural production. Therefore, in this study, we examine the effect of ICT on AGP in the Asia.

**Hypothesis 2.** ICT significantly increases AGP in the Asia.

ICT optimizes processes, helping farmers make better data-driven decisions, while renewable energy provides clean power for agricultural activities such as irrigation, lighting, heating and waste treatment, thereby creating an efficient, resilient and environmentally friendly agricultural system. According to [43], connecting digital devices to renewable energy has the potential to improve electricity efficiency, reduce energy consumption in the agricultural sector, leading to reduced operating costs, thereby promoting AGP. However, [4] agrued that the moderation effects of ICT with REC on AVA is negative. The study suggests that developing countries need to have appropriate policies in integrating renewable energy and applying information technology to promote sustainable AGP. Thus, in this research, we examine the interaction effect of REC with ICT on AGP in the Asia. Based on the research results, the authors will provide some policy implications for improving agricultural productivity through the integration of ICT with REC in agricultural practices.

**Hypothesis 3.** The interaction effect of REC with ICT increases AGP in Asian region.

The NR curse has become a concern for the region due to the rapid increase in resource exploitation, leading to negative economic, social and environmental impacts. This theory states that countries with abundant natural resources have more difficulty achieving sustainable economic development, due to over-dependence on resource exploitation [44]. Many studies have examined the role of NR in economic growth. Few studies have evaluated the role of this factor in the agricultural sector. According to [10], NR reduces AGP in the ASEAN nations. [28] indicated that resource windfalls decrease AGP in 38 sub-Saharan Africa countries, confirming the NR curse of agriculture that exists in these nations. However, [45] stated that well-managed natural resources will improve incomes and ensure food security for rural people. In this research, we will add further empirical evidence on the relationship between NR and AGP in the Asian region.

**Hypothesis 4**. NR significantly increases AGP in the Asia.

Financial development (FD) is closely related to agricultural productivity, particularly through the expansion of credit, insurance, and modern payment services for farmers. As the financial system develops, farmers have easier access to capital to invest in machinery, new seeds, and production technologies, thereby improving efficiency and productivity. [46] confirmed that financial development has a positive impact on productivity and economic growth, with agriculture clearly benefiting. Furthermore, [47] demonstrates that agricultural credit from the formal financial system significantly impacts crop productivity, while [48] showed that limited access to credit reduces the production efficiency of smallholder farmers. This evidence reinforces the view that financial development is a crucial driver for improving agricultural productivity and promoting sustainable development. Moreover, some studies show that financial development boosts agricultural productivity [25–27]. However, some studies claimed that financial development reduces agricultural output [23]. Thus, the authors will provide further empirical evidence on this impact, in the case of Asian countries.

**Hypothesis 5**. FD significantly increases AGP in Asian nations.

Good institutional quality plays an important role in promoting sustainable AGP because good institutions create a stable legal framework, encourage investment, allocate resources effectively, resolve disputes and ensure farmers’ rights, thereby helping to balance production, environmental protection and income generation. [49] show the positive impact of governance on AGP, implying that a nation with better institutional quality can create more the agricultural products. However, [50] proposed a contrasting perspective, confirmed that governance hurts the crop production index. Thus, we will provide further empirical evidence on this impact, in the case of Asian countries. The research findings will suggest several policy implications for improving agricultural productivity.

**Hypothesis 6**. IQ significantly increases AGP in Asian nations.

## 4. Results and discussions

### 4.1. Results

Table 2 and Table 3 present descriptive statistics and a correlation matrix between variables in the model. The results of Table 2 indicate that the mean value of AGP is the highest (26.661), followed by ICT, REC, NR, IQ, and FD. The correlation matrix (Table 3) shows that except for natural resources, all other variables are positively correlated with the dependent variable. Moreover, Table 4 shows the variance inflation factor (VIF) used to assess multicollinearity.

**Table 2:** Description of the variables.

Variables	Obs.	Mean	SD	Min	Max
AGP	638	26.661	4.285	18.459	36.652
REC	638	2.213	1.678	−2.302	4.521
ICT	638	16.246	2.562	0	21.282
NR	638	0.443	2.891	−8.684	4.179
FD	638	−1.132	0.633	−3.552	−0.069
IQ	638	−0.472	0.798	−2.124	1.117

**Table 3:** Correlations results.

Variables	AGP	REC	ICT	NR	FD	IQ
AGP	1					
REC	0.155^***^	1				
ICT	0.499^***^	−0.040	1			
NR	−0.387^***^	−0.076^*^	−0.217^***^	1		
FD	0.105^***^	0.100^**^	−0.0417	−0.191^***^	1	
IQ	0.135^***^	−0.154^***^	0.0522	−0.090^**^	0.040	1

***p < 0.01, **p < 0.05,*p < 0.1.

**Table 4:** Multicollinearity test result.

Variables	VIF	1/VIF
NR	1.11	0.901
ICT	1.06	0.942
FD	1.05	0.947
REC	1.04	0.957
IQ	1.04	0.963
**Mean VIF**	1.06	

The average VIF value is below five in Table 4, which shows that there is no multicollinearity phenomenon. We can proceed with the next steps of analysis.

Before performing regression analysis, the stationarity of the variables must be checked to ensure that the results of the regression are reliable. First, we test the cross-sectional dependency (CSD) within the panel data by applying Breusch-Pagan LM, Pesaran scaled LM, Bias-corrected scaled LM, and Pesaran CD tests, and the outcomes are presented in Table 5. The outcomes indicate that the p-values of all variables are significant at the 1% and 10% level, implying that hypothesis H0 is rejected and that CSD exists in the panel data. However, first-generation unit root tests often ignore CSD, which can decrease the reliability of the outcomes. Therefore, second-generation root tests were used [51]. Table 6 presents the test results of each selected variable, showing that all variables are stationary at the first difference, I(1).

**Table 5:** Results of CSD tests.

Variables	AGP	REC	ICT	NR	FD	IQ
Breusch-Pagan LM	6841.72^***^	3534.29^***^	8318.31^***^	2768.72^***^	3509.21^***^	1903.89^***^
Pesaran scaled LM	225.84^***^	109.78^***^	277.66^***^	82.915^***^	108.90^***^	52.56^***^
Bias-corrected scaled LM	225.15^***^	109.09^***^	276.97^***^	82.22^***^	108.21^***^	51.84^***^
Pesaran CD	68.35^***^	4.259^***^	91.15^***^	25.54^***^	31.15^***^	1.71^*^

***p < 0.01, **p < 0.05,*p < 0.1.

**Table 6:** Outcomes from panel unit root test.

Variables	CADF	
	Level	1st difference
AGP	−1.650	−2.678^***^
REC	−0.971	−2.208^***^
ICT	−2.199^***^	−2.822^***^
NR	−1.871	−2.783^***^
FD	−1.747	−2.442^***^
IQ	−2.286^***^	−2.890^***^

***p < 0.01, **p < 0.05,*p < 0.1.

After conducting the CSD and stationarity tests, the authors continue with the next step of testing the long-run association between the variables in the model using the Kao test. The test outcomes in Table 7 indicate that there exists a long-run nexus between the variables. The results confirm a long-term relationship between the research variables. Thereby, FMOLS and DOLS methods were applied. The Driscoll-Kraay standard error (DK)’ approach and the GMM-system estimator were used to check robustness.

**Table 7:** Outcomes for Panel cointegration test.

	t-statistics	p value
ADF	2.451927	0.0071^***^

With FMOLS estimation, in Model I of Table 8, REC and ICT significantly countribute to AGP in Asia. Specifically, a unit increase in REC and ICT leads to 0.458% and 0.766% point increase in AGP, respectively. This implies that REC enables the enhancement of AGP. This research result is also reinforced by the findings of [11], who showed that REC allows enhancing AGP. Therefore, countries need to switch to using renewable energy to replace fossil energy sources for enhancing AGP to aim for sustainable agricultural development. This result is consistent with the findings of [16] for sub-Saharan Africa, [6] for Indonesia. With their wide coverage, ease of use, multi-functionality, and low cost, mobile devices, especially phones, have solved the biggest challenge for farmers in connecting with markets, promoting the commercialization of agricultural products. This helps improve market access efficiency, thereby increasing income for farming households. In terms of natural resources, NR is found to have a significantly negative influence on AGP. This result shows that the resource curse exists in Asia. This phenomenon coincides with the research results of [28] for 38 Sub-Saharan African countries and [10] for ten ASEAN countries. Accordingly, the hypothesis regarding the curse of NR in the agricultural sector was explored. Therefore, to achieve sustainable AGP, governments at all levels are required to implement appropriate policies to protect natural resources and exploit them appropriately for development. FD and IQ are found to have a positive and statistically insignificant relationship with AGP.

**Table 8:** Results from FMOLS and DOLS models.

Variables	FMOLS		DOLS	
	I	II	I	II
REC	0.458^**^	3.510^**^	0.163	6.097^***^
ICT	0.766^***^	1.416^***^	1.264^***^	2.292^***^
REC*ICT		−0.195^**^		−0.349^***^
NR	−0.384^***^	−0.359^***^	−0.277^***^	−0.226^***^
FD	0.435	0.599	0.483^**^	0.688^***^
IQ	0.002	0.002	0.002^***^	0.002^***^

***p < 0.01, **p < 0.05,*p < 0.1.

With DOLS estimation, the results are similar to the FMOLS, REC, ICT, FD and IQ have a positive impact on AGP, whereas NR has the opposite. With the DOLS estimate, FD and IQ significantly contribute to AGP in Asia. Specifically, a unit increase in FD and IQ leads to a 0.483% and 0.002% points increase in AGP, respectively. Our outcome is supported by the findings of [9] in ASEAN-4, who found that FD allows for enhanced AGP, An efficient financial system allows farmers to easily access loans at low interest rates, allowing them to invest in modern technology, transform farming models to be more efficient, thereby promoting AGP. However, the results of this study are contrary to the results of [4], who found that FD negatively affects AGP. Accordingly, Asian countries need to increase the efficiency of the financial system, implement preferential loan interest rates, and create conditions for farmers to access capital for green agricultural investment. In terms of IQ, IQ is positively related to AGP, which implies that IQ allows for the promotion of agricultural development. This implies that IQ enables agricultural development, government with effective institutional quality, together with effective management and regulation enables AGP. This outcome is supported with that of [9], who found that IQ positively affected AGP. However, contrary to the results of [32], who argued that voice and accountability have a significant negative impact on AGP. To increase AGP while reducing emissions in agricultural production, the role of institutional quality is extremely important. Therefore, governments of Asian countries need to enhance government efficiency, listen to the voice of the people to adjust regulations accordingly.

With FMOLS and DOLS estimation, in Model II of Table 8, the moderation effects of ICT with REC are observed to have a significantly negative effect on AGP. This aligns with the findings of [4], which indicate that the moderating effects of ICT with REC on AGP are negative. Therefore, our results show that the combination of REC and ICT has an ineffective impact on agricultural development, which can be explained by barriers such as the lack of comprehensive and synchronous support policies for the application of renewable energy and ICT in agriculture, especially financial mechanisms to encourage investment. In addition, the small and fragmented scale of production in many rural areas is a major barrier to the application of advanced technologies, requiring large investment costs. High-quality human resources for the operation and maintenance of smart agricultural systems are also a challenge, and the problem of data integration is complex, making it difficult for farmers to access and apply. In addition, many modern technological solutions are too complicated, expensive and not suitable for the level and actual needs of farmers, leading to difficulties in implementation and operation. To overcome this, it is necessary to invest in digital infrastructure, build appropriate technology solutions, enhance digital skills training for farmers, have financial policies to support loans and tax incentives, encourage cooperatives and technology enterprises to participate, and at the same time build a smart and sustainable agricultural ecosystem.

### 4.2. Robustness checks

The results of the Wooldridge autocorrelation test and the Modified Wald test confirm the presence of heteroskedasticity and autocorrelation problems in the model (Table 9). The Hausman test result favours the use of the fixed effects model for the empirical analysis. The DSK estimate is suitable for empirical analysis with large cross-sectional dependency (29) and small time periods (22) as in this study, and adequately addresses the issues of heteroskedasticity, autocorrelation, and CSD in the empirical model [52].

**Table 9:** Results of diagnostic tests.

Test statistics	Value
Hausman test	24.06^***^
Wooldridge Autocorrelation test	257.589^***^
Modified Wald test for Heteroskedasticity	11218.61^***^

***p < 0.01.

Using the Driscoll and Kraay estimation to check robustness is presented in Table 10. In the model-I and II of Table 10, the results indicate that REC, ICT, FD and IQ have a significantly positive impact on AGP, while NR has a negative effect on AGP. In the model-II of Table 10, the moderation effects of ICT with REC are observed to have a significantly negative effect on AGP.

**Table 10:** The results of Driscoll and Kraay estimation.

Variables	I	II
REC	−0.083	1.359^***^
ICT	0.287^***^	0.628^***^
REC*ICT		−0.098^***^
NR	−0.049^***^	−0.010
FD	0.846^***^	0.550^***^
IQ	0.000^***^	0.000^***^
Constant	22.886^***^	17.395^***^
Observation	638	638
Countries	29	29

***p < 0.01, **p < 0.05,*p < 0.1.

Table 11 indicates the results for checking robustness by applying GMM estimation to further consolidate the research outcomes. The GMM estimator results illustrate that the lag of the AVA variable is statistically significant, indicating the appropriateness of the GMM estimator outcomes. Additionally, the p-values of AR1, AR2 and Hansen also state the appropriateness of the GMM estimator. The results from the GMM estimator are similar to those of DOLS, FMOLS and the Driscoll and Kraay estimation. The results of Table 11 state that REC, ICT, FD and IQ positively affect AGP, whereas NR and the moderation effects of ICT with REC are observed to have a significantly negative effect on AGP.

**Table 11:** The results of GMM estimation.

Variables	I	II
AGP_t-1_	0.978^***^(0.004)	0.990^***^ (0.005)
REC	0.061^***^(0.007)	0.128^***^(0.041)
ICT	0.042^***^(0.006)	0.036^***^(0.041)
REC*ICT		−0.006^**^(0.002)
NR	−0.024^***^(0.008)	−0.019^***^(0.006)
FD	0.053^*^(0.027)	0.085^**^(0.032)
IQ	0.0001^*^(0.00005)	0.00001(0.00003)
Constant	−0.131(0.097)	−0.201(0.155)
Observation	609	609
AR(1)	−3.59 (0.000)	−3.56(0.000)
AR(2)	−1.64 (0.100)	−1.64 (0.101)
Hansen test	22.97 (0.346)	22.75 (0.301)

***p < 0.01, **p < 0.05,*p < 0.1.

The results of the causality test in Table 12 illustrated a bidirectional causality among FD, and IQ with AGP. This bidirectional causality implies that any change in these variables will significantly affect other variables in Asian countries. Additionally, it is observed that a unidirectional causality runs from ICT to AGP in such Asian countries.

**Table 12:** Pairwise Dumitrescu Hurlin Panel causality tests.

Variables	AGP	REC	ICT	NR	FD	IQ
AGP	**–**	[5.556] (6.491^***^) {0.000}	[2.779] (0.942) {0.346}	[3.981] (3.345^***^) {0.000}	[5.034] (5.449^***^) {0.000}	[3.284] (1.856^*^) {0.063}
REC	[1.944] (−0.725) {0.468}	**–**	[4.433] (4.248^***^) {0.000}	[3.795] (2.972^***^) {0.003}	[4.133] (3.649^***^) {0.000}	[2.669] (0.656) {0.511}
ICT	[5.640] (6.659^***^) {0.000}	[4.422] (4.226^***^) {0.000}	**–**	[4.399] (4.179^***^) {0.000}	[6.496] (8.370^***^) {0.000}	[4.014] (3.279^***^) {0.001}
NR	[2.562] (0.510) {0.609}	[3.415] (2.212^**^) {0.026}	[2.640] (0.664) {0.506}	**–**	[3.565] (2.514^**^) {0.011}	[3.540] (2.355^**^) {0.018}
FD	[3.290] (1.963^**^) {0.049}	[2.978] (1.339) {0.180}	[2.589] (0.563) {0.573}	[3.212] (1.807^*^) {0.070}	–	[3.632] (2.533^**^) {0.011}
IQ	[3.264] (1.816^*^) {0.069}	[2.491] (0.308) {0.757}	[2.688] (0.692) {0.488}	[2.668] (0.654) {0.512}	[2.771] (0.854) {0.392}	**–**

Note: *, **, & *** denote 0.10, 0.05, & 0.01 significance level, respectively. [.], (.) and {.} stands for W-bar, Z-bar, and P-value, respectively.

## 5. Conclusion

### 5.1. Summary of findings

This study analyzes the nexus between renewable energy, information and communication technologies, financial development, natural resources, institutional quality and agricultural productivity in 29 Asian countries for the period of 2000–2021. The empirical outcomes show that all variables are stationary at the first difference I(1), based on the results of the CADF tests. All variables are cointegrated in the long run by the Kao test. The FMOLS, DOLS, DSK and GMM estimation are used for the empirical analysis. The empirical results indicate that renewable energy positively affects AGP, which confirms hypothesis 1. Additionally, hypothesis 2 is also confirmed, the findings show that ICT enhances AGP. However, the finding rejected hypothesis 3, which assumes that the interaction between ICT and REC enhances AGP in Asian countries. The finding rejected hypothesis 4, which assumes that natural resources contribute to AGP. In contrast, the results confirm hypothesis 5, financial development promotes AGP. Similarly, the study`s finding validates the hypothesis 6, institutional quality enhances AGP. Furthermore, causality testing results revealed a bidirectional causal relationship between FD, IQ, and AGP. Additionally, a unidirectional causal relationship from ICT to AGP was observed in these Asian countries.

### 5.2. Policy implications

Based on research results, the suggestions have been made to enhance AGP in Asian countries.

First, renewable energy is found to have a positive influence on AGP. Therefore, governments should encourage the use of renewable energy sources for agricultural production. For example, in some countries, with the potential to produce clean energy from wind power, such as Vietnam, local authorities implement the construction of clean energy production from wind power, which is the process of using wind power as a renewable energy source, converting the kinetic energy of wind into electricity through wind turbines, without emitting greenhouse gases, contributing to reducing pollution and dependence on fossil fuels. Accordingly, this energy source can be used to dry agricultural products, pump water, and provide lighting, such as irrigation and lighting systems for dragon fruit plants. In addition, countries should encourage the production of biomass energy from agricultural by-products and organic waste, which provides heat and electricity for farm operations at farmers’ households.

Second, policy implications related to the application of ICT to enhance AGP. Governments should encourage farmers and cooperatives to apply artificial intelligence to diagnose crop diseases and optimize fertilizer and pesticide use. Because AI can help analyze data from the environment and crops, make predictions, and support decision-making, it can help farmers improve productivity, reduce costs, and promote sustainable agricultural development. For example, information about impending heavy rainfall could help farmers postpone pesticide spraying or harvest crops in a timely manner to minimize losses. Additionally, consideration should be given to using drones for spraying and sowing to optimize agricultural practices. In addition, local authorities should implement policies to encourage farmers to use agricultural robots for weeding, crop care, and harvesting, which could lead to increased productivity. These devices can be operated remotely or automatically, offering convenience and high efficiency for users.

Third, some policy suggestions regarding financial support. Governments and financial institutions should consider providing preferential financial support and loan packages for farmers. Simultaneously, collaboration between technology companies and farmers is also an effective solution to alleviate the financial burden on farmers, as the initial investment costs for technologies applied to agricultural production are quite high. Specifically, the banking system needs to have a policy to reduce interest rates for farmers. This allows farmers to switch to modern farming models, innovate production technology, and increase productivity. Policies to encourage cleaner, low-carbon production need to be considered, prioritizing capital for clean production models to enhance AGP and move towards sustainable agriculture. Moreover, supporting agricultural cooperatives with capital and infrastructure to implement the circular economy. Waste from agricultural production can produce clean energy, which contributes to increase agricultural productivity, while reducing environmental pollution.

Fourth, this result shows that the resource curse exists in Asia. Therefore, to break the “resource curse,” governments should implement policies that shift from exploitation-based growth to efficiency- and knowledge-based growth. For example, applying tax incentives and green credit mechanisms for businesses investing in deep processing, precision agriculture, and seed technology, in order to escape the trap of exporting cheap raw materials.

Furthermore, the policy implications relate to institutional quality. Governments should refine the legal framework to encourage investment in wind, solar, and biomass energy for agricultural production and processing. Furthermore, governments should create transparent grid connection mechanisms to allow farmers to sell surplus electricity. In addition, it is necessary to develop rural digital infrastructure, standardize data systems and traceability, and encourage the application of IoT, AI, and blockchain in production management, thereby enhancing the efficiency and transparency of the value chain. Financially, governments should expand rural credit, develop agricultural insurance and green financial products, and encourage the mobilization of private and international capital through green bonds and public-private partnerships. These institutional reforms will lay the foundation for a modern agricultural ecosystem, moving towards green and sustainable agriculture.

### 5.3. The limitations of study

Although this study finds evidence of the nexus between renewable energy, information and communication technologies, financial development, natural resources, institutional quality and agricultural productivity in 29 Asian countries, it still has a few limitations. Future research could extend the investigation of the effects of climatic conditions on AGP for all Asian countries. For example, consider the impact of rainfall and temperature on agricultural productivity in this region. In this study, the authors only considered Voice and Accountability as a proxy for institutional quality, further research could consider six indicators such as Control of Corruption, Political Stability, Regulatory Quality, Government Effectiveness, Rule of Law and Voice and Accountability, and use principal component analysis to determine the value of institutional quality variables. Furthermore, the role of human resources should also be considered in relation to AGP. Because the high quality human resources can help farmers update new knowledge and modern technology more easily, thereby helping to improve agricultural productivity. Therefore, future research can incorporate these variables in relation to AGP, adding causal analysis between variables, providing policy makers with more perspectives for the goal of green agricultural growth.
