# Supplementary material for: Renewable energy, information and communication technology, financial development, natural resources, institutional quality, and agricultural productivity in Asian countries
Source: PLoS One. 2026 Aug 14;21(8):e0355560. doi: 10.1371/journal.pone.0355560 (PMC13475886; doi:10.1371/journal.pone.0355560)
Supplement: Supplementary file 1 [file pone.0355560.s001.docx]

**The relationship between renewable energy, information and communication technologies, financial development and sustainable agriculture: Evidence from Asia nations**

Tinh Do Phu Tran^1,2*^, Hue Trinh Hoang Hong^1,2*^, Duyen Thi My Thi ^1,2*^

^1^University of Economics and Law, Ho Chi Minh City, Vietnam

^2^Vietnam National University, Ho Chi Minh City, Vietnam

**Corresponding author:* [*duyenttm21702@sdh.uel.edu.vn*](mailto:duyenttm21702@sdh.uel.edu.vn) *(Duyen Thi My Thi)*

[*tinhdpt@uel.edu.vn*](mailto:tinhdpt@uel.edu.vn) *(Tinh Tran Phu Do)*

[*huethh@uel.edu.vn*](mailto:huethh@uel.edu.vn) *(Hue Trinh Hoang Hong)*

**ABSTRACT**

This study investigates the effect of renewable energy consumption (REC), information and communication technologies (ICT), natural resources (NR), financial development (FD) and institutional quality (IQ) on agricultural add valued (AVA) in 29 Asian countries for the 2000 – 2021 period. The fully modified least-squares (FMOLS) and dynamic least-squares (DOLS) estimation are used for the empirical analysis. The Driscoll and Kraay estimation (DSK) and the generalised method of moments (GMM) are used for robustness checks. The empirical results indicate that REC, ICT, FD, IQ have a significant positive impact on AVA, whereas NR has a significant negative influence on AVA, implying that the resource curse persists in Asian countries. The results of the causality test illustrated a bidirectional causality among FD, and IQ with AVA. Considering the research results, the authors suggest some policies to encourage the use of clean energy for agriculture, integrate ICT in agricultural production, and provide capital incentives for green projects, aiming at sustainable agricultural growth for Asian countries.

**Keywords:** Renewable energy, natural resources, ICT, financial development, sustainable agriculture.

**1. Introduction**

Climate change is threatening agricultural production, and thus, the livelihoods and food security of billions of people who depend on agriculture in the Asian region. Agriculture is highly vulnerable to climate change because of its dependence on climate and weather. According to statistics, Asia accounts for approximately 37% of greenhouse gas emissions from agricultural production and China accounts for more than 18% of total emissions^[1]^. Ensuring food security, increasing the value of agricultural products, and reducing carbon emissions from the agricultural sector are all necessary to make agriculture more sustainable. Innovation in production practices and the implementation of comprehensive mitigation and adaptation strategies are needed to meet the food and income needs of current and future generations.

In Asia, agriculture plays an important role. More than 60% of the population, about 2.2 billion people in this region, depend on agriculture for their livelihoods, while the contribution of agriculture to the gross domestic product (GDP) is declining across the region from an average of 11.146% in 2000 to 7.103% in 2023^[2]^. In 2019, the agricultural sector accounted for approximately 2 percent of total energy consumption in Southeast Asian countries. Energy consumption in this sector increased by 35 percent between 2009 and 2019^[3]^. This negatively impacts the environment. Therefore, increasing access to clean, affordable, and environmentally friendly energy is essential to promote sustainable agriculture in the region. In addition, reducing food loss and waste is another way to reduce harmful impacts on the environment. The presence of large agricultural industries creates large amounts of unused wastes. According to Amran et al. (2021), the agricultural industry generates approximately 1,300 million tons of waste each year, and approximately 50% of the raw materials are discarded without being processed. This process is wasteful and harmful to the environment. Agricultural waste (fruits, vegetables, and plant waste) can be reused to produce biomass energy (Selvaggi and Valenti 2021). Agriculture plays a dual role as an energy user and supplier in the form of bioenergy^[4]^. Several studies have examined the role of renewable energy consumption (REC) in sustainable agricultural development (Wang et al. 2023). Some studies have shown bidirectional causality between REC and agricultural value added (AVA) (Qamruzzaman 2022; Chopra et al. 2022). Countries are committed to achieving sustainable development goals, by halving emissions by 2023, and moving towards zero emissions by 2050. Therefore, raising awareness among farmers in using renewable energy in agricultural production is extremely important. Because consuming renewable energy can help reduce emissions (Thi et al. 2023). Integrating information technology into agricultural production is extremely important, it allows farmers to better adapt to climate change through more modern production models. This study examines the relationship between renewable energy, natural resources, information and communication technologies, financial development and agriculture development in Asian countries.

This study contributes to the literature in several ways. Although previous studies have assessed the relationship between agricultural value added (AVA) and its determinants for various regions. There is still a lack of research for the case of Asian countries. Specifically, studies have not yet emphasized the decisive role of REC and ICT on sustainable agriculture in Asia. Therefore, this study will examine the role of ICT in using Mobile to share knowledge, access weather-related information to help farmers make accurate decisions to improve productivity, promote sustainable agriculture (Mariyono et al. 2022). Furthermore, the use of renewable energy sources for agriculture is also urgent. Because it helps reduce emissions from agricultural production activities, allowing agricultural products to be more environmentally friendly, towards sustainable agriculture (Wang et al. 2023). Additionally, this study also incorporates the interactive effects of renewable energy and ICT on AVA. Moreover, financial resources are considered to be extremely important for farmers to invest in agricultural production activities, it allows farmers to invest in technological knowledge related to ICT and renewable energy, which is beneficial for the goal of sustainable agriculture. Furthermore, as the increasing consumption of fossil energies and overexploitation of natural resources pose a threat to the environment, many studies have investigated the role of natural resources for economic growth. However, few studies have examined the role of this factor in the agricultural sector. This study will provide further evidence on the direct impact of natural resources on AVA, thereby suggesting some policy implications towards sustainable development goals (SDGs), specifically target 2.4.

The authors use recent econometric methods to analyze the model. First, we test the cross-sectional dependence (CSD). Because if the research skips this step, the results may be distorted (Halkos and Polemis 2017). If CSD exists in the panel data, the second-generation root tests will apply, as well as panel cointegration tests are performed. The fully modified least-squares (FMOLS) and dynamic least-squares (DOLS) methods will be applied. the Driscoll-Kraay standard error (DK)’ approach and the generalised method of moments (GMM)-system estimator will be used for check robustness.

The structure of this study is as follows: Section 2 provides a brief overview of the literature; Section 3 presents the methodology and data; Section 4 illustrates the outcomes and discussions; and Section 5 concludes the paper.

**2. Literature review**

**2.1 Renewable energy and agriculture sector**

The world is facing a major challenge of climate change, partly caused by carbon emissions. To counter this risk, renewable energy should be considered for its contribution to sustainable development goals (SDGs) for global countries. Several studies have examined the role of renewable energy consumption (REC) in sustainable agricultural development. For instance, Wang et al. (2023) examined the link between REC and agriculture productivity (AP) in 123 nations during the period 1995-2019. They show that REC, foreign direct investment (FDI), financial inclusion have a postive influence on AP, whereas carbon dioxide emission (CO_2_E) show the opposite. Research suggests that farmers should limit the consumption of non-renewable energy sources for agricultural production and switch to use renewable energy. Qamruzzaman (2022) investigated the effect of renewable energy (RE) and FDI on AP and found that RE and FDI positively affect AP. The outcomes also indicate that the existence of bidirectional causality between RE and AP, between FDI and AP in lower-income and upper-middle-income nations. Similarly, Nendissa et al. (2022) examined the role of RE and Non-RE on Agricultural Economics (AE) in Indonesia from to 1986-2020. The outcomes revealed that renewable energy production (REP), REC, and non-REC positively affected AE. In additionally, Soni and Rl (2024) investigated the link between AP and REC in BRICS from 2000 to 2020. The outcomes indicate that REC, CO2E, and FDI positively affect AP, whereas access to financial inclusion has no impact on AP. Furthermore, Chandio et al. (2022) assessed the influence of climate change and financial development (FD) on agricultural production (AP) in ASEAN-4 from to 1990-2016. They found that climate change had a negative impact on agricultural production, whereas renewable energy, human capital (HC), and institutional quality (IQ) positively affected AP. Chopra et al. (2022) evaluated the impact of REC and NR on agricultural productivity in ASEAN countries. They found that REC positively affected the agricultural sector, whereas the forest area, CO2E, and NR negatively affected AP. Similarly, Karimi Alavijeh and Salehnia (2023) investigated the nexus between climate change, REC, non-REC, and agricultural development in Middle East and North African nations during 2000-2018. They found that temperature negatively affected AVA, whereas precipitation showed the opposite effect. In addition, REC increases AVA, whereas non-REC reduces it. This study suggests that governments should target the development of renewable energy sources, particularly in the agricultural sector.

**2.2 Natural resources and agriculture sector**

The demand for fossil energy is increasing to serve economic growth, which leads to overuse of natural resources and also causes large emissions. Recently, natural resources and environmental pollution have attracted much attention from researchers. Much of the literature focuses on the impact of natural resources on economic growth, emissions, and poverty. Few studies have examined the impact of natural resources on the agricultural sector. Chopra et al. (2022) examined the effects of CO_2_E, deforestation, REC, GF, and regional integration on agricultural productivity (AP) in ten ASEAN countries. The empirical outcomes show that CO_2_E, forest area, and NR decrease agricultural productivity, while renewable energy consumption (REC) increases agricultural productivity. Similarly, Dorinet et al. (2021) explored that resource windfalls reduce AP in Sub-Saharan Africa, confirming the resource curse of agriculture in these countries. In the case of 25 oil-exporting nations, Abdlaziz et al. (2018) studied the impact of oil prices on agriculture and showed that oil prices have a negative impact on agricultural value-added. Implying that that Dutch disease exists in these countries. In another research, Apergis et al. (2014) examined the impact of oil rents on AVA in the Middle East and North Africa (MENA) nations. They discovered a negative link between oil rents and AVA, showing that the oil industry boom is associated with a contraction in agriculture. Likewise, Zhou et al. (2024) discovered a negative nexus between NR abundance and agricultural productivity (AP) in Asia-Pacific Economic Cooperation (APEC) countries, implying that natural resource decreases AP.

**2.3 Information and communication technologies (ICT) and agriculture sector**

The effective use of ICT to improve access to appropriate agricultural information and services holds great promise for transforming farmers' productivity and livelihoods. Oyelami et al. (2022) examined the efect of ICT on agriculture sector performances (ASP) in sub-Saharan Africa (SSA) for period of 1995–2017 by employing panel autoregressive distributed lag (ARDL) approach. The results show that ICT has positive impact on ASP in the long run. However, there is no evidence that this effect persists in the short term. Similarly, Sethi et al. (2024) examined the effect of renewable energy (RE), ICT and agricultural credit (AC) for sustainable agricultural sector in developing nations for the 2000-2021 period by emloying the Driscoll-Kraay and Quantile Regression for check robustness. They indicate that RE, ICT and AC have a positive impact on agriculture sector. The moderating effect of the interaction between ICT and renewable energy is negative and weak. Mariyono et al. (2022) investigated the mobile phones use to support agricultural business management in Indonesia. The results show that mobile phone usage allows farmers to enhance sales, get market information, access to advanced agricultural technology. This helps increase profits for farmers. The study suggests encouraging farmers to use mobile phones to access information for agricultural business activities. Similar results were also found in the study of Erlangga et al. (2023); Rajkhowa and Qaim (2022). According to Baumüller (2022) provide opportunities to improve the delivery of services to farmers in developing nations. Mobile networks are increasingly popular, making them easily accessible to users, which makes it easier for farmers to collect and share agricultural knowledge with each other. Similarly, according to Uy et al. (2023), mobile phones allow farmers to easily exchange market information, receive weather information, receive agricultural extension advice, and learn about new farming technologies, which allows them to improve agricultural productivity.

**2.4 Financial development (FD) and agriculture sector**

Investing financial resources for agricultural development is necessary, it allows farmers to convert to a more modern agricultural production model by applying modern technology, it helps the agricultural sector increase productivity and product quality. The important role of financial development in economic development has been proven by many studies, its role in the agricultural sector also needs to be clarified. Anetor et al. (2016) indicated that commercial loans to the farming sector significantly affect production in the country. Accordingly, the study suggests that commercial banks should support low-interest loans to farmers. Additionally, Zakaria et al. (2019) examined the effect of financial development on agricultural productivity in South Asia and found the inverted U-shaped result of FD on agricultural production. Similarly, Chandio et al. (2022) examined the influence of climate change and FD on agricultural production in ASEAN-4 for the 1990-2016 period. They found that climate change affects agricultural production (AP) negatively, while FD improves AP only after reaching a certain threshold. They suggest the governments must adopt flexible financial and agricultural policies so that farmers would be benefitted and AP can be increased. On the contrary, according to Liu et al. (2021) digital inclusive finance reduce households' agricultural output. Similar results were also found in the study of Sethi et al. (2024), who found that FD and political stability affect agriculture negatively. However, Chandio et al. (2021) examined the influence of global climate change, financial development (FD), and technical progress on cereal production in Pakistan from 1977 to 2014. They found that CO2 emissions negatively affect on cereal production, whereas FD and technical progress have a positive influence on cereal production. Research suggests that financial development is necessary for sustainable agriculture.

2.5 Research gap

Although previous studies have assessed the relationship between agricultural value added (AVA) and its determinants for various regions. There is still a lack of research for the case of Asia. This study provides further empirical evidence on the impact of renewable energy, natural resources, information and communication technology (ICT), and financial development (FD) on AVA in this region. Specifically, studies have not yet emphasized the decisive role of REC and ICT on sustainable agriculture. Therefore, this study will examine the role of ICT in using Mobile to share knowledge, access weather-related information to help farmers make accurate decisions to improve productivity, promote sustainable agriculture (Mariyono et al. 2022). Furthermore, the use of renewable energy sources for agriculture is also urgent. Because it helps reduce emissions from agricultural production activities, allowing agricultural products to be more environmentally friendly, towards sustainable agriculture (Wang et al. 2023). Additionally, this study also incorporates the interactive effects of renewable energy and ICT on AVA. Moreover, the study also examines the role of financial development for AVA. Financial resources are considered to be extremely important for farmers to invest in agricultural production activities, it allows farmers to invest in technological knowledge related to ICT and renewable energy, which is beneficial for the goal of sustainable agriculture. Furthermore, as the increasing consumption of fossil energy and overexploitation of natural resources pose a threat to the environment, many studies have investigated the role of NR for economic growth. However, few studies have examined the role of this factor in the agricultural sector. This study will provide further evidence on the direct impact of natural resources on AVA, thereby suggesting some policy implications towards sustainable development goals (SDGs), specifically target 2.4.

**3. Methodology and data**

**3.1 Data and variables**

This study uses a panel dataset of 29 Asian countries, with data ranging from 2000 to 2021. Data is collected from World Bank and International Monetary Fund (IMF). In this research, agricultural value-added (AVA) is the dependent variable, and renewable energy consumption (REC), information and communication technologies (ICT), natural resources (NR), financial development (FD) and institutional quality (IQ) are explanatory variables. Table 1 provides details of all the variables and their sources.

**Table 1:** Describe variables

| **Variables** | **Measurement** | **Data Source** |
| --- | --- | --- |
| Agriculture (AVA) | Log of agriculture, forestry, and fishing, value added (current LCU) | WDI |
| Renewable energy consumption (REC) | Renewable energy consumption (% of total final energy consumption) | WDI |
| Information and communication technologies (ICT) | Log of mobile cellular subscriptions | WDI |
| Natural  resources (NR) | Log of total natural resources rents (% of GDP) | WDI |
| Financial development (FD) | Log of financial development index | IMF |
| Institutional quality (IQ) | Voice and Accountability | WDI |

**3.2 Theoretical framework and study hypothesis**

The factors influencing agricultural production are presented based on the production function, where productivity is expressed in terms of functional inputs. Theoretically, agricultural productivity depends on capital (K) and labor (L). However, according to Echevarria (1998)The production function can be expanded using more input variables. The nexus between inputs and outputs in agricultural production can be presented based on the Cobb–Douglas production function, as follows:

Y_it_ = θK_it_^α^ L_it_^β^ E_it_^є^	(1).

Where Y is output, is measured by agricultural value-added (AVA), K is capital, and L is labor, and E is other input. This study examined the effect of renewable energy consumption (REC), information and communication technologies (ICT), natural resources (NR), financial development (FD) and institutional quality (IQ) on agriculture. When we calculate the natural logarithm of equation (1) and incorporate renewable energy consumption (REC), information and communication technologies (ICT), natural resources (NR), financial development (FD) and institutional quality (IQ)in the model, equation (1) becomes

${lnAVA}_{i,t}=\alpha_{i,t}+{lnREC}_{i,t}+{lnICT}_{i,t}+{lnNR}_{i,t}{+{lnFD}_{i,t}+{lnIQ}_{i,t}+\varepsilon}_{i,t}$ (2)

Furthermore, Equation (2) is widened by introducing the interaction of (LnREC * LnICT) to examine the moderation influence of REC with ICT on AVA in equation (3).

${lnAVA}_{i,t}=\alpha_{i,t}+{lnREC}_{i,t}+{lnICT}_{i,t}+lnREC*lnICT+{lnNR}_{i,t}{+{lnFD}_{i,t}+{lnIQ}_{i,t}+\varepsilon}_{i,t}$ (3)

Asian countries face great challenges from climate change, which affects agricultural production and threatens national food security. Creating agricultural value-added (AVA) while reducing emissions is the path towards sustainable agricultural development and green growth. The agricultural sector in developing countries plays an important role in creating jobs and ensuring national food security. On the other hand, this sector is also responsible for climate change, about 37% of greenhouse gas emissions from agricultural production in Asian countries. Therefore, to achieve the goal of sustainable agricultural development, it is necessary to use environmentally friendly inputs. According to Wang et al. (2023) REC allows for increased agricultural productivity, and it also helps reduce CO2E (Thi and Do 2024). Converting fossil energy consumption to renewable energy in agricultural production to create more environmentally friendly products, meeting export market demand with high quality, low emission agricultural product standards. Thus, the authors assume the effect of REC on AVA to be positive, i.e.$\frac{{\beta AVA}_{it}}{{\beta REC}_{it}}$>0, in hypothesis 1. Similarly, the main benefits that ICT brings to agriculture are enhancing information, increasing productivity, improving management capacity, proactively seeking markets, reducing risks, and creating a foundation for developing new business models. Therefore, the author believes that the impact of ICT on agriculture is positive, i.e., $\frac{{\beta AVA}_{it}}{{\beta ICT}_{it}}$>0, in hypothesis 2. Furthermore, over the past few decades, the theory of the NR curse has become a worrying factor for the region due to the rapid increase in resource exploitation (Sovacool 2010). Many studies have evaluated the role of natural resources in economic growth. Few studies have investigated the role of this factor in the agricultural sector. According to Chopra et al. (2022) NR decrease agricultural productivity in the ASEAN countries. Likewise, Dorinet et al. (2021) show that resource windfalls reduce AP in 38 sub-Saharan Africa, confirming the resource curse of agriculture that exists in these countries. However, well-managed natural resources will contribute to improved incomes and food security for rural people (Bakala and Asfaw 2020). Therefore, we assume the impact of NR on AVA to be positive, i.e., $\frac{{\beta AVA}_{it}}{{\beta NR}_{it}}$>0, in hypothesis 3. Moreover, financial investment is essential for farmers to convert to more modern farming models, applying modern science and technology to improve productivity and product quality. A healthy financial system with preferential interest rates will create opportunities for farmers to access capital for investment in production in a modern, environmentally friendly direction. Therefore, the author believes that FD has a positive influence on AVA, $\frac{{\beta AVA}_{it}}{{\beta FD}_{it}}$>0, in hypothesis 4. Similarly, Otim et al. (2023) confirmed that governance has a negative impact on the crop production index. However, Lio and Liu (2008), proposed a contrasting perspective, showing the positive impact of governance on AVA, implying that a nation with better institutional quality can create more AVA. Thus, the authors assume the impact of IQ on AVA to be positive, i.e., $\frac{{\beta AVA}_{it}}{{\beta IQ}_{it}}$>0, in hypothesis 5. Additionally, In addition, connecting digital devices to renewable energy has the potential to improve electricity efficiency, reduce energy consumption in the agricultural sector, leading to reduced operating costs (Karlilar et al. 2023). Therefore, the study evaluates the interaction between ICT and REC for AVA, which is considered positive in hypothesis 6.

4. Results and Discussions

4.1 Results

Table 2 and Table 3 present descriptive statistics and correlation matrix between variables in the model. The results of Table 2 indicate that the mean value of AVA is the highest (26.661), followed by ICT, REC, NR, IQ, and FD. The correlation matrix (Table 3) shows that except for natural resources, all other variables are positively correlated with the dependent variable. Moreover, Table 4 shows the variance inflation factor (VIF) used to assess multicollinearity.

**Table 2:** Description of the variables

| **Variables** | **Obs.** | **Mean** | **SD** | **Min** | **Max** |
| --- | --- | --- | --- | --- | --- |
| AVA | 638 | 26.661 | 4.285 | 18.459 | 36.652 |
| REC | 638 | 2.213 | 1.678 | -2.302 | 4.521 |
| ICT | 638 | 16.246 | 2.562 | 0 | 21.282 |
| NR | 638 | 0.443 | 2.891 | -8.684 | 4.179 |
| FD | 638 | -1.132 | 0.633 | -3.552 | -0.069 |
| IQ | 638 | -0.472 | 0.798 | -2.124 | 1.117 |

Table 3: Correlations results

| **Variables** | **AVA** | **REC** | **ICT** | **NR** | **FD** | **IQ** |
| --- | --- | --- | --- | --- | --- | --- |
| AVA | 1 |  |  |  |  |  |
| REC | 0.155^***^ | 1 |  |  |  |  |
| ICT | 0.499^***^ | -0.040 | 1 |  |  |  |
| NR | -0.387^***^ | -0.076^*^ | -0.217^***^ | 1 |  |  |
| FD | 0.105^***^ | 0.100^**^ | -0.0417 | -0.191^***^ | 1 |  |
| IQ | 0.135^***^ | -0.154^***^ | 0.0522 | -0.090^**^ | 0.040 | 1 |

****p < 0.01, **p < 0.05 ,*p < 0.1*

The average VIF value is below five in Table 4, which shows that there is no multicollinearity phenomenon. We can proceed with the next steps of analysis.

Table 4 Multicollinearity test result

| **Variables** | **VIF** | **1/VIF** |
| --- | --- | --- |
| NR | 1.11 | 0.901 |
| ICT | 1.06 | 0.942 |
| FD | 1.05 | 0.947 |
| REC | 1.04 | 0.957 |
| IQ | 1.04 | 0.963 |
| Mean VIF | 1.06 |  |

Before performing regression analysis, the variables stationarity must be checked to ensure that the results of the regression are reliable. First, we test the cross-sectional dependency (CSD) within the panel data by applying Breusch-Pagan LM, Pesaran scaled LM, Bias-corrected scaled LM, and Pesaran CD tests, and the outcomes are presented in Table 5. The outcomes indicate that the p-values of all variables are significant at the 1% and 10% level, implying that hypothesis H0 is rejected and that CSD exists in the panel data. However, first-generation unit root tests often ignore CSD, which can decrease the reliability of the outcomes. Therefore, second-generation root tests were used (Pesaran 2007). Table 6 shows the test results of each selected variable and indicates that all variables are stationary at the first difference, I(1).

Table 5: Results of CSD tests

| **Variables** | **AVA** | **REC** | **ICT** | **NR** | **FD** | **IQ** |
| --- | --- | --- | --- | --- | --- | --- |
| Breusch-Pagan LM | 6841.72^***^ | 3534.29^***^ | 8318.31^***^ | 2768.72^***^ | 3509.21^***^ | 1903.89^***^ |
| Pesaran scaled LM | 225.84^***^ | 109.78^***^ | 277.66^***^ | 82.915^***^ | 108.90^***^ | 52.56^***^ |
| Bias-corrected  scaled LM | 225.15^***^ | 109.09^***^ | 276.97^***^ | 82.22^***^ | 108.21^***^ | 51.84^***^ |
| Pesaran CD | 68.35^***^ | 4.259^***^ | 91.15^***^ | 25.54^***^ | 31.15^***^ | 1.71^*^ |

****p < 0.01, **p < 0.05 ,*p < 0.1*

**Table 6:** Outcomes from panel unit root test

| **Variables** | **CADF** | |
| --- | --- | --- |
|  | **Level** | **1st difference** |
| AVA | -1.650 | -2.678^***^ |
| REC | -0.971 | -2.208^***^ |
| ICT | -2.199^***^ | -2.822^***^ |
| NR | -1.871 | -2.783^***^ |
| FD | -1.747 | -2.442^***^ |
| IQ | -2.286^***^ | -2.890^***^ |

****p < 0.01, **p < 0.05 ,*p < 0.1*

After conducting the CSD and stationarity tests, The authors continue with the next step of testing the long-run association between the variables in the model using the Kao test. The test outcomes in Table 7 indicate that there exists the long-run nexus between the variables. The results confirm a long-term relationship between the research variables. Thereby, the fully modified least-squares (FMOLS) and dynamic least-squares (DOLS) methods will be applied. the Driscoll-Kraay standard error (DK)’ approach and the generalised method of moments (GMM)-system estimator will be used for check robustness.

Table 7: Outcomes for Panel cointegration test

|  | t-statistics | p value |
| --- | --- | --- |
| ADF | 2.451927 | 0.0071^***^ |

Table 8: Results from FMOLS and DOLS models

| **Variables** | **FMOLS** | | DOLS | |
| --- | --- | --- | --- | --- |
|  | I | II | I | II |
| REC | 0.458^**^ | 3.510^**^ | 0.163 | 6.097^***^ |
| ICT | 0.766^***^ | 1.416^***^ | 1.264^***^ | 2.292^***^ |
| REC*ICT |  | -0.195^**^ |  | -0.349^***^ |
| NR | -0.384^***^ | -0.359^***^ | -0.277^***^ | -0.226^***^ |
| FD | 0.435 | 0.599 | 0.483^**^ | 0.688^***^ |
| IQ | 0.002 | 0.002 | 0.002^***^ | 0.002^***^ |

****p < 0.01, **p < 0.05 ,*p < 0.1*

With FMOLS estimation, in Model I of Table 8, REC and ICT significantly countribute to agricultural value added in Asia. Specifically, a unit increase in REC and ICT lead to 0.458% and 0.766% points increase in AVA, respectively. This implies that REC enables the enhancement of agricultural value added. This research result is also reinforced by the findings of Karimi Alavijeh and Salehnia (2023), who showed that REC allow enhance AVA. Therefore, countries need to switch to using renewable energy to replace fossil energy sources towards sustainable agriculture. This result is consistent with the findings of Oyelami et al. (2022) for sub-Saharan Africa, Mariyono et al. (2022) for Indonesia. With widespread coverage, ease of use, multi-functionality and low cost, mobile devices, especially phones, have solved the biggest challenge for farmers in connecting to the market, promoting the commercialization of agricultural products. This helps improve agricultural value added. In term of natural resources, NR is found to have a significantly negative influence on AVA. This result shows that the resource curse exists in Asia. This phenomenon coincides with the research results of Dorinet et al. (2021) for 38 Sub-Saharan African countries and Chopra et al. (2022) for ten ASEAN countries. Accordingly, the hypothesis regarding the curse of NR in the agricultural sector was explored. Therefore, to achieve sustainable agriculture, governments at all levels are required to implement appropriate policies to protect natural resources and exploit them appropriately for development. FD and IQ are found to have a positive and statistically insignificant relationship with AVA. With DOLS estimation, the results are similar with the FMOLS, REC, ICT, FD and IQ have a positive impact on AVA, whereas NR has the opposite. With DOLS estimate, FD and IQ significantly countribute to agricultural value added in Asia. Specifically, a unit increase in FD and IQ leads to a 0.483% and 0.002% points increase in AVA, respectively. our outcome is supported by the findings of Chandio et al. (2022) in ASEAN-4, who found that FD allows enhance AVA, an efficient financial system allows farmers to easily access loans at low interest rates, allowing them to invest in modern technology, transform farming models to be more efficient, thereby promoting AVA. However, the results of this study are contrary to the results of Sethi et al. (2024), who found that FD negatively affect agriculture. Accordingly, Asian countries need to increase the efficiency of the financial system, implement preferential loan interest rates, and create conditions for farmers to access capital for green agricultural investment. In term of IQ, IQ is positively related to AVA, this implies that IQ allows for the promotion of agricultural development. This implies that IQ enables agricultural development, government with effective institutional quality, together with effective management and regulation enable agricultural value addition. This outcome is supported with that of Chandio et al. (2022), who found IQ positively affected AVA. However, contrary to the results of Gelgo, Gemechu, and Bedemo (2023), who argued that voice and accountability has a negative significant impact on AVA. To increase agricultural added value while reducing emissions in agricultural production, the role of institutional quality is extremely important. Therefore, governments of Asian countries need to enhance government efficiency, listen to the voice of the people to adjust regulations accordingly.

With FMOLS and DOLS estimation, in Model II of Table 8, the moderation effects of ICT with REC is observed to have a significantly negative effect on AVA. This aligns with conclusions drawn by Sethi et al. (2024), who found that the moderation effects of ICT with REC on AVA is negative. Asian governments need to increase farmers' awareness of renewable energy use, by encouraging farmers to adopt information technology to share and acquire knowledge in adapting to climate change and improving agricultural productivity.

**4.2 Robustness checks**

**Table 9:** Results of diagnostic tests

| **Test statistics** | **Value** |
| --- | --- |
| Hausman test | 24.06^***^ |
| Wooldridge Autocorrelation test | 257.589^***^ |
| Modified Wald test for Heteroskedasticity | 11218.61^***^ |

****p < 0.01*

The results of Wooldridge autocorrelation test and Modified Wald test confirm the presence of heteroskedasticity and autocorrelation problems in the model (Table 9). The Hausman test result favours the use of fixed effects model for the empirical analysis. The DK estimate is suitable for empirical analysis with large cross-sectional dependency (29) and small time periods (22) as in this study, and adequately addresses the issues of heteroskedasticity, autocorrelation, and CSD in the empirical model (Behera et al. 2024).

**Table 10:** The results of Driscoll and Kraay estimation

| **Variables** | I | II |
| --- | --- | --- |
| REC | -0.083 | 1.359^***^ |
| ICT | 0.287^***^ | 0.628^***^ |
| REC*ICT |  | -0.098^***^ |
| NR | -0.049^***^ | -0.010 |
| FD | 0.846^***^ | 0.550^***^ |
| IQ | 0.000^***^ | 0.000^***^ |
| Constant | 22.886^***^ | 17.395^***^ |
| Observation | 638 | 638 |
| Countries | 29 | 29 |

****p < 0.01, **p < 0.05 ,*p < 0.1*

Using the Driscoll and Kraay estimation for check robustness is presented in Table 10. In the model-I and II of Table 10, the results indicate that REC, ICT, FD and IQ have a significantly positive impact on AVA, while NR has a negative effect on AVA. In the model-II of Table 10, the moderation effects of ICT with REC is observed to have a significantly negative effect on AVA.

Table 11: The results of GMM estimation

| **Variables** | I | II |
| --- | --- | --- |
| AVA_t-1_ | 0.978^***^(0.004) | 0.990^***^ (0.005) |
| REC | 0.061^***^(0.007) | 0.128^***^(0.041) |
| ICT | 0.042^***^(0.006) | 0.036^***^(0.041) |
| REC*ICT |  | -0.006^**^(0.002) |
| NR | -0.024^***^(0.008) | -0.019^***^(0.006) |
| FD | 0.053^*^(0.027) | 0.085^**^(0.032) |
| IQ | 0.0001^*^(0.00005) | 0.00001(0.00003) |
| Constant | -0.131(0.097) | -0.201(0.155) |
| Observation | 609 | 609 |
| AR(1) | -3.59 (0.000) | -3.56(0.000) |
| AR(2) | -1.64 (0.100) | -1.64 (0.101) |
| Hansen test | 22.97 (0.346) | 22.75 (0.301) |

****p < 0.01, **p < 0.05 ,*p < 0.1*

Table 11 indicates the results for check robustness by applying GMM estimation to further consolidate the research outcomes. The GMM estimator results illustrates that the lag of the AVA variable is statistically significant, indicating the appropriateness of the GMM estimator outcomes. Additionally, the p-values of AR1, AR2 and Hansen also states the appropriateness of the GMM estimator. The results from GMM estimator are similar to those of DOLS, FMOLS and the Driscoll and Kraay estimation. The results of Table 11 state that REC, ICT, FD and IQ positively affect AVA, whereas NR and the moderation effects of ICT with REC is observed to have a significantly negative effect on AVA.

The results of the causality test in Table 12 illustrated a bidirectional causality among FD, and IQ with AVA. This bidirectional causality implies that any change in these variables will significantly affect other variables in Asian countries. Additionally, it is observed that a unidirectional causality running from ICT to AVA in such Asian countries.

**Table 12**: Pairwise Dumitrescu Hurlin Panel Causality Tests

| **Variables** | AVA | REC | ICT | NR | FD | IQ |
| --- | --- | --- | --- | --- | --- | --- |
| AVA | **-** | [5.556]  (6.491^***^)  {0.000} | [2.779]  (0.942)  {0.346} | [3.981]  (3.345^***^)  {0.000} | [5.034]  (5.449^***^)  {0.000} | [3.284]  (1.856^*^)  {0.063} |
| REC | [1.944]  (-0.725)  {0.468} | **-** | [4.433]  (4.248^***^)  {0.000} | [3.795]  (2.972^***^)  {0.003} | [4.133]  (3.649^***^)  {0.000} | [2.669]  (0.656)  {0.511} |
| ICT | [5.640]  (6.659^***^)  {0.000} | [4.422]  (4.226^***^)  {0.000} | **-** | [4.399]  (4.179^***^)  {0.000} | [6.496]  (8.370^***^)  {0.000} | [4.014]  (3.279^***^)  {0.001} |
| NR | [2.562]  (0.510)  {0.609} | [3.415]  (2.212^**^)  {0.026} | [2.640]  (0.664)  {0.506} | **-** | [3.565]  (2.514^**^)  {0.011} | [3.540]  (2.355^**^)  {0.018} |
| FD | [3.290]  (1.963^**^)  {0.049} | [2.978]  (1.339)  {0.180} | [2.589]  (0.563)  {0.573} | [3.212]  (1.807^*^)  {0.070} | - | [3.632]  (2.533^**^)  {0.011} |
| IQ | [3.264]  (1.816^*^)  {0.069} | [2.491]  (0.308)  {0.757} | [2.688]  (0.692)  {0.488} | [2.668]  (0.654)  {0.512} | [2.771]  (0.854)  {0.392} | **-** |

*Note: *, **, & *** denote 0.10, 0.05, & 0.01 significance level, respectively. [.], (.) and {.} stands for W-bar, Z-bar, and P-value, respectively.*

5. Conclusion

5.1. Conclusion

This study analyzes the effect of renewable energy, information and communication technologies, natural resources, financial development and institutional quality on agriculture in 29 Asian countries for the period of 2000 - 2021. The empirical outcomes show that all variables are stationary at the first difference I(1), based on the results of the CADF tests**.** All variables are cointegrated in the long run by the Kao test. The FMOLS, DOLS, DSK and GMM estimation are used for the empirical analysis. The empirical results indicate that renewable energy is found to have a positive influence on agriculture, which confirms the hypothesis 1 of this research. Additionally, the hypothesis 2 also gets confirm, the findings show that ICT enhance AVA. It implies that ICT enables farmers to capture timely information, access markets, manage supplies, thereby improving AVA. However, the finding rejected hypothesis 3, which assumes that natural resources contribute to AVA. on the other hand, the research results confirm hypothesis 4, financial development promotes AVA. Similarly, the study`s finding validates the hypothesis 5, institutional quality enhances AVA. However, the finding rejected hypothesis 6, which assumes that the interaction between ICT and REC enhances AVA in Asian countries.

5.2. Policy implications

Based on research results, the results have considered making some suggestions to enhance agricultural added value in Asian countries. First, renewable energy is found to have a positive influence on agriculture. Thereby, farmers can integrate renewable energy into agriculture to increase added value. The governments can encourage investments in REC to promote the green agricultural sector. This enables high-quality agricultural production with low-carbon emissions. Investment policies for green projects should be prioritized. Second, the banking system needs to have a policy to reduce interest rates for farmers. A policy to reduce loan interest rates to create opportunities for farmers to access capital. This allows farmers to switch to modern farming models, innovate production technology, and increase productivity. Third, there needs to be strict policies and legal frameworks to implement digital transformation and integrate ICT tools in agricultural cultivation to improve agricultural productivity. Furthermore, policies to encourage cleaner, low-carbon production need to be considered, prioritizing capital for clean production models to move towards sustainable agriculture. Moreover, supporting agricultural cooperatives with capital and infrastructure to implement the circular economy, raising awareness, and promoting circular economic development is necessary. Waste from agricultural production can produce clean energy, which contributes to increasing the value of agricultural products, while reducing environmental pollution. the results show that a resource curse exists in Asian countries; therefore, the government needs to have strict regulations on the exploitation and management of natural resources. Finally, to achieve sustainable development goals, specifically goal 2.4, national governments need to issue integrated policies, increase farmers' awareness of using renewable energy, and combine ICT applications in agricultural production to adapt to climate change.

5.3. The limitations of study

Although this study finds evidence of the effect of renewable energy, information and communication technologies, natural resources, financial development and institutional quality on agriculture in 29 Asian countries, it still has a few limitations. Future research could extend the investigation of the effects of climatic conditions on AVA. In addition, the role of human resources should also be considered in relation to AVA. Because the high quality human resources can help farmers update new knowledge and modern technology more easily, thereby helping to improve agricultural productivity. Therefore, future research can incorporate these variables in relation to AVA, adding causal analysis between variables, providing policy makers with more perspectives for the goal of green agricultural growth.

**Disclosure statement:** No potential conflict of interest was reported by the author(s).

**Data availability statement:** All data related to the study are given in tables and figures in the manuscript. However, if more data is required, it will be provided by the corresponding author.

**Acknowledgements**: This research was funded by Vietnam National University Ho Chi Minh City (VNU-HCM) under grant number B2025-34-03.

**Author contribution**

**Tinh Tran** **Phu Do** contributed significantly to the research process by providing valuable research methodology and data analysis, drafting the initial manuscript, making critical revisions, revising the final manuscript according to the reviewer`s request, and accepting the final version.

**Hue Trinh Hoang Hong** focused on developing the introduction, writing the literature review, assisting in data collection, and interpreting the findings, revised the final manuscript according to the reviewer`s request, and accepted the final version.

**Duyen Thi** focused on providing valuable research methodology and data analysis, drafting the initial manuscript, making critical revisions, revising the final manuscript according to the reviewer`s request, and accepting the final version.

**References**

Abdlaziz, Rizgar Abdlkarim, Mohd Naseem Niaz Ahmad, and Ly Slesman. 2018. "Dutch disease effect of oil price on agriculture sector: evidence from panel cointegration of oil exporting countries." *International Journal of Energy Economics and Policy* 8 (5):241-250.

Anetor, Friday, Chris Ogbechie, Ikechukwu Kelikume, and Fredrick Ikpesu. 2016. "Credit supply and agricultural production in Nigeria: a vector autoregressive (VAR) approach." *Journal of Economics and Sustainable Development* 7 (2).

Apergis, Nicholas, Ghassen El-Montasser, Emmanuel Sekyere, Ahdi N. Ajmi, and Rangan Gupta. 2014. "Dutch disease effect of oil rents on agriculture value added in Middle East and North African (MENA) countries." *Energy Economics* 45:485-490. doi: <https://doi.org/10.1016/j.eneco.2014.07.025>.

Bakala, Fayera, and Milkessa Asfaw. 2020. "The role of natural resources for sustaining food security in Ethiopia: a review." *Food Science and Quality Management* 97 (2224-6088):5-13.

Baumüller, Heike. 2022. "Towards smart farming? Mobile technology trends and their potential for developing country agriculture." In *Handbook on ICT in developing countries*, 191-210. River Publishers.

Behera, Puspanjali, Biswanath Behera, and Narayan Sethi. 2024. "Assessing the impact of fiscal decentralization, green finance and green technology innovation on renewable energy use in European Union countries: What is the moderating role of political risk?" *Renewable Energy* 229:120715. doi: <https://doi.org/10.1016/j.renene.2024.120715>.

Chandio, Abbas Ali, Yuansheng Jiang, Waqar Akram, Sultan Adeel, Muhammad Irfan, and Inayatullah Jan. 2021. "Addressing the effect of climate change in the framework of financial and technological development on cereal production in Pakistan." *Journal of Cleaner Production* 288:125637. doi: <https://doi.org/10.1016/j.jclepro.2020.125637>.

Chandio, Abbas Ali, Muhammad Ibrahim Shah, Narayan Sethi, and Zulqarnain Mushtaq. 2022. "Assessing the effect of climate change and financial development on agricultural production in ASEAN-4: the role of renewable energy, institutional quality, and human capital as moderators." *Environmental Science and Pollution Research* 29 (9):13211-13225. doi: 10.1007/s11356-021-16670-9.

Chopra, Ritika, Cosimo Magazzino, Muhammad Ibrahim Shah, Gagan Deep Sharma, Amar Rao, and Umer Shahzad. 2022. "The role of renewable energy and natural resources for sustainable agriculture in ASEAN countries: Do carbon emissions and deforestation affect agriculture productivity?" *Resources Policy* 76:102578. doi: <https://doi.org/10.1016/j.resourpol.2022.102578>.

Dorinet, Elizavetta, Pierre-André Jouvet, and Julien Wolfersberger. 2021. "Is the agricultural sector cursed too? Evidence from Sub-Saharan Africa." *World Development* 140:105250. doi: <https://doi.org/10.1016/j.worlddev.2020.105250>.

Echevarria, Cristina. 1998. "A Three-Factor Agricultural Production Function: The Case of Canada." *International Economic Journal* 12 (3):63-75. doi: 10.1080/10168739800000029.

Erlangga, Erlangga, Owen Machuku, and Clint Jun Dahino. 2023. "A review article on the impact and challenges of mobile phone usage on agricultural production in Africa." *Cogent Food & Agriculture* 9 (2):2273634.

Gelgo, Biru, Adeba Gemechu, and Amsalu Bedemo. 2023. "The effect of institutional quality on agricultural value added in East Africa." *Heliyon* 9 (10). doi: 10.1016/j.heliyon.2023.e20964.

Halkos, George E, and Michael L Polemis. 2017. "Does financial development affect environmental degradation? Evidence from the OECD countries." *Business Strategy and the Environment* 26 (8):1162-1180. doi: <https://doi.org/10.1002/bse.1976>.

Karimi Alavijeh, Nooshin, and Narges Salehnia. 2023. "Climate change, renewable and non-renewable energy consumption and agricultural development in the Middle East and North African countries." *Environment, Development and Sustainability*. doi: 10.1007/s10668-023-03521-5.

Karlilar, Selin, Mehmet Balcilar, and Firat Emir. 2023. "Environmental sustainability in the OECD: The power of digitalization, green innovation, renewable energy and financial development." *Telecommunications Policy* 47 (6):102568. doi: <https://doi.org/10.1016/j.telpol.2023.102568>.

Lio, Monchi, and Meng-Chun Liu. 2008. "Governance and agricultural productivity: A cross-national analysis." *Food Policy* 33 (6):504-512. doi: <https://doi.org/10.1016/j.foodpol.2008.06.003>.

Liu, Yang, Chunyu Liu, and Mi Zhou. 2021. "Does digital inclusive finance promote agricultural production for rural households in China? Research based on the Chinese family database (CFD)." *China Agricultural Economic Review* 13 (2):475-494.

Mariyono, Joko, Siswanto Imam Santoso, Jaka Waskito, and Akbar Ario Satrio Utomo. 2022. "Usage of mobile phones to support management of agribusiness activities in Indonesia." *Aslib Journal of Information Management* 74 (1):110-134. doi: 10.1108/AJIM-02-2021-0053.

Nendissa, Doppy Roy, Atiek Iriany, Jeky Melkianus Sui, Nikmatul Khoiriyah, Onuma Suphattanakul, and Worakamol Wisetsri. 2022. "The role of renewable and nonrenewable energy on agricultural economics in Indonesia." *International Journal of Energy Economics and Policy* 12 (3):352-360.

Otim, Jacob, Susan Watundu, John Mutenyo, Vincent Bagire, and Muyiwa S. Adaramola. 2023. "Effects of carbon dioxide emissions on agricultural production indexes in East African community countries: Pooled mean group and fixed effect approaches." *Energy Nexus* 12:100247. doi: <https://doi.org/10.1016/j.nexus.2023.100247>.

Oyelami, Lukman O, Nurudeen Afolabi Sofoluwe, and Omowumi Monisola Ajeigbe. 2022. "ICT and agricultural sector performance: empirical evidence from sub-Saharan Africa." *Future Business Journal* 8 (1):18.

Pesaran, M Hashem. 2007. "A simple panel unit root test in the presence of cross‐section dependence." *Journal of applied econometrics* 22 (2):265-312. doi: <https://doi.org/10.1002/jae.951>.

Qamruzzaman, Md. 2022. "Nexus between renewable energy, foreign direct investment, and agro-productivity: The mediating role of carbon emission." *Renewable Energy* 184:526-540. doi: <https://doi.org/10.1016/j.renene.2021.11.092>.

Rajkhowa, Pallavi, and Matin Qaim. 2022. "Mobile phones, off‐farm employment and household income in rural India." *Journal of Agricultural Economics* 73 (3):789-805. doi: <https://doi.org/10.1111/1477-9552.12480>.

Selvaggi, Roberta, and Francesca Valenti. 2021. "Assessment of fruit and vegetable residues suitable for renewable energy production: GIS-based model for developing new frontiers within the context of circular economy." *Applied System Innovation* 4 (1):10. doi: <https://doi.org/10.3390/asi4010010>.

Sethi, Litu, Puspanjali Behera, Biswanath Behera, and Narayan Sethi. 2024. "Unravelling the role of renewable energy, information and communication technology and agricultural credit for sustainable agricultural productivity in developing countries." *International Journal of Sustainable Development & World Ecology* 31 (7):989-1003. doi: 10.1080/13504509.2024.2366474.

Soni, Shnehal, and Manogna Rl. 2024. "The dynamic nexus between agricultural productivity and renewable energy consumption in BRICS: the role of financial inclusion and foreign direct investment." *International Journal of Energy Sector Management* ahead-of-print (ahead-of-print). doi: 10.1108/IJESM-08-2023-0028.

Sovacool, Benjamin K. 2010. "The political economy of oil and gas in Southeast Asia: heading towards the natural resource curse?" *The Pacific Review* 23 (2):225-259. doi: 10.1080/09512741003624484.

Thi, Duyen My-Thi, and Tran Tinh-Phu Do. 2024. "The interrelationships between carbon dioxide emissions and innovation: international evidence." *International Journal of Sustainable Energy* 43 (1):2352515. doi: 10.1080/14786451.2024.2352515.

Thi, Duyen, Van Q. Tran, and Dat T. Nguyen. 2023. "The relationship between renewable energy consumption, international tourism, trade openness, innovation and carbon dioxide emissions: international evidence." *International Journal of Sustainable Energy* 42 (1):397-416. doi: 10.1080/14786451.2023.2192827.

Uy, Tran Cao, Hoang Dung Ha, Nguyen Ngoc Truyen, Nguyen Van Chung, Duong Ngoc Phuoc, Le Van Nam, Le Thi Hong Phuong, and Cao Thi Thuyet. 2023. "Mobile phone use for farm-related activities by ethnic minority farmers during the Covid-19 pandemic in Quang Tri Province, Central Vietnam." *Information Services and Use* 43 (1):27-37.

Wang, Yanqi, Mansoora Ahmed, Syed Ali Raza, and Maiyra Ahmed. 2023. "Threshold nonlinear relationship between renewable energy consumption and agriculture productivity: the role of foreign direct investment and financial inclusion." *Environmental Science and Pollution Research* 30 (24):65900-65915. doi: 10.1007/s11356-023-27003-3.

Zakaria, Muhammad, Wen Jun, and Marium Farrukh Khan. 2019. "Impact of financial development on agricultural productivity in South Asia." *Agricultural Economics/zemědělská Ekonomika* 65 (5).

Zhou, Zhongmin, Arshian Sharif, Roula Inglesi-Lotz, and Muhammad Farhan Bashir. 2024. "Analysing the interplay between energy transition, resource consumption, deforestation, and environmental factors on agricultural productivity: Insights from APEC countries." *Journal of Cleaner Production* 446:141408. doi: <https://doi.org/10.1016/j.jclepro.2024.141408>.

1. https://www.adb.org/sites/default/files/publication/27531/building-climate-resilience-agriculture-sector.pdf
2. https://databank.worldbank.org/source/world-development-indicators#
3. https://www.irena.org/media/Files/IRENA/Agency/Publication/2022/Jun/IRENA_Renewables_Agriculture_SEAsia_2022.pdf
4. https://www.adb.org/sites/default/files/publication/684966/adb-brief-173-green-recovery-southeast-asia.pdf
