# Supplementary material for: Renewable energy, information and communication technology, financial development, natural resources, institutional quality, and agricultural productivity in Asian countries
Source: PLoS One. 2026 Aug 14;21(8):e0355560. doi: 10.1371/journal.pone.0355560 (PMC13475886; doi:10.1371/journal.pone.0355560)
Supplement: Supplementary file 2 [file pone.0355560.s002.docx]

Dear Editor,

I am writing to provide my review of the manuscript titled “The relationship between renewable energy, information and communicationtechnologies, financial development and sustainable agriculture: Evidence from Asia nations”. The manuscript examines the impact of renewable energy consumption, information and communication technologies, financial development, natural resources, and institutional quality on agricultural value added in 29 Asian countries between 2000 and 2021. Using advanced econometric models (FMOLS, DOLS, DSK, and GMM), the study finds that renewable energy, ICT, financial development, and institutional quality support sustainable agriculture, while natural resources exert a negative “resource curse” effect. The paper highlights policy recommendations to promote clean energy adoption, ICT integration, and financial incentives for green agricultural practices in Asia.

While the manuscript introduces a technically innovative framework with promising results, I believe that several revisions could improve its clarity, interpretability, and impact. Below, I offer my detailed comments and recommendations:

1. **Title**

- Consider a smoother phrasing to improve readability.
- “Asia nations” in the current version is grammatically awkward; use “Asian nations.”

2. **Abstract:**

- Revise grammar and clarity: e.g., “agricultural add valued (AVA)” → “agricultural value added (AVA).”
- The phrase “illustrated a bidirectional causality among FD, and IQ with AVA” could be streamlined: “illustrated bidirectional causality between FD, IQ, and AVA.”
- Suggest adding a novelty statement (e.g., how this study differs from existing literature in Asia).

3. **Introduction:**

- Improve flow by condensing overly long paragraphs.
- Recheck factual clarity: the sentence “Countries are committed to halving emissions by 2023” seems inconsistent with international agreements (likely 2030).
- Some citations are heavily descriptive—consider synthesizing instead of listing.
- Highlight more explicitly what is new in this study compared to prior ASEAN, BRICS, or MENA analyses.
- Tighten narrative to emphasize research gap and contribution earlier. Replace raw URLs with proper citation formatting.

4. **Literature review:**

- Well-structured by themes (renewable energy, natural resources, ICT, financial development). Good coverage of recent and regional studies.
- Some redundancy (several studies described with similar outcomes). The section is long and reads like a summary rather than a critical synthesis.
- Condense by merging similar findings. Add a clearer positioning of how this study fills the identified gap.

5. **Methodology and Data:**

- Data sources (World Bank, IMF) are credible. Variables are clearly defined in a table. The theoretical framework uses Cobb–Douglas function, which is appropriate.
- Hypotheses are numerous but not clearly linked to each part of the model. Measurement of “institutional quality” with only “voice and accountability” may not fully capture governance dimensions.
- Justify variable choices (especially IQ proxy). Clarify novelty in methodological approach compared to prior studies.

6. **Results and Discussions:**

- Results are presented systematically (descriptive stats, correlation, multicollinearity, stationarity, cointegration, FMOLS/DOLS, robustness with DSK and GMM, causality tests). Interpretations are well connected to literature.
- Some tables are very detailed but under-discussed (e.g., causality test results). The negative interaction of ICT and REC is noted but not deeply explained. Discussion sometimes repeats results instead of analyzing implications.
- Expand discussion on why ICT*REC interaction is negative in Asia (possible issues of technology adoption, infrastructure, cost?). Reduce redundant restatements of results.

7. **Conclusion:**

- Clearly summarizes empirical results and confirms/rejects hypotheses. Policy implications are relevant (green energy, ICT integration, financial support).
- Conclusion partly overlaps with policy implications; readability suffers. Limitations are only briefly addressed at the end.
- Separate Conclusion (summary of findings) from Policy Implications (recommendations). Expand Limitations (e.g., only 29 Asian countries, exclusion of climatic variables, single proxy for governance).

8. **References:**

- Extensive and recent (up to 2024). Includes regional studies relevant to Asia.
- Some formatting inconsistencies (missing page numbers, inconsistent DOI presentation). Raw URLs in the text should be replaced with formatted references.
- Revise references to ensure journal-required style (PLOS ONE prefers consistent DOI format).
