# Supplementary material for: Renewable energy, information and communication technology, financial development, natural resources, institutional quality, and agricultural productivity in Asian countries
Source: PLoS One. 2026 Aug 14;21(8):e0355560. doi: 10.1371/journal.pone.0355560 (PMC13475886; doi:10.1371/journal.pone.0355560)
Supplement: Supplementary file 3 [file pone.0355560.s003.docx]

Comments to the Author

For the manuscript PONE-D-25-41598 entitled ‘’ The relationship between renewable energy, information and communication technologies, financial development and sustainable agriculture: Evidence from Asia nations which has been submitted to PLOS ONE journal .

Thank you for submitting this manuscript to the PLOS ONE journal. Overall, the work is informative, well-organized, and clearly structured, though some sections require minor improvements.

Comment on the use of abbreviations: once you introduce an abbreviation, please use only the abbreviation thereafter. There is no need to repeat the full term with the abbreviation in parentheses each time. This issue appears throughout your manuscript, so please correct it.

2.2 Natural resources and agriculture sector

under this subtitle there is a sentence it says ‘’ Zhou et al. (2024) discovered a negative nexus between NR abundance and agricultural productivity (AP) in Asia-Pacific Economic Cooperation (APEC) countries, implying that natural resource decreases AP’’. How this could be true?

Methodology section

The Methodology section is written in the future tense, but since the work has already been completed, it should be presented in the past tense. Therefore, this section requires revision.
