# Supplementary material for: Renewable energy, information and communication technology, financial development, natural resources, institutional quality, and agricultural productivity in Asian countries
Source: PLoS One. 2026 Aug 14;21(8):e0355560. doi: 10.1371/journal.pone.0355560 (PMC13475886; doi:10.1371/journal.pone.0355560)
Supplement: Supplementary file 4 [file pone.0355560.s004.docx]

**PONE-D-25-41598**

**The relationship between renewable energy, information and communication technologies, financial development and sustainable agriculture: Evidence from Asia nations**

**Journal: PLOS ONE**

**Dear Editor and the Reviewers,**

We would like to thank you for your comments and suggestions. Following your recommendations, we have made several revisions in response to the comments, to strengthen the arguments throughout the paper. Our detailed responses are shown below, with your comments in *italics and* bold, while our responses are given in the normal text.

**Reviewer: 1**

*(1) Despite the statistics and econometric model applied to process data and produce result, the variables that were associated to have effect on one another seems unrelated. For instance, renewable energy is included in the natural resources. But both appeared to be two different variables. The relationship between the selected variables such as renewable energy, information and communication technologies, financial development and sustainable agriculture all seems to have no logical relationship. For example, the authors can see the effect of ICT on Agriculture production; or the effect of financial capacity of farmers on Agriculture productivity.....or the summative effect of "the three" variables on Agriculture Value Addition (if this is considered as dependent variable). But now, the variables related seems to have spurious correlation unless split and stand alone by themselves to be regressed over "one selected dependent variable".*

*The title states "the relationship between renewable energy, information and communication technologies, financial development and sustainable agriculture" ---- Sustainable Agriculture*

🡪Thank you for your comment, the authors have revised the manuscript according to your suggestions.

Burning fossil fuels releases large amounts of carbon dioxide (CO2) into the atmosphere, which is the main cause of the greenhouse effect and rising global temperatures. In 2019, the agricultural sector accounted for approximately 2 percent of total energy consumption in Southeast Asian countries. Energy consumption in this sector increased by 35 percent between 2009 and 2019 (3). This causes serious environmental degradation by releasing greenhouse gases that cause global warming, acid rain (from sulfur dioxide and nitrogen oxide), and air, water, and soil pollution. Countries are committed to achieving sustainable development goals, by halving emissions by 2030, and moving towards zero emissions by 2050. Therefore, innovation in agricultural production activities is urgent to realize the goal of reducing emissions, increasing agricultural productivity, ensuring food security and income for current and future generations, towards the goal of sustainable development. According to (4) renewable energy helps increase agricultural productivity (AGP) by providing a clean, stable source of energy for activities such as irrigation, drying agricultural products, lighting, and operating modern equipment, thereby reducing production costs, improving efficiency and promoting sustainable agricultural development. Therefore, considering the impact of renewable energy consumption (REC) on agricultural development in the Asian region is urgent in the current context. Renewable energy enhances sustainable AGP by reducing operating costs, providing clean, reliable energy for agricultural activities, and minimizing environmental impacts. Sources such as solar, wind, and biomass power help run machinery, pump water, treat waste, and reduce dependence on fossil fuels, contributing to climate change response. Furthermore, information and communication technology (ICT) plays a revolutionary role in the agricultural sector, helping to improve productivity and product quality, optimize resource use, save costs, expand market access channels and support decision-making, thanks to applications such as the Internet of Things (IoT), artificial intelligence (AI), and data analysis, towards a smart and sustainable agriculture. According to (5) ICT increases AGP because it helps optimize production processes and increase resource efficiency. Renewable energy provides clean, economical electricity for agricultural activities, while ICT provides monitoring and automatic control solutions, optimizes resource use and enhances adaptability to climate change, contributing to building a modern, efficient and sustainable agricultural system. Integrating ICT into agricultural production might allow farmers to better adapt to climate change through more modern production models. Moreover, the application of technology helps optimize resource use, increase productivity and reduce greenhouse gas emissions in agricultural production, while financial and institutional development plays an important role in investing, coordinating and creating a favorable legal corridor for the application of these technologies, thereby improving agricultural productivity in a sustainable manner (6). This study examines the relationship between REC, natural resources, ICT, financial development and agriculture productivity in Asian countries. From the research results, the authors will suggest some policy implications towards the goal of sustainable development in the region.

*(2) The Abstract says, "each variable has positive/ negative effect on Agriculture Value Addition" not sustainable agriculture like in the title.*

🡪 Thank you for your comment, the authors have revised the title and content of the manuscript "agricultural productivity".

*The analysis (statistics) processed data to link each variable. But finally arrived on the conclusion that each variable has relationship on Agriculture. This is a vague conclusion*

🡪 Thank you for your comment, the authors have revised the manuscript according to your suggestions.

*(3) Here, the dependent variable is not clearly stated. Is it;*

*- Sustainable agriculture (as on the title) or*

*- Agriculture Value Addition (as in the abstract and in the result) or*

*- Agriculture (as in the conclusion)*

🡪Thank you for your comment, the authors re-aggregated the dependent variable as “agricultural productivity” for both title and content.

*(4) In the theoretical framework, agriculture input and out put (in terms of production) was considered.*

*Whichever the dependent variable is, the three are different concepts that can't be synonymized. The question for the authors is, have they measured the dependent variable in terms of output (if agriculture production) or Value (if AVA) or made trend analysis (if Sustainable agriculture production was considered).*

🡪Thank you for your comment, the authors re-aggregated the dependent variable as “agricultural productivity” for both title and content.

**3.2 Theoretical framework and study hypothesis**

The factors influencing agricultural production are presented based on the production function, where productivity is expressed in terms of functional inputs. Theoretically, agricultural productivity depends on capital (K) and labor (L). However, according to (36), the production function can be expanded using more input variables. The nexus between inputs and outputs in agricultural production can be presented based on the Cobb–Douglas production function was developed by Echevarria (36) as follows:

Y_it_ = θK_it_^α^ L_it_^β^ E_it_^є^	(1).

Where Y is output, is measured by AGP, K is capital, and L is labor, and E is other input. This study examined the effect of REC, ICT, NR, FD and IQ on AGP. When we calculate the natural logarithm of equation (1) and incorporate REC, ICT, NR, FD and IQ, following the studies (33); (34) as mentioned in Equation (2).

${lnAGP}_{i,t}=\alpha_{i,t}+{lnREC}_{i,t}+{lnICT}_{i,t}+{lnNR}_{i,t}{+{lnFD}_{i,t}+{IQ}_{i,t}+\varepsilon}_{i,t}$ (2)

Furthermore, Equation (2) is widened by introducing the interaction of (LnREC * LnICT) to examine the moderating influence of REC with ICT on AGP , following the studies (33), (5) as mentioned in Equation (3).

${lnAGP}_{i,t}=\alpha_{i,t}+{lnREC}_{i,t}+{lnICT}_{i,t}+lnREC*lnICT+{lnNR}_{i,t}{+{lnFD}_{i,t}+{IQ}_{i,t}+\varepsilon}_{i,t}$ (3)

*(5) Generally, the variable relationships are not logically justified. The Literature review part doesn't support the logical relationship of the four variables and the theoretical framework was less related to the empirical studies in the review part.*

🡪Thank you for your comment, the authors have revised the literature review as per your suggestion, it is highlighted in red in the literature review.

**2. Literature review**

**2.1 Renewable energy and** **the agricultural productivity**

The world is facing a major challenge of climate change, partly caused by carbon emissions. To counter this risk, renewable energy should be considered for its contribution to Sustainable Development Goals (SDGs) for global countries. Several studies have examined the role of REC in enhancing sustainable agricultural productivity. For instance, (4) examined the link between REC and AGP in 123 nations during the period 1995-2019. They show that REC, foreign direct investment (FDI), and financial inclusion has a positive influence on AGP, whereas carbon dioxide emission (CO_2_E) show the opposite. Research suggests that farmers should limit the consumption of non-renewable energy sources for agricultural production and switch to enewable energy. Additionally, (11) investigated the link between AGP and REC in BRICS from 2000 to 2020. The outcomes indicate that REC, CO2E, and FDI positively affect AGP, whereas access to financial inclusion has no impact on AGP. (9) assessed the influence of climate change and FD on agricultural production in ASEAN-4 from 1990-2016. They found that climate change hurt gricultural production, whereas renewable energy, human capital (HC), and institutional quality (IQ) positively affected AGP. (10) evaluated the impact of REC and NR on AGP in ASEAN countries. They found that REC positively affected the AGP, whereas the forest area, CO2E, and NR negatively affected AGP. Similarly, (12) found that REC increases AGP in Middle East and North African nations. This study suggests that governments should target the development of renewable energy sources, particularly in the agricultural sector. REC boosts agricultural productivity was also found in the study of (15); (16); (8); (5) and (7).

Generally, some studies examined the nexus between REC and sustainable agricultural development, the outcomes of this influence in Asia still need to be clarified. RE can power irrigation systems, agricultural drying, helping farmers use water and land resources more efficiently. This study will provide further empirical evidence on the impact of REC on AGP in Asian countries. Futhermore, clean energy helps reduce agriculture's carbon footprint, while ICT provides digital solutions to optimize agricultural production and management. However, few studies have examined the interactive effects of REC and ICT on AGP. Considering this interaction is necessary because REC and ICT can promote sustainable AGP by reducing pollution, increasing resource efficiency and creating smarter production systems, thereby improving competitiveness and ensuring food security. RE provides clean power for smart agricultural equipment, such as using solar energy to operate soil sensors and automatic irrigation systems. Thus, the authors also examine the interactive effects of REC and ICT on AGP in Asian countries, thereby suggesting policy implications to promote sustainable agriculture in this region.

**2.2 Natural resources and the agricultural productivity**

The demand for fossil energy is increasing to serve economic growth, which leads to the overuse of natural resources and also causes large emissions. Recently, natural resources and environmental pollution have attracted much attention from researchers. Much of the literature focuses on the impact of natural resources on economic growth, emissions, and poverty. Few studies have examined the impact of natural resources on the agricultural sector. (10) show that CO_2_E, forest area, and NR decrease AGP. Similarly, (17) explored that resource windfalls reduce AGP in Sub-Saharan Africa, confirming the resource curse of agriculture in these countries. In the case of 25 oil-exporting nations, (18) studied the impact of oil prices on agriculture and showed that oil prices harmed on AGP. Implying that Dutch disease exists in these countries. In another research, (19) examined the impact of oil rents on AGP in the Middle East and North Africa (MENA) nations. They discovered a negative link between oil rents and AGP, showing that the oil industry boom is associated with a contraction in agriculture. Likewise, (20) discovered a negative nexus between NR abundance and AGP in Asia-Pacific Economic Cooperation (APEC) countries, implying that natural resource decreases AGP. This confirms the existence of a resource curse in these countries, due to an over-concentration in the resource sector, leading to the neglect of other sectors, increasing dependence on commodity price fluctuations. Poor governance, corruption and political instability also exacerbate this situation, preventing resources from being used effectively to promote sustainable growth.

In summary, many studies have examined the role of NR in economic growth, but few studies have examined the impact of NR on agriculture sector, particularly in Asia. Some studies have found that NR has a negative impact on AGP (10); (20). The authors will provide further empirical evidence on this relationship in Asian countries, thereby suggesting some policy implications for promoting sustainable AGP.

**2.3 Information and communication technologies (ICT) and the agricultural productivity**

The effective use of ICT to improve access to appropriate agricultural information and services holds great promise for transforming farmers' productivity and livelihoods. (21) examined the effect of ICT on agriculture sector performances (ASP) in sub-Saharan Africa (SSA) for the period of 1995–2017 by employing panel autoregressive distributed lag (ARDL) approach. The results show that ICT has a positive impact on ASP in the long run. However, there is no evidence that this effect persists in the short term. Similarly, (5) examined the effect of RE, ICT and agricultural credit (AC) for the sustainable AGP in developing nations for the 2000-2021 period by employing the Driscoll-Kraay and Quantile Regression to check robustness. They indicate that ICT have a positive impact on AGP. The moderating effect of the interaction between ICT and renewable energy is negative and weak. (13) investigated the mobile phones to support agricultural business management in Indonesia. The results show that mobile phone usage allows farmers to enhance sales, get market information, and acces to advanced agricultural technology. This helps increase profits for farmers. The study suggests encouraging farmers to use mobile phones to access information for agricultural business activities. According to (22), mobile networks are increasingly popular, making them easily accessible to users, which makes it easier for farmers to collect and share agricultural knowledge with each other. Similarly, according to (23), mobile phones enable farmers to easily exchange market information, receive weather updates, obtain agricultural extension advice, and learn about new farming technologies, thereby enhancing agricultural productivity. Similar results were also found in the study of (24); (25).

In short, some studies have investigated the impact of ICT on AGP, but this nexus still needs to be clarified in the Asian region. In addition, this study also examines the interaction effect of REC and ICT on AGP. Thereby, the authors will suggest some policy implications for the goal of sustainable AGP in the region for the coming time.

**2.4 Financial development (FD) and the agricultural productivity**

Investing financial resources for agricultural development is necessary, it allows farmers to convert to a more modern agricultural production model by applying modern technology, which helps the agricultural sector increase productivity and product quality. The important role of financial development in economic development has been proven by many studies, its role in the agricultural sector also needs to be clarified. (26) indicated that commercial loans to the farming sector significantly affect production in the country. Accordingly, the study suggests that commercial banks should support low-interest loans to farmers. Additionally, (27) examined the effect of financial development on AGP in South Asia and found the inverted U-shaped result of FD on agricultural production. Similar results were also found in the study of (9) for 4 ASEAN nations. They suggest that governments must adopt flexible financial and agricultural policies so that farmers can benefit and AGP can be increased. On the contrary, according to (28) digital inclusive finance reduces households' agricultural output. However, (29) examined the influence of global climate change, financial development (FD), and technical progress on cereal production in Pakistan from 1977 to 2014. They found that CO2 emissions negatively affect cereal production, whereas FD and technical progress have a positive influence on cereal production. Research suggests that financial development is necessary for sustainable AGP. Similar results were also found in the study of (30); (31); (32), they showed that financial development enhances AGP.

In general, the important role of financial development in economic development has been proven by many studies, its role in the agricultural sector also needs to be clarified. Some studies show that financial development boosts AGP, however some studies claim that financial development reduces AGP (28). The authors will provide further empirical evidence on this impact, in the case of Asian countries. Thereby, propose some policy implications to promote sustainable AGP through financial instruments.

**2.5 Research gap**

Although previous studies have assessed the relationship between AGP and its determinants for various regions. There is still a lack of research on the case of Asia. This study provides further empirical evidence on the impact of REC, NR, ICT, FD and IQ on AGP in this region. Specifically, studies have not yet emphasized the decisive role of REC and ICT in AGP. Therefore, this study will examine the role of ICT in using Mobile to share knowledge, access weather-related information to help farmers make accurate decisions to improve productivity, promote sustainable AGP (13). Furthermore, REC plays an important role in enhancing sustainable AGP. Because it helps reduce emissions from agricultural production activities, allowing agricultural products to be more environmentally friendly, towards sustainable agriculture (4). The interaction between REC and ICT can have a positive impact on AGP, as they can help reduce environmental impacts, increase production efficiency and improve climate change adaptation. ICT plays a supporting role in the effective deployment and management of renewable energy, such as energy monitoring systems, while renewable energy provides clean electricity to operate smart agricultural technologies, creating a more modern and sustainable agricultural ecosystem. However, there is currently a lack of research examining this interaction on agricultural production in Asian regions. Through this study, we will provide more empirical evidence on this interaction. From there, the authors will suggest some policy implications to enhance AGP in the region. Moreover, financial resources are considered to be extremely important for farmers to invest in agricultural production activities, they allow farmers to invest in technological knowledge related to ICT and renewable energy, which is beneficial for the goal of sustainable agriculture. As the increasing consumption of fossil fuels and overexploitation of natural resources pose a threat to the environment, many studies have investigated the role of natural resources in promoting economic growth. However, few studies have examined the role of this factor in the agricultural sector. This study will provide further evidence on the direct impact of natural resources on AGP, thereby suggesting some policy implications towards sustainable development goals (SDGs), specifically target 2.4.

In terms of applied methods, there are also differences from previous studies, for example, (5) applied the ‘Driscoll-Kraay for empirical analysis and the Method of Moments Quantile Regression is used to check robustness. In our study, modern econometric methods were applied, namely, the fully modified least-squares (FMOLS) and dynamic least-squares (DOLS) methods. Because they are capable of solving the endogeneity problem and serial correlation errors in time series models, they help to estimate the long-run relationship reliably. In addition, the authors used The Driscoll-Kraay (DSK) estimator and the generalized method of moments (GMM) to check the robustness. The DSK estimate is appropriate for the empirical analysis in the case of large cross-sections (29) and small time periods (22), as in this study, and solves the problems of heteroscedasticity, autocorrelation, and the cross-sectional dependence (CSD) in empirical models (33). Besides, in order to address the endogeneity problem in the model, the authors used GMM estimation to check the robustness. In addition, our study also test the causal relationship between variables using Pairwise Dumitrescu Hurlin Panel Causality Tests.

🡪Thank you for your comment, the authors have revised the theoretical framework, The authors have revised the dependent variable to be “agricultural productivity”. It is highlighted in red in the section of theoretical framework.

**3.2 Theoretical framework and study hypothesis**

The factors influencing agricultural production are presented based on the production function, where productivity is expressed in terms of functional inputs. Theoretically, agricultural productivity depends on capital (K) and labor (L). However, according to (36), the production function can be expanded using more input variables. The nexus between inputs and outputs in agricultural production can be presented based on the Cobb–Douglas production function was developed by Echevarria (36) as follows:

Y_it_ = θK_it_^α^ L_it_^β^ E_it_^є^	(1).

Where Y is output, is measured by AGP, K is capital, and L is labor, and E is other input. This study examined the effect of REC, ICT, NR, FD and IQ on AGP. When we calculate the natural logarithm of equation (1) and incorporate REC, ICT, NR, FD and IQ, following the studies (33); (34) as mentioned in Equation (2).

${lnAGP}_{i,t}=\alpha_{i,t}+{lnREC}_{i,t}+{lnICT}_{i,t}+{lnNR}_{i,t}{+{lnFD}_{i,t}+{IQ}_{i,t}+\varepsilon}_{i,t}$ (2)

Furthermore, Equation (2) is widened by introducing the interaction of (LnREC * LnICT) to examine the moderating influence of REC with ICT on AGP , following the studies (33), (5) as mentioned in Equation (3).

${lnAGP}_{i,t}=\alpha_{i,t}+{lnREC}_{i,t}+{lnICT}_{i,t}+lnREC*lnICT+{lnNR}_{i,t}{+{lnFD}_{i,t}+{IQ}_{i,t}+\varepsilon}_{i,t}$ (3)

*(6) Recommendation: If the authors have data with them, it is recommended to clearly define the variables, empirically justify the relationship between the variables, and reframe theoretical links between variable and re run the analysis.*

🡪Thank you for your comment, the authors have revised the literature review and theoretical framework showing the relationship between variables.

**Reviewer 2:**

*(1) Please apply the comments provided in the file and provide valid references for the formulas you have used in the text of the article.*

🡪Thank you for your comment, the authors have edited as per your suggestion.

*(2) Some comments are:*

*Edit:*

*Added value…*

*Use numerical information to express results.*

🡪Thank you for your comment, the authors have edited as per your suggestion.

*(3) Edit:*

*The climate….*

*Edit according to the in-text referencing format.*

*Replace with:*

*To make agriculture more sustainable, it is essential to ensure food security, enhance the value of agricultural products, and decrease carbon emissions from the agricultural sector.*

🡪Thank you for your comment, the authors have edited as per your suggestion.

In Asia, agriculture plays a vital role in the livelihoods of its people. Over 60% of the population, approximately 2.2 billion individuals, rely on agriculture for their income. However, the agricultural sector's contribution to the region's gross domestic product (GDP) has been declining, falling from an average of 11.1% in 2000 to 7.1% in 2023 (1). Climate change is threatening agricultural production, and thus, the livelihoods and food security of billions of people who depend on agriculture in the Asian region. Agriculture is highly vulnerable to climate change because of its dependence on the climate and weather. According to the Food and Agriculture Organization (FAO), Asia's total emissions from agricultural activities in 2018 reached 3.3 GtCO2e, which was nearly one-third of the global share from agriculture. China and India were significant emitters from agricultural production in 2018, with each country contributing around 650 million tonnes (Mt) of CO2eq annually (2). To make agriculture more sustainable, it is essential to ensure food security, enhance the value of agricultural products, and decrease carbon emissions from the agricultural sector.

*(4) Edit:*

*Added value…*

*Use numerical information to express results.*

🡪Thank you for your comment, the authors have edited as per your suggestion.

*(5) Edit:*

*The climate….*

*Edit according to the in-text referencing format.*

*Please provide a valid reference for this section.*

🡪Thank you for your comment, the authors have edited as per your suggestion.

*(6) Replace with:*

*In Asia, agriculture plays a vital role in the livelihoods of its people. Over 60% of the population, approximately 2.2 billion individuals, rely on agriculture for their income. However, the agricultural sector's contribution to the region's gross domestic product (GDP) has been declining, falling from an average of 11.1% in 2000 to 7.1% in 2023.*

*And edit the refrence formatting.*

🡪Thanks you for your comment, the authors have revised the information as per your suggestion.

In Asia, agriculture plays a vital role in the livelihoods of its people. Over 60% of the population, approximately 2.2 billion individuals, rely on agriculture for their income. However, the agricultural sector's contribution to the region's gross domestic product (GDP) has been declining, falling from an average of 11.1% in 2000 to 7.1% in 2023 (1).

**Reviewer 3:**

1. ***Title***

- *Consider a smoother phrasing to improve readability.*
- *“Asia nations” in the current version is grammatically awkward; use “Asian nations.”*

🡪 Thank you for your comment. The authors have edited the title according to your suggestion. “The relationship between renewable energy, information and communication technologies, financial development and agricultural productivity: Evidence from Asian nations”

2. ***Abstract:***

- *Revise grammar and clarity: e.g., “agricultural add valued (AVA)” → “agricultural value added (AVA).”*

🡪 Thank you for your comment. The authors have revised the dependent variable to be “agricultural productivity” as suggested by Reviewer 1, and have revised the abstract as suggested by you.

- *The phrase “illustrated a bidirectional causality among FD, and IQ with AVA” could be streamlined: “illustrated bidirectional causality between FD, IQ, and AVA.”*

*🡪* Thank you for your comment. The authors have revised according to your suggestion: “illustrated bidirectional causality between FD, IQ, and AVA.”

- *Suggest adding a novelty statement (e.g., how this study differs from existing literature in Asia).*

🡪 Thank you for your comment. The authors have edited and added the statement, which is highlighted in red in the abstract.

This study investigates the effect of renewable energy consumption (REC), information and communication technologies (ICT), natural resources (NR), financial development (FD) and institutional quality (IQ) on agricultural productivity (AGP) in 29 Asian countries for the 2000 – 2021 period. The fully modified least-squares (FMOLS) and dynamic least-squares (DOLS) estimation are used for the empirical analysis. The Driscoll and Kraay estimation **(**DSK) and the generalised method of moments (GMM) are used for robustness checks. The empirical results indicate that REC, ICT, FD, IQ have a significant positive impact on AGP. Specifically, a unit increase in REC, ICT, FD and IQ lead to a 6.097%, 2.292%, 0.688% and 0.002% points increase in AGP, respectively. Meanwhile, NR has a significant negative influence on AGP. Specifically, a unit increase in NR leads to 0.226% points reduce in AGP, implying that the resource curse persists in Asian countries. The results of the causality test illustrated bidirectional causality between FD, IQ, and AGP. Compared with previous studies in the Asian region, this study provides additional empirical evidence on the impact of REC and ICT on AGP, and the interaction of these two factors on agricultural development in the region. The research results suggest that managers need to issue policies to enhance sustainable AGP through the development of renewable energy sources, the application of ICT in agricultural production, and capital incentives for green projects to promote sustainable agricultural growth for Asian countries.

3. ***Introduction:***

- *Improve flow by condensing overly long paragraphs.*
- *Recheck factual clarity: the sentence “Countries are committed to halving emissions by 2023” seems inconsistent with international agreements (likely 2030).*
- *Some citations are heavily descriptive—consider synthesizing instead of listing.*
- *Highlight more explicitly what is new in this study compared to prior ASEAN, BRICS, or MENA analyses.*
- *Tighten narrative to emphasize research gap and contribution earlier. Replace raw URLs with proper citation formatting.*

*🡪* Thank you for your comment. The authors have edited the introduction according to your suggestion, it is highlighted in red.

**1. Introduction**

In Asia, agriculture plays a vital role in the livelihoods of its people. Over 60% of the population, approximately 2.2 billion individuals, rely on agriculture for their income. However, the agricultural sector's contribution to the region's gross domestic product (GDP) has been declining, falling from an average of 11.1% in 2000 to 7.1% in 2023 (1). Climate change is threatening agricultural production, and thus, the livelihoods and food security of billions of people who depend on agriculture in the Asian region. Agriculture is highly vulnerable to climate change because of its dependence on the climate and weather. According to the Food and Agriculture Organization (FAO), Asia's total emissions from agricultural activities in 2018 reached 3.3 GtCO2e, which was nearly one-third of the global share from agriculture. China and India were significant emitters from agricultural production in 2018, with each country contributing around 650 million tonnes (Mt) of CO2eq annually (2). To make agriculture more sustainable, it is essential to ensure food security, enhance the value of agricultural products, and decrease carbon emissions from the agricultural sector.

Burning fossil fuels releases large amounts of carbon dioxide (CO2) into the atmosphere, which is the main cause of the greenhouse effect and rising global temperatures. In 2019, the agricultural sector accounted for approximately 2 percent of total energy consumption in Southeast Asian countries. Energy consumption in this sector increased by 35 percent between 2009 and 2019 (3). This causes serious environmental degradation by releasing greenhouse gases that cause global warming, acid rain (from sulfur dioxide and nitrogen oxide), and air, water, and soil pollution. Countries are committed to achieving sustainable development goals, by halving emissions by 2030, and moving towards zero emissions by 2050. Therefore, innovation in agricultural production activities is urgent to realize the goal of reducing emissions, increasing agricultural productivity, ensuring food security and income for current and future generations, towards the goal of sustainable development. According to (4) renewable energy helps increase agricultural productivity (AGP) by providing a clean, stable source of energy for activities such as irrigation, drying agricultural products, lighting, and operating modern equipment, thereby reducing production costs, improving efficiency and promoting sustainable agricultural development. Therefore, considering the impact of renewable energy consumption (REC) on agricultural development in the Asian region is urgent in the current context. Renewable energy enhances sustainable AGP by reducing operating costs, providing clean, reliable energy for agricultural activities, and minimizing environmental impacts. Sources such as solar, wind, and biomass power help run machinery, pump water, treat waste, and reduce dependence on fossil fuels, contributing to climate change response. Furthermore, information and communication technology (ICT) plays a revolutionary role in the agricultural sector, helping to improve productivity and product quality, optimize resource use, save costs, expand market access channels and support decision-making, thanks to applications such as the Internet of Things (IoT), artificial intelligence (AI), and data analysis, towards a smart and sustainable agriculture. According to (5) ICT increases AGP because it helps optimize production processes and increase resource efficiency. Renewable energy provides clean, economical electricity for agricultural activities, while ICT provides monitoring and automatic control solutions, optimizes resource use and enhances adaptability to climate change, contributing to building a modern, efficient and sustainable agricultural system. Integrating ICT into agricultural production might allow farmers to better adapt to climate change through more modern production models. Moreover, the application of technology helps optimize resource use, increase productivity and reduce greenhouse gas emissions in agricultural production, while financial and institutional development plays an important role in investing, coordinating and creating a favorable legal corridor for the application of these technologies, thereby improving agricultural productivity in a sustainable manner (6). This study examines the relationship between REC, natural resources, ICT, financial development and agriculture productivity in Asian countries. From the research results, the authors will suggest some policy implications towards the goal of sustainable development in the region.

This study makes several contributions to the existing literature. Although previous studies have assessed the relationship between AGP and its determinants for various regions. For example, (5) examined the effect of renewable energy (RE), ICT and agricultural credit (AC) for sustainable AGP in developing nations. In case of the 10 Asia`s agricultural economies, (7) explored the effect of technological innovation, REC, financial development, globalization, and institutional quality on sustainable agriculture. (8) concentrated on the nexus among renewable and non-renewable energy agrotechnology, and agricultural productivity (AP) in 32 developed countries. (9) focused on examining the impacts of climate change (CC) and financial development on agricultural production in four ASEAN countries. Similarly, (10) concentrated on evaluating the effect of REC and NR on agricultural productivity in ASEAN countries. In case of BRICS countries, (11) focused on examining the link between AGP and REC with the role of financial inclusion and foreign direct investment (FDI). In case of the Middle East and North African (MENA) countries, (12) concentrated on the nexus between CC, renewable and non-renewable energy consumption and agricultural development. There is still a lack of research on the case of Asian countries. Specifically, studies have not yet emphasized the decisive role of REC and ICT on AGP in Asia. Thus, this study will explore how ICT facilitates the use of mobile technology to share knowledge and access weather information, aiding farmers in making informed decisions to enhance productivity and promote sustainable agriculture (13). Furthermore, renewable energy plays an important role in enhancing sustainable AGP. Because it helps reduce emissions from agricultural production activities, allowing agricultural products to be more environmentally friendly, towards sustainable agriculture (4). The interaction between REC and ICT can have a positive impact on sustainable AGP as they can help reduce environmental impacts, increase production efficiency and improve climate change adaptation. ICT plays a supporting role in the effective deployment and management of renewable energy, such as energy monitoring systems, while renewable energy provides clean electricity to operate smart agricultural technologies, creating a more modern and sustainable agricultural ecosystem. However, there is currently a lack of research examining this interaction on agricultural production in Asian regions. Through this study, we will provide more empirical evidence on this interaction. From there, the authors will suggest some policy implications to enhance AGP in the region. Moreover, financial resources are considered to be extremely important for farmers to invest in agricultural production activities, they allow farmers to invest in technological knowledge related to ICT and renewable energy, which is beneficial for the goal of sustainable agriculture. As the increasing consumption of fossil fuels and overexploitation of natural resources pose a threat to the environment, many studies have investigated the role of natural resources in promoting economic growth. However, few studies have examined the role of this factor in the agricultural sector. This study will provide further evidence on the direct impact of natural resources on AGP, thereby suggesting some policy implications towards sustainable development goals (SDGs), specifically target 2.4.

The authors use recent econometric methods to analyze the model. First, we tested the cross-sectional dependence (CSD). Because if the research skips this step, the results may be distorted (14). The CSD exists in the panel data, the athors applied the second-generation root tests for the analysis, and the panel cointegration tests are also applied. The fully modified least-squares (FMOLS) and dynamic least-squares (DOLS) methods were used for empirical analysis. The Driscoll-Kraay standard error (DK)’ approach and the generalised method of moments (GMM)-system estimator were used to check robustness.

The structure of this study is as follows: Section 2 provides a brief overview of the literature; Section 3 presents the methodology and data; Section 4 illustrates the outcomes and discussions; and Section 5 concludes the paper.

4. ***Literature review:***

- *Well-structured by themes (renewable energy, natural resources, ICT, financial development). Good coverage of recent and regional studies.*
- *Some redundancy (several studies described with similar outcomes). The section is long and reads like a summary rather than a critical synthesis.*
- *Condense by merging similar findings. Add a clearer positioning of how this study fills the identified gap.*

🡪 Thank you for your comment. The authors have edited and supplemented the literature review according to your suggestions.

**2. Literature review**

**2.1 Renewable energy and** **the agricultural productivity**

The world is facing a major challenge of climate change, partly caused by carbon emissions. To counter this risk, renewable energy should be considered for its contribution to Sustainable Development Goals (SDGs) for global countries. Several studies have examined the role of REC in enhancing sustainable agricultural productivity. For instance, (4) examined the link between REC and AGP in 123 nations during the period 1995-2019. They show that REC, foreign direct investment (FDI), and financial inclusion has a positive influence on AGP, whereas carbon dioxide emission (CO_2_E) show the opposite. Research suggests that farmers should limit the consumption of non-renewable energy sources for agricultural production and switch to enewable energy. Additionally, (11) investigated the link between AGP and REC in BRICS from 2000 to 2020. The outcomes indicate that REC, CO2E, and FDI positively affect AGP, whereas access to financial inclusion has no impact on AGP. (9) assessed the influence of climate change and FD on agricultural production in ASEAN-4 from 1990-2016. They found that climate change hurt gricultural production, whereas renewable energy, human capital (HC), and institutional quality (IQ) positively affected AGP. (10) evaluated the impact of REC and NR on AGP in ASEAN countries. They found that REC positively affected the AGP, whereas the forest area, CO2E, and NR negatively affected AGP. Similarly, (12) found that REC increases AGP in Middle East and North African nations. This study suggests that governments should target the development of renewable energy sources, particularly in the agricultural sector. REC boosts agricultural productivity was also found in the study of (15); (16); (8); (5) and (7).

Generally, some studies examined the nexus between REC and sustainable agricultural development, the outcomes of this influence in Asia still need to be clarified. RE can power irrigation systems, agricultural drying, helping farmers use water and land resources more efficiently. This study will provide further empirical evidence on the impact of REC on AGP in Asian countries. Futhermore, clean energy helps reduce agriculture's carbon footprint, while ICT provides digital solutions to optimize agricultural production and management. However, few studies have examined the interactive effects of REC and ICT on AGP. Considering this interaction is necessary because REC and ICT can promote sustainable AGP by reducing pollution, increasing resource efficiency and creating smarter production systems, thereby improving competitiveness and ensuring food security. RE provides clean power for smart agricultural equipment, such as using solar energy to operate soil sensors and automatic irrigation systems. Thus, the authors also examine the interactive effects of REC and ICT on AGP in Asian countries, thereby suggesting policy implications to promote sustainable agriculture in this region.

**2.2 Natural resources and the agricultural productivity**

The demand for fossil energy is increasing to serve economic growth, which leads to the overuse of natural resources and also causes large emissions. Recently, natural resources and environmental pollution have attracted much attention from researchers. Much of the literature focuses on the impact of natural resources on economic growth, emissions, and poverty. Few studies have examined the impact of natural resources on the agricultural sector. (10) show that CO_2_E, forest area, and NR decrease AGP. Similarly, (17) explored that resource windfalls reduce AGP in Sub-Saharan Africa, confirming the resource curse of agriculture in these countries. In the case of 25 oil-exporting nations, (18) studied the impact of oil prices on agriculture and showed that oil prices harmed on AGP. Implying that Dutch disease exists in these countries. In another research, (19) examined the impact of oil rents on AGP in the Middle East and North Africa (MENA) nations. They discovered a negative link between oil rents and AGP, showing that the oil industry boom is associated with a contraction in agriculture. Likewise, (20) discovered a negative nexus between NR abundance and AGP in Asia-Pacific Economic Cooperation (APEC) countries, implying that natural resource decreases AGP. This confirms the existence of a resource curse in these countries, due to an over-concentration in the resource sector, leading to the neglect of other sectors, increasing dependence on commodity price fluctuations. Poor governance, corruption and political instability also exacerbate this situation, preventing resources from being used effectively to promote sustainable growth.

In summary, many studies have examined the role of NR in economic growth, but few studies have examined the impact of NR on agriculture sector, particularly in Asia. Some studies have found that NR has a negative impact on AGP (10); (20). The authors will provide further empirical evidence on this relationship in Asian countries, thereby suggesting some policy implications for promoting sustainable AGP.

**2.3 Information and communication technologies (ICT) and the agricultural productivity**

The effective use of ICT to improve access to appropriate agricultural information and services holds great promise for transforming farmers' productivity and livelihoods. (21) examined the effect of ICT on agriculture sector performances (ASP) in sub-Saharan Africa (SSA) for the period of 1995–2017 by employing panel autoregressive distributed lag (ARDL) approach. The results show that ICT has a positive impact on ASP in the long run. However, there is no evidence that this effect persists in the short term. Similarly, (5) examined the effect of RE, ICT and agricultural credit (AC) for the sustainable AGP in developing nations for the 2000-2021 period by employing the Driscoll-Kraay and Quantile Regression to check robustness. They indicate that ICT have a positive impact on AGP. The moderating effect of the interaction between ICT and renewable energy is negative and weak. (13) investigated the mobile phones to support agricultural business management in Indonesia. The results show that mobile phone usage allows farmers to enhance sales, get market information, and acces to advanced agricultural technology. This helps increase profits for farmers. The study suggests encouraging farmers to use mobile phones to access information for agricultural business activities. According to (22), mobile networks are increasingly popular, making them easily accessible to users, which makes it easier for farmers to collect and share agricultural knowledge with each other. Similarly, according to (23), mobile phones enable farmers to easily exchange market information, receive weather updates, obtain agricultural extension advice, and learn about new farming technologies, thereby enhancing agricultural productivity. Similar results were also found in the study of (24); (25).

In short, some studies have investigated the impact of ICT on AGP, but this nexus still needs to be clarified in the Asian region. In addition, this study also examines the interaction effect of REC and ICT on AGP. Thereby, the authors will suggest some policy implications for the goal of sustainable AGP in the region for the coming time.

**2.4 Financial development (FD) and the agricultural productivity**

Investing financial resources for agricultural development is necessary, it allows farmers to convert to a more modern agricultural production model by applying modern technology, which helps the agricultural sector increase productivity and product quality. The important role of financial development in economic development has been proven by many studies, its role in the agricultural sector also needs to be clarified. (26) indicated that commercial loans to the farming sector significantly affect production in the country. Accordingly, the study suggests that commercial banks should support low-interest loans to farmers. Additionally, (27) examined the effect of financial development on AGP in South Asia and found the inverted U-shaped result of FD on agricultural production. Similar results were also found in the study of (9) for 4 ASEAN nations. They suggest that governments must adopt flexible financial and agricultural policies so that farmers can benefit and AGP can be increased. On the contrary, according to (28) digital inclusive finance reduces households' agricultural output. However, (29) examined the influence of global climate change, financial development (FD), and technical progress on cereal production in Pakistan from 1977 to 2014. They found that CO2 emissions negatively affect cereal production, whereas FD and technical progress have a positive influence on cereal production. Research suggests that financial development is necessary for sustainable AGP. Similar results were also found in the study of (30); (31); (32), they showed that financial development enhances AGP.

In general, the important role of financial development in economic development has been proven by many studies, its role in the agricultural sector also needs to be clarified. Some studies show that financial development boosts AGP, however some studies claim that financial development reduces AGP (28). The authors will provide further empirical evidence on this impact, in the case of Asian countries. Thereby, propose some policy implications to promote sustainable AGP through financial instruments.

**2.5 Research gap**

Although previous studies have assessed the relationship between AGP and its determinants for various regions. There is still a lack of research on the case of Asia. This study provides further empirical evidence on the impact of REC, NR, ICT, FD and IQ on AGP in this region. Specifically, studies have not yet emphasized the decisive role of REC and ICT in AGP. Therefore, this study will examine the role of ICT in using Mobile to share knowledge, access weather-related information to help farmers make accurate decisions to improve productivity, promote sustainable AGP (13). Furthermore, REC plays an important role in enhancing sustainable AGP. Because it helps reduce emissions from agricultural production activities, allowing agricultural products to be more environmentally friendly, towards sustainable agriculture (4). The interaction between REC and ICT can have a positive impact on AGP, as they can help reduce environmental impacts, increase production efficiency and improve climate change adaptation. ICT plays a supporting role in the effective deployment and management of renewable energy, such as energy monitoring systems, while renewable energy provides clean electricity to operate smart agricultural technologies, creating a more modern and sustainable agricultural ecosystem. However, there is currently a lack of research examining this interaction on agricultural production in Asian regions. Through this study, we will provide more empirical evidence on this interaction. From there, the authors will suggest some policy implications to enhance AGP in the region. Moreover, financial resources are considered to be extremely important for farmers to invest in agricultural production activities, they allow farmers to invest in technological knowledge related to ICT and renewable energy, which is beneficial for the goal of sustainable agriculture. As the increasing consumption of fossil fuels and overexploitation of natural resources pose a threat to the environment, many studies have investigated the role of natural resources in promoting economic growth. However, few studies have examined the role of this factor in the agricultural sector. This study will provide further evidence on the direct impact of natural resources on AGP, thereby suggesting some policy implications towards sustainable development goals (SDGs), specifically target 2.4.

In terms of applied methods, there are also differences from previous studies, for example, (5) applied the ‘Driscoll-Kraay for empirical analysis and the Method of Moments Quantile Regression is used to check robustness. In our study, modern econometric methods were applied, namely, the fully modified least-squares (FMOLS) and dynamic least-squares (DOLS) methods. Because they are capable of solving the endogeneity problem and serial correlation errors in time series models, they help to estimate the long-run relationship reliably. In addition, the authors used The Driscoll-Kraay (DSK) estimator and the generalized method of moments (GMM) to check the robustness. The DSK estimate is appropriate for the empirical analysis in the case of large cross-sections (29) and small time periods (22), as in this study, and solves the problems of heteroscedasticity, autocorrelation, and the cross-sectional dependence (CSD) in empirical models (33). Besides, in order to address the endogeneity problem in the model, the authors used GMM estimation to check the robustness. In addition, our study also test the causal relationship between variables using Pairwise Dumitrescu Hurlin Panel Causality Tests.

5. ***Methodology and Data:***

- *Data sources (World Bank, IMF) are credible. Variables are clearly defined in a table. The theoretical framework uses Cobb–Douglas function, which is appropriate.*
- *Hypotheses are numerous but not clearly linked to each part of the model. Measurement of “institutional quality” with only “voice and accountability” may not fully capture governance dimensions.*

🡪Thank you for your comment. The authors have edited the hypothesis section, it is highlighted in red in the section: 3.2 Theoretical framework and study hypothesis.

Asian countries face great challenges from climate change, which affects agricultural production and threatens national food security. Creating AGP while reducing emissions is the path towards sustainable agricultural development and green growth. The agricultural sector in developing countries plays an important role in creating jobs and ensuring national food security. On the other hand, this sector is also responsible for climate change, Asia's total emissions from agricultural activities in 2018 reached 3.3 GtCO2e, which was nearly one-third of the global share from agriculture (2). Therefore, to achieve the goal of sustainable agricultural development, it is necessary to use environmentally friendly inputs. According to (4) stated that REC increases agricultural productivity and helps reduce CO2E emissions (37). Converting fossil energy consumption to renewable energy in agricultural production to create more environmentally friendly products, meeting export market demand with high quality, low emission agricultural product standards. Thus, in this research, we assess the role of REC in enhancing AGP in the Asia region.

**Hypothesis 1**. REC significantly increases AGP in Asian countries.

ICT promotes sustainable AGP by applying technologies such as IoT, AI, drones, robots, and communication systems to monitor, automate, optimize production, and disseminate knowledge, minimize risks and create a platform for developing new business models. These technologies help reduce resource waste (water, fertilizer), increase productivity, improve product quality, and manage the environment. According to (5), ICT helps improve agricultural productivity and research suggests that developing countries need to integrate ICT in agricultural production. Therefore, in this study, we examine the effect of ICT on AGP in the Asia.

**Hypothesis 2.** ICT significantly increases AGP in the Asia.

ICT optimizes processes, helping farmers make better data-driven decisions, while renewable energy provides clean power for agricultural activities such as irrigation, lighting, heating and waste treatment, thereby creating an efficient, resilient and environmentally friendly agricultural system. According to (38), connecting digital devices to renewable energy has the potential to improve electricity efficiency, reduce energy consumption in the agricultural sector, leading to reduced operating costs, thereby promoting AGP. However, (5) agrued that the moderation effects of ICT with REC on AVA is negative. The study suggests that developing countries need to have appropriate policies in integrating renewable energy and applying information technology to promote sustainable AGP. Thus, in this research, we examine the interaction effect of REC with ICT on AGP in the Asia.

**Hypothesis 3.** The interaction effect of REC with ICT increases AGP in Asian region.

The NR curse has become a concern for the region due to the rapid increase in resource exploitation, leading to negative economic, social and environmental impacts. This theory states that countries with abundant natural resources have more difficulty achieving sustainable economic development, due to over-dependence on resource exploitation, price volatility and corruption (39). Many studies have examined the role of NR in economic growth. Few studies have evaluated the role of this factor in the agricultural sector. According to (10), NR reduces AGP in the ASEAN nations. (17) indicated that resource windfalls decrease AGP in 38 sub-Saharan Africa countries, confirming the NR curse of agriculture that exists in these nations. However, (40) stated that well-managed natural resources will improve incomes and ensure food security for rural people. Thus, in this research, we assess the role of NR on AGP in the Asia.

**Hypothesis 4**. NR significantly increases AGP in the Asia.

Moreover, financial investment is essential for farmers to convert to more modern farming models, applying modern science and technology to improve productivity and product quality. A healthy financial system with preferential interest rates will create opportunities for farmers to access capital for investment in production in a modern, environmentally friendly direction. Some studies show that financial development boosts agricultural productivity (30); (31); (32). However, some studies claimed that financial development reduces agricultural output (28). Thus, the authors will provide further empirical evidence on this impact, in the case of Asian countries.

**Hypothesis 5**. NR significantly increases AGP in Asian nations.

Good institutional quality plays an important role in promoting sustainable AGP because good institutions create a stable legal framework, encourage investment, allocate resources effectively, resolve disputes and ensure farmers' rights, thereby helping to balance production, environmental protection and income generation. (41) show the positive impact of governance on AGP, implying that a nation with better institutional quality can create more the agricultural products. However, (42) proposed a contrasting perspective, confirmed that governance hurts the crop production index. Thus, we will provide further empirical evidence on this impact, in the case of Asian countries.

**Hypothesis 6**. IQ significantly increases AGP in Asian nations.

- *Justify variable choices (especially IQ proxy). Clarify novelty in methodological approach compared to prior studies.*

🡪 Thank you for your comment. The authors have added the reasons for choosing the variables, which are highlighted in red in the section: 3.1 Data and variables. The authors have added a novelty in methodological approach, which is highlighted in red in the section 2.5 Research gap.

**3.1 Data and variables**

This study utilizes a panel dataset comprising 29 Asian countries, with data spanning the period from 2000 to 2021. Data is collected from the World Bank and the International Monetary Fund (IMF). In this research, AGP is the dependent variable, and REC, ICT, NR, FD and IQ are explanatory variables. Table 1 provides details of all the variables and their sources.

In the context of increasing climate change, which threatens food security, it is necessary to examine the role of REC, ICT, FD, NR and IQ on AGP in the Asian region. The research results will provide empirical evidence, thereby suggesting policy suggestions to promote sustainable AGP in the region. Sustainable AGP can be driven by renewable energy through reduced dependence on fossil fuels, ICT through its ability to optimize processes and improve efficiency. Moreover, the application of technology helps optimize resource use, increase productivity and reduce greenhouse gas emissions in agricultural production, while financial and institutional development plays an important role in investing, coordinating and creating a favorable legal corridor for the application of these technologies, thereby improving agricultural productivity in a sustainable manner (6). According to (5) REC and ICT promote sustainable AGP. Because using energy from clean sources such as solar, wind, biomass helps reduce greenhouse gas emissions, limit environmental pollution and protect natural resources. Besides, ICT provides solutions such as smart irrigation systems, data-based farm management, helping farmers make more accurate decisions about planting, caring, and harvesting. In addition, with wide coverage, ease of use, multi-function and low cost, mobile devices, especially phones, can solve the biggest challenge of farmers in connecting to the market, promoting the commercialization of agricultural products, helping the agricultural industry develop more strongly and sustainably. However, the interactive impact between REC and ICT on agricultural production is negative (5). Therefore, our study also provides further empirical evidence on this interaction effect on AGP in the Asian region. Furthermore, financial development plays a vital role in enhancing AGP as it provides the necessary capital to invest in environmentally friendly farming methods, modern technology and capacity building for farmers, thereby ensuring long-term economic benefits, improving farmers' lives and protecting natural resources. (29) stated that FD positively affect AGP, they suggested that the Pakistan government should invest financial resources in sustainable agricultural development. However, (28) indicated that digital inclusive finance reduces households' agricultural output. Thus, it is necessary to consider the impact of financial development on AGP in Asian countries. From the research results, the authors will suggest policy implications to promote AGP in this region. Moreover, institutional quality, particularly voice and accountability mechanisms, plays an important role in promoting AGP by allowing stakeholders to have a voice in policymaking, holding institutions accountable for their actions, and ensuring that agriculture initiatives are designed and implemented effectively, reflect real needs, and aim for sustainable outcomes. However, (34) agrued that institutional quality negatively affect AGP, they suggest that governments need to enact stricter regulations towards sustainable AGP. Farmers’ voices help policymakers better understand the challenges and opportunities in agriculture production, thereby building more appropriate and effective policies for sustainable development. Therefore, this study will examine the impact of IQ, specifically voice and accountability, on AGP, thereby suggesting solutions towards sustainable agricultural growth in the Asian region.

🡪Thank you for your comment. The authors have added a novelty in methodological approach, which is highlighted in red in the section 2.5 Research gap.

**2.5 Research gap**

Although previous studies have assessed the relationship between AGP and its determinants for various regions. There is still a lack of research on the case of Asia. This study provides further empirical evidence on the impact of REC, NR, ICT, FD and IQ on AGP in this region. Specifically, studies have not yet emphasized the decisive role of REC and ICT in AGP. Therefore, this study will examine the role of ICT in using Mobile to share knowledge, access weather-related information to help farmers make accurate decisions to improve productivity, promote sustainable AGP (13). Furthermore, REC plays an important role in enhancing sustainable AGP. Because it helps reduce emissions from agricultural production activities, allowing agricultural products to be more environmentally friendly, towards sustainable agriculture (4). The interaction between REC and ICT can have a positive impact on AGP, as they can help reduce environmental impacts, increase production efficiency and improve climate change adaptation. ICT plays a supporting role in the effective deployment and management of renewable energy, such as energy monitoring systems, while renewable energy provides clean electricity to operate smart agricultural technologies, creating a more modern and sustainable agricultural ecosystem. However, there is currently a lack of research examining this interaction on agricultural production in Asian regions. Through this study, we will provide more empirical evidence on this interaction. From there, the authors will suggest some policy implications to enhance AGP in the region. Moreover, financial resources are considered to be extremely important for farmers to invest in agricultural production activities, they allow farmers to invest in technological knowledge related to ICT and renewable energy, which is beneficial for the goal of sustainable agriculture. As the increasing consumption of fossil fuels and overexploitation of natural resources pose a threat to the environment, many studies have investigated the role of natural resources in promoting economic growth. However, few studies have examined the role of this factor in the agricultural sector. This study will provide further evidence on the direct impact of natural resources on AGP, thereby suggesting some policy implications towards sustainable development goals (SDGs), specifically target 2.4.

In terms of applied methods, there are also differences from previous studies, for example, (5) applied the ‘Driscoll-Kraay for empirical analysis and the Method of Moments Quantile Regression is used to check robustness. In our study, modern econometric methods were applied, namely, the fully modified least-squares (FMOLS) and dynamic least-squares (DOLS) methods. Because they are capable of solving the endogeneity problem and serial correlation errors in time series models, they help to estimate the long-run relationship reliably. In addition, the authors used The Driscoll-Kraay (DSK) estimator and the generalized method of moments (GMM) to check the robustness. The DSK estimate is appropriate for the empirical analysis in the case of large cross-sections (29) and small time periods (22), as in this study, and solves the problems of heteroscedasticity, autocorrelation, and the cross-sectional dependence (CSD) in empirical models (33). Besides, in order to address the endogeneity problem in the model, the authors used GMM estimation to check the robustness. In addition, our study also test the causal relationship between variables using Pairwise Dumitrescu Hurlin Panel Causality Tests.

6. **Results and Discussions:**

- Results are presented systematically (descriptive stats, correlation, multicollinearity, stationarity, cointegration, FMOLS/DOLS, robustness with DSK and GMM, causality tests). Interpretations are well connected to literature.
- Some tables are very detailed but under-discussed (e.g., causality test results). The negative interaction of ICT and REC is noted but not deeply explained. Discussion sometimes repeats results instead of analyzing implications.
- Expand discussion on why ICT*REC interaction is negative in Asia (possible issues of technology adoption, infrastructure, cost?). Reduce redundant restatements of results.

🡪 Thank you for your comment. The authors have added an extended discussion of why ICT*REC interactions are negative in Asia, which is highlighted in red in the section 4.1 Results.

With FMOLS and DOLS estimation, in Model II of Table 8, the moderation effects of ICT with REC are observed to have a significantly negative effect on AGP. This aligns with the findings of (5), which indicate that the moderating effects of ICT with REC on AGP are negative. Therefore, our results show that the combination of REC and ICT has an ineffective impact on agricultural development, which can be explained by barriers such as the lack of comprehensive and synchronous support policies for the application of renewable energy and ICT in agriculture, especially financial mechanisms to encourage investment. In addition, the small and fragmented scale of production in many rural areas is a major barrier to the application of advanced technologies, requiring large investment costs. High-quality human resources for the operation and maintenance of smart agricultural systems are also a challenge, and the problem of data integration is complex, making it difficult for farmers to access and apply. In addition, many modern technological solutions are too complicated, expensive and not suitable for the level and actual needs of farmers, leading to difficulties in implementation and operation. To overcome this, it is necessary to invest in digital infrastructure, build appropriate technology solutions, enhance digital skills training for farmers, have financial policies to support loans and tax incentives, encourage cooperatives and technology enterprises to participate, and at the same time build a smart and sustainable agricultural ecosystem.

7. **Conclusion:**

- Clearly summarizes empirical results and confirms/rejects hypotheses. Policy implications are relevant (green energy, ICT integration, financial support).
- Conclusion partly overlaps with policy implications; readability suffers. Limitations are only briefly addressed at the end.
- Separate Conclusion () from Policy Implications (recommendations). Expand Limitations (e.g., only 29 Asian countries, exclusion of climatic variables, single proxy for governance).

🡪 Thank you for your comment. The authors have separated the conclusion from the policy implications, the authors have edited and added the policy implications and research limitations as suggested by you, it is highlighted in red.

**5. Conclusion**

**5.1. Summary of findings**

This study analyzes the effect of renewable energy, information and communication technologies, natural resources, financial development and institutional quality on AGP in 29 Asian countries for the period of 2000 - 2021. The empirical outcomes show that all variables are stationary at the first difference I(1), based on the results of the CADF tests**.** All variables are cointegrated in the long run by the Kao test. The FMOLS, DOLS, DSK and GMM estimation are used for the empirical analysis. The empirical results indicate that renewable energy is found to have a positive influence on AGP, which confirms hypothesis 1 of this research. Additionally, hypothesis 2 is also confirmed, the findings show that ICT enhances AGP. It implies that ICT enables farmers to capture timely information, access markets, manage supplies, thereby improving AGP. However, the finding rejected hypothesis 3, which assumes that the interaction between ICT and REC enhances AGP in Asian countries. The finding rejected hypothesis 4, which assumes that natural resources contribute to AGP. On the other hand, the research results confirm hypothesis 5, financial development promotes AGP. Similarly, the study`s finding validates the hypothesis 6, institutional quality enhances AGP.

**5.2. Policy implications**

Based on research results, the suggestions have been made to enhance AGP in Asian countries.

First, renewable energy is found to have a positive influence on AGP. Therefore, governments and farmers need to pay attention to using renewable energy sources for agricultural production activities such as applying solar power for water pumps, agricultural drying systems, lighting systems and agricultural machinery running on renewable energy. Specifically, installing solar panels on farms to provide electricity for water pumps, irrigation systems, barn cooling, lighting, and production machinery; Using solar energy to dry agricultural products helps preserve products better without fuel costs; Developing and using agricultural equipment and vehicles such as tractors and harvesters that operate on electricity from renewable energy to replace traditional internal combustion engines; In areas with wind potential, install small-scale wind turbines to provide electricity for the farm; Utilizing agricultural by-products and organic waste to create biomass energy, providing heat and electricity for farm operations.

Second, policy implications related to the application of ICT to enhance AGP, ICT supporting sustainable agriculture through the use of the Internet of Things (IoT) to monitor the environment and crops, artificial intelligence (AI) to analyze data and make decisions, digital technology to manage the supply chain and improve transparency. To promote this, it is necessary to invest in digital infrastructure, upgrade telecommunications and Internet infrastructure to ensure stable and high-speed connections for the implementation of digital agricultural solutions; Train farmers on new technologies, organize training and coaching programs so that farmers can access and effectively use new technologies; Develop policies to support and encourage public-private partnerships, encourage cooperation between government agencies, research institutes, technology enterprises and farmers to jointly develop appropriate solutions; At the same time, develop applications suitable for local conditions, prioritizing the development of digital technology solutions suitable for farming conditions and the characteristics of each region of each country.

Third, the resource curse is a phenomenon in which many resource-rich countries have lower economic growth, higher corruption, and poorer development outcomes than countries with fewer resources, including those in Asia. To overcome this curse and move towards sustainable agricultural development, governments need to develop strong, transparent, and effective resource management policies, including strengthening accountability, controlling corruption, and promoting community participation.

Fourth, some solutions related to institutional quality, to promote sustainable AGP in Asian countries, it is necessary to focus on improving the legal framework, strengthening institutional capacity, promoting stakeholder participation, reforming financial and market institutions, and innovating technology and knowledge. Specific solutions include developing policies to support small-scale farmers, investing in research and application of sustainable technology, establishing effective monitoring and evaluation mechanisms, and promoting international cooperation to share knowledge and experience.

Some policy suggestions regarding financial support, Asian countries need to have specific financial support policies, such as interest rate incentives, credit guarantees, and risk insurance funds for sustainable agricultural projects and farmers adopting green farming methods; Developing innovative financial products, banks and financial institutions need to diversify financial products, such as green bonds, green credit, sustainable agricultural investment funds, to meet the diverse needs of the industry; Promoting public-private partnerships, enhance the role of the private sector in investing in sustainable agriculture, through public-private partnerships, to mobilize additional capital and technical expertise; Applying financial technology (Fintech), utilizing digital technology to build financial platforms for agriculture, helping farmers easily access information, borrow capital, and conduct transactions transparently and effectively. Specifically, the banking system needs to have a policy to reduce interest rates for farmers. A policy to reduce loan interest rates to create opportunities for farmers to access capital. This allows farmers to switch to modern farming models, innovate production technology, and increase productivity. Policies to encourage cleaner, low-carbon production need to be considered, prioritizing capital for clean production models to enhance AGP and move towards sustainable agriculture. Moreover, supporting agricultural cooperatives with capital and infrastructure to implement the circular economy, raising awareness, and promoting circular economic development is necessary. Waste from agricultural production can produce clean energy, which contributes to increase agricultural productivity, while reducing environmental pollution.

**5.3. The limitations of study**

Although this study finds evidence of the effect of renewable energy, information and communication technologies, natural resources, financial development and institutional quality on AGP in 29 Asian countries, it still has a few limitations. Future research could extend the investigation of the effects of climatic conditions on AGP for all Asian countries. For example, consider the impact of rainfall and temperature on agricultural productivity in this region. In addition, in this study, the authors only considered Voice and Accountability as a proxy for institutional quality, further research could consider six indicators such as Control of Corruption, Political Stability, Regulatory Quality, Government Effectiveness, Rule of Law and Voice and Accountability, and use principal component analysis to determine the value of institutional quality variables. In addition, the role of human resources should also be considered in relation to AGP. Because the high quality human resources can help farmers update new knowledge and modern technology more easily, thereby helping to improve agricultural productivity. Therefore, future research can incorporate these variables in relation to AGP, adding causal analysis between variables, providing policy makers with more perspectives for the goal of green agricultural growth.

8. **References:**

- Extensive and recent (up to 2024). Includes regional studies relevant to Asia.
- Some formatting inconsistencies (missing page numbers, inconsistent DOI presentation). Raw URLs in the text should be replaced with formatted references.
- Revise references to ensure journal-required style (PLOS ONE prefers consistent DOI format).

🡪 Thank you for your comment. The authors extended recent studies, and revised the reference format as per your suggestion.

**References**

1.	Bank W. World development indicators: World Bank; 2025.

2.	FAO. Emissions due to agriculture. Global, regional and country trends 2000-2018. FAOSTAT Analytical Brief 2020(18).

3.	IRENA. Renewable energy for agriculture: Insights from Southeast Asia.: International Renewable Energy Agency, Abu Dhabi.; 2022.

4.	Wang Y, Ahmed M, Raza SA, Ahmed M. Threshold nonlinear relationship between renewable energy consumption and agriculture productivity: the role of foreign direct investment and financial inclusion. Environmental Science and Pollution Research. 2023;30(24):65900-15.

5.	Sethi L, Behera P, Behera B, Sethi N. Unravelling the role of renewable energy, information and communication technology and agricultural credit for sustainable agricultural productivity in developing countries. International Journal of Sustainable Development & World Ecology. 2024;31(7):989-1003.

6.	Morkūnas M, Wang Y, Wei J. Role of AI and IoT in advancing renewable energy use in agriculture. Energies. 2024;17(23):5984.

7.	Sharma GD, Shah MI, Chopra R, Rao A, Shahzad U. Impact of technological advancement and greener energy on sustainable agriculture in Asia: Evidence from selected Asian countries. Sustainable Development. 2025;33(1):221-37.

8.	Muhamad GM. The Technological Effects of Renewable and Non-Renewable Energy on Agricultural Productivity in Developed Countries. International Journal of Empirical Economics. 2024;03(03):2450008.

9.	Chandio AA, Shah MI, Sethi N, Mushtaq Z. Assessing the effect of climate change and financial development on agricultural production in ASEAN-4: the role of renewable energy, institutional quality, and human capital as moderators. Environmental Science and Pollution Research. 2022;29(9):13211-25.

10.	Chopra R, Magazzino C, Shah MI, Sharma GD, Rao A, Shahzad U. The role of renewable energy and natural resources for sustainable agriculture in ASEAN countries: Do carbon emissions and deforestation affect agriculture productivity? Resources Policy. 2022;76:102578.

11.	Soni S, Rl M. The dynamic nexus between agricultural productivity and renewable energy consumption in BRICS: the role of financial inclusion and foreign direct investment. International Journal of Energy Sector Management. 2024;ahead-of-print(ahead-of-print).

12.	Karimi Alavijeh N, Salehnia N. Climate change, renewable and non-renewable energy consumption and agricultural development in the Middle East and North African countries. Environment, Development and Sustainability. 2023.

13.	Mariyono J, Santoso SI, Waskito J, Utomo AAS. Usage of mobile phones to support management of agribusiness activities in Indonesia. Aslib Journal of Information Management. 2022;74(1):110-34.

14.	Halkos GE, Polemis ML. Does financial development affect environmental degradation? Evidence from the OECD countries. Business Strategy and the Environment. 2017;26(8):1162-80.

15.	Qamruzzaman M. Nexus between renewable energy, foreign direct investment, and agro-productivity: The mediating role of carbon emission. Renewable Energy. 2022;184:526-40.

16.	Nendissa DR, Iriany A, Sui JM, Khoiriyah N, Suphattanakul O, Wisetsri W. The role of renewable and nonrenewable energy on agricultural economics in Indonesia. International Journal of Energy Economics and Policy. 2022;12(3):352-60.

17.	Dorinet E, Jouvet P-A, Wolfersberger J. Is the agricultural sector cursed too? Evidence from Sub-Saharan Africa. World Development. 2021;140:105250.

18.	Abdlaziz RA, Ahmad MNN, Slesman L. Dutch disease effect of oil price on agriculture sector: evidence from panel cointegration of oil exporting countries. International Journal of Energy Economics and Policy. 2018;8(5):241-50.

19.	Apergis N, El-Montasser G, Sekyere E, Ajmi AN, Gupta R. Dutch disease effect of oil rents on agriculture value added in Middle East and North African (MENA) countries. Energy Economics. 2014;45:485-90.

20.	Zhou Z, Sharif A, Inglesi-Lotz R, Bashir MF. Analysing the interplay between energy transition, resource consumption, deforestation, and environmental factors on agricultural productivity: Insights from APEC countries. Journal of Cleaner Production. 2024;446:141408.

21.	Oyelami LO, Sofoluwe NA, Ajeigbe OM. ICT and agricultural sector performance: empirical evidence from sub-Saharan Africa. Future Business Journal. 2022;8(1):18.

22.	Baumüller H. Towards smart farming? Mobile technology trends and their potential for developing country agriculture. Handbook on ICT in developing countries: River Publishers; 2022. p. 191-210.

23.	Uy TC, Ha HD, Truyen NN, Chung NV, Phuoc DN, Nam LV, et al. Mobile phone use for farm-related activities by ethnic minority farmers during the Covid-19 pandemic in Quang Tri Province, Central Vietnam. Information Services and Use. 2023;43(1):27-37.

24.	Erlangga E, Machuku O, Jun Dahino C. A review article on the impact and challenges of mobile phone usage on agricultural production in Africa. Cogent Food & Agriculture. 2023;9(2):2273634.

25.	Rajkhowa P, Qaim M. Mobile phones, off‐farm employment and household income in rural India. Journal of Agricultural Economics. 2022;73(3):789-805.

26.	Anetor F, Ogbechie C, Kelikume I, Ikpesu F. Credit supply and agricultural production in Nigeria: a vector autoregressive (VAR) approach. Journal of Economics and Sustainable Development. 2016;7(2).

27.	Zakaria M, Jun W, Khan MF. Impact of financial development on agricultural productivity in South Asia. Agricultural Economics/zemědělská Ekonomika. 2019;65(5).

28.	Liu Y, Liu C, Zhou M. Does digital inclusive finance promote agricultural production for rural households in China? Research based on the Chinese family database (CFD). China Agricultural Economic Review. 2021;13(2):475-94.

29.	Chandio AA, Jiang Y, Akram W, Adeel S, Irfan M, Jan I. Addressing the effect of climate change in the framework of financial and technological development on cereal production in Pakistan. Journal of Cleaner Production. 2021;288:125637.

30.	Cao L, Wang G. Impact of digital finance on agricultural output: From the perspective of digital development of agriculture. Finance Research Letters. 2024;66:105698.

31.	Ozdemir D. Reconsidering agricultural credits and agricultural production nexus from a global perspective. Food and Energy Security. 2024;13(1):e504.

32.	Yadav IS, Goyari P. The effects of financial development on crop productivity: ARDL evidence from Indian agriculture. Journal of Financial Economic Policy. 2024.

33.	Tran Phu Do T, Trinh Hoang Hong H, Thi D. Green finance, natural resources, and environmental quality in Asia: the moderating role of institutional quality. International Journal of Sustainable Energy. 2025;44(1):2553082.

34.	Tran TDP, Van NN, Thi D. The impact of green finance, natural resources and institutional quality on sustainable agriculture: evidence from Asian countries. Cogent Food & Agriculture. 2025;11(1):2488112.

35.	Gelgo B, Gemechu A, Bedemo A. The effect of institutional quality on agricultural value added in East Africa. Heliyon. 2023;9(10).

36.	Echevarria C. A Three-Factor Agricultural Production Function: The Case of Canada. International Economic Journal. 1998;12(3):63-75.

37.	Thi DM-T, Do TT-P. The interrelationships between carbon dioxide emissions and innovation: international evidence. International Journal of Sustainable Energy. 2024;43(1):2352515.

38.	Karlilar S, Balcilar M, Emir F. Environmental sustainability in the OECD: The power of digitalization, green innovation, renewable energy and financial development. Telecommunications Policy. 2023;47(6):102568.

39.	Sovacool BK. The political economy of oil and gas in Southeast Asia: heading towards the natural resource curse? The Pacific Review. 2010;23(2):225-59.

40.	Bakala F, Asfaw M. The role of natural resources for sustaining food security in Ethiopia: a review. Food Science and Quality Management. 2020;97(2224-6088):5-13.

41.	Lio M, Liu M-C. Governance and agricultural productivity: A cross-national analysis. Food Policy. 2008;33(6):504-12.

42.	Otim J, Watundu S, Mutenyo J, Bagire V, Adaramola MS. Effects of carbon dioxide emissions on agricultural production indexes in East African community countries: Pooled mean group and fixed effect approaches. Energy Nexus. 2023;12:100247.

43.	Pesaran MH. A simple panel unit root test in the presence of cross‐section dependence. Journal of applied econometrics. 2007;22(2):265-312.

44.	Behera P, Behera B, Sethi N. Assessing the impact of fiscal decentralization, green finance and green technology innovation on renewable energy use in European Union countries: What is the moderating role of political risk? Renewable Energy. 2024;229:120715.

**Reviewer 4:**

*For the manuscript PONE-D-25-41598 entitled ‘’ The relationship between renewable energy, information and communication technologies, financial development and sustainable agriculture: Evidence from Asia nations which has been submitted to PLOS ONE journal .*

*Thank you for submitting this manuscript to the PLOS ONE journal. Overall, the work is informative, well-organized, and clearly structured, though some sections require minor improvements.*

*Comment on the use of abbreviations: once you introduce an abbreviation, please use only the abbreviation thereafter. There is no need to repeat the full term with the abbreviation in parentheses each time. This issue appears throughout your manuscript, so please correct it.*

🡪 Thank you for your comment. The author have revised according to your suggestions.

*2.2 Natural resources and agriculture sector*

*under this subtitle there is a sentence it says ‘’Zhou et al. (2024) discovered a negative nexus between NR abundance and agricultural productivity (AP) in Asia-Pacific Economic Cooperation (APEC) countries, implying that natural resource decreases AP’’. How this could be true?*

🡪 Thank you for your comment. The authors have added and edited as per your suggestion, it is highlighted in red in the section of 2.2 Natural resources and the agricultural productivity

Likewise, (20) discovered a negative nexus between NR abundance and AGP in Asia-Pacific Economic Cooperation (APEC) countries, implying that natural resource decreases AGP. This confirms the existence of a resource curse in these countries, due to an over-concentration in the resource sector, leading to the neglect of other sectors, increasing dependence on commodity price fluctuations. Poor governance, corruption and political instability also exacerbate this situation, preventing resources from being used effectively to promote sustainable growth.

*Methodology section*

*The Methodology section is written in the future tense, but since the work has already been completed, it should be presented in the past tense. Therefore, this section requires revision.*

🡪 Thank you for your comment. The author have revised according to your suggestions.

**In the section of introduction:**

The authors use recent econometric methods to analyze the model. First, we tested the cross-sectional dependence (CSD). Because if the research skips this step, the results may be distorted (14). The CSD exists in the panel data, the athors applied the second-generation root tests for the analysis, and the panel cointegration tests are also applied. The fully modified least-squares (FMOLS) and dynamic least-squares (DOLS) methods were used for empirical analysis. The Driscoll-Kraay standard error (DK)’ approach and the generalised method of moments (GMM)-system estimator were used to check robustness.

**In the section of 4.1 Results:**

After conducting the CSD and stationarity tests, the authors continue with the next step of testing the long-run association between the variables in the model using the Kao test. The test outcomes in Table 7 indicate that there exists a long-run nexus between the variables. The results confirm a long-term relationship between the research variables. Thereby, FMOLS and DOLS methods were applied. The Driscoll-Kraay standard error (DK)’ approach and the GMM-system estimator were used to check robustness.
