# Supplementary material for: Renewable energy, information and communication technology, financial development, natural resources, institutional quality, and agricultural productivity in Asian countries
Source: PLoS One. 2026 Aug 14;21(8):e0355560. doi: 10.1371/journal.pone.0355560 (PMC13475886; doi:10.1371/journal.pone.0355560)
Supplement: Supplementary file 5 [file pone.0355560.s005.docx]

Dear Editor,

I am writing to provide my review of the manuscript titled “The relationship between renewable energy, information and communication technologies, financial development and agricultural productivity: Evidence from Asian nations “. I have carefully reviewed this revised manuscript for the second time. I commend the authors for their substantial improvements across all major sections. The narrative is now more coherent, the methodological explanations are clearer, and the empirical results are more convincingly discussed. The responses to earlier reviewer comments have been appropriately addressed, and the manuscript is now much closer to publication quality.

Before recommending acceptance, I suggest the authors consider the following two key revisions to further strengthen clarity:

1. Improve Conciseness and Reduce Repetition in the Introduction and Literature Review

Although improved, several paragraphs—particularly in the Introduction and Section 2—repeat similar arguments regarding the role of renewable energy, ICT, and financial development in supporting agricultural productivity. Streamlining these sections would improve readability and ensure a stronger narrative flow. The authors may consolidate overlapping explanations to highlight the novelty of this study more sharply.

2. Enhance the Policy Implications with More Focused and Actionable Recommendations

The Policy Implications section is rich but overly broad. To improve its usefulness for policymakers and readers, the authors should refine this section to present more specific, actionable, and evidence-driven recommendations that directly correspond to the empirical findings. Prioritizing key policies (e.g., renewable energy adoption in farming, ICT-capacity building, targeted financial instruments) will make the implications more impactful and aligned with the study’s contributions.
