# Supplementary material for: Renewable energy, information and communication technology, financial development, natural resources, institutional quality, and agricultural productivity in Asian countries
Source: PLoS One. 2026 Aug 14;21(8):e0355560. doi: 10.1371/journal.pone.0355560 (PMC13475886; doi:10.1371/journal.pone.0355560)
Supplement: Supplementary file 6 [file pone.0355560.s006.docx]

**PONE-D-25-41598R1**

**The relationship between renewable energy, information and communication technologies, financial development and agricultural productivity: Evidence from Asian nations**

**Journal: PLOS ONE**

**Dear Editor and the Reviewers,**

We would like to thank you for your comments and suggestions. Following your recommendations, we have made several revisions in response to the comments, to strengthen the arguments throughout the paper. Our detailed responses are shown below, with your comments in *italics and* bold, while our responses are given in the normal text.

**Reviewer: 1**

*(1) I am not satisfied with relating the variables and how each variable affects agriculture productivity. For instance the ICTs support can positively influence agriculture productivity through Accessing market information, Weather forecasts, digital agricultural extension advice, Early warning systems, Digital financial services. Similarly if we take Financial development as a factor to influence productivity, that is through improved access to credit, provision of affordable loans, use of digital banking, insurance and savings....etc.
In the manuscript, the variables were not well defined to indicate correlation between the dependent and independent.*

🡪Thank you for your comment, the authors have revised the manuscript according to your suggestions. The authors have revised the variable definitions according to your suggestion, which is highlighted in red in section 3.2 Theoretical framework and study hypothesis.

The increased consumption of renewable energy has a positive impact on agricultural productivity through reduced production costs, improved resource efficiency, and the promotion of sustainable development. Solar, wind, and biomass power systems provide a stable energy supply for irrigation, processing, and storage of agricultural products, thereby improving quality and yield. In addition, renewable energy contributes to reducing greenhouse gas emissions, improving soil and water environments, and creating favorable conditions for long-term agricultural production. [37] emphasized that renewable energy is a crucial factor in ensuring food and energy security, while also enhancing agricultural productivity through the integration of green technologies into production. Thus, in this research, we assess the role of REC in enhancing AGP in the Asia region.

Hypothesis 1. REC significantly increases AGP in Asian countries.

ICT is increasingly becoming a key factor in improving agricultural productivity. Through the provision of timely market information, accurate weather forecasts, and digital extension services, ICT helps farmers make more effective production decisions, minimize risks, and increase income. Furthermore, early warning systems and digital financial services support farmers in disease prevention, access to capital and crop insurance, contributing to sustainable production. [38] showed that the adoption of ICT significantly improved the technical efficiency of small-scale vegetable farmers in China. Similarly, [39] emphasized that ICT in extension services helps bridge the information gap and enhance agricultural productivity. According to [4], ICT helps improve agricultural productivity and research suggests that developing countries need to integrate ICT in agricultural production. Therefore, in this study, we examine the effect of ICT on AGP in the Asia.

Hypothesis 2. ICT significantly increases AGP in the Asia.

ICT optimizes processes, helping farmers make better data-driven decisions, while renewable energy provides clean power for agricultural activities such as irrigation, lighting, heating and waste treatment, thereby creating an efficient, resilient and environmentally friendly agricultural system. According to [40], connecting digital devices to renewable energy has the potential to improve electricity efficiency, reduce energy consumption in the agricultural sector, leading to reduced operating costs, thereby promoting AGP. However, [4] agrued that the moderation effects of ICT with REC on AVA is negative. The study suggests that developing countries need to have appropriate policies in integrating renewable energy and applying information technology to promote sustainable AGP. Thus, in this research, we examine the interaction effect of REC with ICT on AGP in the Asia. Based on the research results, the authors will provide some policy implications for improving agricultural productivity through the integration of ICT with REC in agricultural practices.

Hypothesis 3. The interaction effect of REC with ICT increases AGP in Asian region.

The NR curse has become a concern for the region due to the rapid increase in resource exploitation, leading to negative economic, social and environmental impacts. This theory states that countries with abundant natural resources have more difficulty achieving sustainable economic development, due to over-dependence on resource exploitation [41]. Many studies have examined the role of NR in economic growth. Few studies have evaluated the role of this factor in the agricultural sector. According to [10], NR reduces AGP in the ASEAN nations. [16] indicated that resource windfalls decrease AGP in 38 sub-Saharan Africa countries, confirming the NR curse of agriculture that exists in these nations. However, [42] stated that well-managed natural resources will improve incomes and ensure food security for rural people. In this research, we will add further empirical evidence on the relationship between NR and AGP in the Asian region.

Hypothesis 4. NR significantly increases AGP in the Asia.

Financial development (FD) is closely related to agricultural productivity, particularly through the expansion of credit, insurance, and modern payment services for farmers. As the financial system develops, farmers have easier access to capital to invest in machinery, new seeds, and production technologies, thereby improving efficiency and productivity. [43] confirmed that financial development has a positive impact on productivity and economic growth, with agriculture clearly benefiting. Furthermore, [44] demonstrates that agricultural credit from the formal financial system significantly impacts crop productivity, while [45] showed that limited access to credit reduces the production efficiency of smallholder farmers. This evidence reinforces the view that financial development is a crucial driver for improving agricultural productivity and promoting sustainable development. Moreover, some studies show that financial development boosts agricultural productivity [29, 30, 31]. However, some studies claimed that financial development reduces agricultural output [27]. Thus, the authors will provide further empirical evidence on this impact, in the case of Asian countries.

Hypothesis 5. FD significantly increases AGP in Asian nations.

Good institutional quality plays an important role in promoting sustainable AGP because good institutions create a stable legal framework, encourage investment, allocate resources effectively, resolve disputes and ensure farmers' rights, thereby helping to balance production, environmental protection and income generation. [46] show the positive impact of governance on AGP, implying that a nation with better institutional quality can create more the agricultural products. However, [47] proposed a contrasting perspective, confirmed that governance hurts the crop production index. Thus, we will provide further empirical evidence on this impact, in the case of Asian countries. The research findings will suggest several policy implications for improving agricultural productivity.

Hypothesis 6. IQ significantly increases AGP in Asian nations.

*(2) The points narrated as revised version over the earlier review comment is totally not needed. To describe renewable energy, the authors discussed about burning fossil fuels, carbon dioxide (CO2) releases greenhouse effect and rising global temperature; which are all less related to REC. What the authors should have explained was how renewable energy consumption contribute (directly or indirectly) to Agriculture productivity.*

*Anyways, the structure of the manuscript seems fine but its contribution to the body of knowledge remains limited.*

🡪Thank you for your comment, the authors have revised the manuscript according to your suggestions. The authors have removed unnecessary information as you suggested and have edited it, which is highlighted in red in the introduction.

Countries are committed to achieving sustainable development goals, by halving emissions by 2030, and moving towards zero emissions by 2050. Therefore, innovation in agricultural production activities is urgent to realize the goal of reducing emissions, increasing agricultural productivity, ensuring food security and income for current and future generations, towards the goal of sustainable development. According to [3], renewable energy helps increase agricultural productivity (AGP) by providing a clean, stable source of energy for activities such as irrigation, drying agricultural products, lighting, and operating modern equipment, thereby reducing production costs, improving efficiency and promoting sustainable agricultural development. Therefore, considering the impact of renewable energy consumption (REC) on agricultural development in the Asian region is urgent in the current context. According to [4], ICT increases AGP because it helps optimize production processes and increase resource efficiency. Renewable energy provides clean, economical electricity for agricultural activities, while ICT provides monitoring and automatic control solutions, optimizes resource use and enhances adaptability to climate change, contributing to building a modern, efficient and sustainable agricultural system. Integrating ICT into agricultural production might allow farmers to better adapt to climate change through more modern production models. Moreover, the application of technology helps optimize resource use, increase productivity and reduce greenhouse gas emissions in agricultural production, while financial and institutional development plays an important role in investing, coordinating and creating a favorable legal corridor for the application of these technologies, thereby improving agricultural productivity in a sustainable manner [5]. This study examines the relationship between REC, natural resources, ICT, financial development and agriculture productivity in Asian countries. From the research results, the authors will suggest some policy implications towards the goal of sustainable development in the region.

This study makes several contributions to the existing literature. Although previous studies have assessed the relationship between AGP and its determinants for various regions.. There is still a lack of research on the case of Asian countries. Specifically, studies have not yet emphasized the decisive role of REC and ICT on AGP in Asia. Thus, this study will explore how ICT facilitates the use of mobile technology to share knowledge and access weather information, aiding farmers in making informed decisions to enhance productivity and promote sustainable agriculture [6]. Furthermore, renewable energy plays an important role in enhancing sustainable AGP. Because it helps reduce emissions from agricultural production activities, allowing agricultural products to be more environmentally friendly, towards sustainable agriculture [3]. The interaction between REC and ICT can have a positive impact on sustainable AGP as they can help reduce environmental impacts, increase production efficiency and improve climate change adaptation. However, there is currently a lack of research examining this interaction on agricultural production in Asian regions. Through this study, we will provide more empirical evidence on this interaction. From there, the authors will suggest some policy implications to enhance AGP in the region. Moreover, financial resources are considered to be extremely important for farmers to invest in agricultural production activities, they allow farmers to invest in technological knowledge related to ICT and renewable energy, which is beneficial for the goal of sustainable agriculture. As the increasing consumption of fossil fuels and overexploitation of natural resources pose a threat to the environment, many studies have investigated the role of natural resources in promoting economic growth. However, few studies have examined the role of this factor in the agricultural sector. This study will provide further evidence on the direct impact of natural resources on AGP, thereby suggesting some policy implications towards sustainable development goals (SDGs), specifically target 2.4.

The authors use recent econometric methods to analyze the model. First, we tested the cross-sectional dependence (CSD). Because if the research skips this step, the results may be distorted [7]. The CSD exists in the panel data, the athors applied the second-generation root tests for the analysis, and the panel cointegration tests are also applied. The fully modified least-squares (FMOLS) and dynamic least-squares (DOLS) methods were used for empirical analysis. The Driscoll-Kraay standard error (DK)’ approach and the generalised method of moments (GMM)-system estimator were used to check robustness.

The structure of this study is as follows: Section 2 provides a brief overview of the literature; Section 3 presents the methodology and data; Section 4 illustrates the outcomes and discussions; and Section 5 concludes the paper.

**Reviewer: 3**

*I am writing to provide my review of the manuscript titled “The relationship between renewable energy, information and communication technologies, financial development and agricultural productivity: Evidence from Asian nations “. I have carefully reviewed this revised manuscript for the second time. I commend the authors for their substantial improvements across all major sections. The narrative is now more coherent, the methodological explanations are clearer, and the empirical results are more convincingly discussed. The responses to earlier reviewer comments have been appropriately addressed, and the manuscript is now much closer to publication quality.*

*Before recommending acceptance, I suggest the authors consider the following two key revisions to further strengthen clarity:*

*(1) Improve Conciseness and Reduce Repetition in the Introduction and Literature Review*

*Although improved, several paragraphs—particularly in the Introduction and Section 2—repeat similar arguments regarding the role of renewable energy, ICT, and financial development in supporting agricultural productivity. Streamlining these sections would improve readability and ensure a stronger narrative flow. The authors may consolidate overlapping explanations to highlight the novelty of this study more sharply.*

🡪Thank you for your comment, the authors have revised the manuscript according to your suggestions. The authors have revised the introduction and 2. Literature review, removing the duplicate sections as suggested by you; these are highlighted in red in the Introduction and Literature review.

1. Introduction

In Asia, agriculture plays a vital role in the livelihoods of its people. Over 60% of the population, approximately 2.2 billion individuals, rely on agriculture for their income. However, the agricultural sector's contribution to the region's gross domestic product (GDP) has been declining, falling from an average of 11.1% in 2000 to 7.1% in 2023 [1]. Climate change is threatening agricultural production, and thus, the livelihoods and food security of billions of people who depend on agriculture in the Asian region. Agriculture is highly vulnerable to climate change because of its dependence on the climate and weather. According to the Food and Agriculture Organization (FAO), Asia's total emissions from agricultural activities in 2018 reached 3.3 GtCO2e, which was nearly one-third of the global share from agriculture. China and India were significant emitters from agricultural production in 2018, with each country contributing around 650 million tonnes (Mt) of CO2eq annually [2]. To make agriculture more sustainable, it is essential to ensure food security, enhance the value of agricultural products, and decrease carbon emissions from the agricultural sector.

Countries are committed to achieving sustainable development goals, by halving emissions by 2030, and moving towards zero emissions by 2050. Therefore, innovation in agricultural production activities is urgent to realize the goal of reducing emissions, increasing agricultural productivity, ensuring food security and income for current and future generations, towards the goal of sustainable development. According to [3], renewable energy helps increase agricultural productivity (AGP) by providing a clean, stable source of energy for activities such as irrigation, drying agricultural products, lighting, and operating modern equipment, thereby reducing production costs, improving efficiency and promoting sustainable agricultural development. Therefore, considering the impact of renewable energy consumption (REC) on agricultural development in the Asian region is urgent in the current context. According to [4], ICT increases AGP because it helps optimize production processes and increase resource efficiency. Renewable energy provides clean, economical electricity for agricultural activities, while ICT provides monitoring and automatic control solutions, optimizes resource use and enhances adaptability to climate change, contributing to building a modern, efficient and sustainable agricultural system. Integrating ICT into agricultural production might allow farmers to better adapt to climate change through more modern production models. Moreover, the application of technology helps optimize resource use, increase productivity and reduce greenhouse gas emissions in agricultural production, while financial and institutional development plays an important role in investing, coordinating and creating a favorable legal corridor for the application of these technologies, thereby improving agricultural productivity in a sustainable manner [5]. This study examines the relationship between REC, natural resources, ICT, financial development and agriculture productivity in Asian countries. From the research results, the authors will suggest some policy implications towards the goal of sustainable development in the region.

This study makes several contributions to the existing literature. Although previous studies have assessed the relationship between AGP and its determinants for various regions.. There is still a lack of research on the case of Asian countries. Specifically, studies have not yet emphasized the decisive role of REC and ICT on AGP in Asia. Thus, this study will explore how ICT facilitates the use of mobile technology to share knowledge and access weather information, aiding farmers in making informed decisions to enhance productivity and promote sustainable agriculture [6]. Furthermore, renewable energy plays an important role in enhancing sustainable AGP. Because it helps reduce emissions from agricultural production activities, allowing agricultural products to be more environmentally friendly, towards sustainable agriculture [3]. The interaction between REC and ICT can have a positive impact on sustainable AGP as they can help reduce environmental impacts, increase production efficiency and improve climate change adaptation. However, there is currently a lack of research examining this interaction on agricultural production in Asian regions. Through this study, we will provide more empirical evidence on this interaction. From there, the authors will suggest some policy implications to enhance AGP in the region. Moreover, financial resources are considered to be extremely important for farmers to invest in agricultural production activities, they allow farmers to invest in technological knowledge related to ICT and renewable energy, which is beneficial for the goal of sustainable agriculture. As the increasing consumption of fossil fuels and overexploitation of natural resources pose a threat to the environment, many studies have investigated the role of natural resources in promoting economic growth. However, few studies have examined the role of this factor in the agricultural sector. This study will provide further evidence on the direct impact of natural resources on AGP, thereby suggesting some policy implications towards sustainable development goals (SDGs), specifically target 2.4.

The authors use recent econometric methods to analyze the model. First, we tested the cross-sectional dependence (CSD). Because if the research skips this step, the results may be distorted [7]. The CSD exists in the panel data, the athors applied the second-generation root tests for the analysis, and the panel cointegration tests are also applied. The fully modified least-squares (FMOLS) and dynamic least-squares (DOLS) methods were used for empirical analysis. The Driscoll-Kraay standard error (DK)’ approach and the generalised method of moments (GMM)-system estimator were used to check robustness.

The structure of this study is as follows: Section 2 provides a brief overview of the literature; Section 3 presents the methodology and data; Section 4 illustrates the outcomes and discussions; and Section 5 concludes the paper.

2. Literature review

2.1 Renewable energy and the agricultural productivity

The world is facing a major challenge of climate change, partly caused by carbon emissions. To counter this risk, renewable energy should be considered for its contribution to Sustainable Development Goals (SDGs) for global countries. Several studies have examined the role of REC in enhancing sustainable agricultural productivity. For instance, [3] examined the link between REC and AGP in 123 nations during the period 1995-2019. They show that REC, foreign direct investment (FDI), and financial inclusion has a positive influence on AGP, whereas carbon dioxide emission (CO_2_E) show the opposite. Research suggests that farmers should reduce the consumption of non-renewable energy sources for agricultural production and switch to renewable energy. Additionally, [8] investigated the link between AGP and REC in BRICS from 2000 to 2020. The outcomes indicate that REC, CO2E, and FDI positively affect AGP, whereas access to financial inclusion has no impact on AGP. [9] assessed the influence of climate change and FD on agricultural production in ASEAN-4 from 1990-2016. They found that climate change hurt gricultural production, whereas renewable energy, human capital (HC), and institutional quality (IQ) positively affected AGP. [10] evaluated the impact of REC and NR on AGP in ASEAN countries. They found that REC positively affected the AGP, whereas the forest area, CO2E, and NR negatively affected AGP. Similarly, [11] found that REC increases AGP in Middle East and North African nations. This study suggests that governments should target the development of renewable energy sources, particularly in the agricultural sector. REC boosts agricultural productivity was also found in the study of [12]; [13]; [14]; [4] and [15].

Generally, some studies examined the nexus between REC and sustainable agricultural development, the outcomes of this influence in Asia still need to be clarified. This study will provide further empirical evidence on the impact of REC on AGP in Asian countries, thereby suggesting policy implications to promote sustainable agriculture in this region.

2.2 Natural resources (NR) and the agricultural productivity

The demand for fossil energy is increasing to serve economic growth, which leads to the overuse of natural resources and also causes large emissions. Recently, NR and environmental pollution have attracted much attention from researchers. Much of the literature focuses on the impact of NR on economic growth, emissions, and poverty. Few studies have examined the impact of NR on the agricultural sector. [10] show that CO_2_E, forest area, and NR decrease AGP. Similarly, [16] explored that resource windfalls reduce AGP in Sub-Saharan Africa, confirming the resource curse of agriculture in these countries. In the case of 25 oil-exporting nations, [17] studied the impact of oil prices on agriculture and showed that oil prices harmed on AGP. Implying that Dutch disease exists in these countries. In another research, [18] examined the impact of oil rents on AGP in the Middle East and North Africa (MENA) nations. They discovered a negative link between oil rents and AGP, showing that the oil industry boom is associated with a contraction in agriculture. Likewise, [19] discovered a negative nexus between NR abundance and AGP in Asia-Pacific Economic Cooperation (APEC) countries, implying that natural resource decreases AGP. This confirms the existence of a resource curse in these countries, due to an over-concentration in the resource sector, leading to the neglect of other sectors, increasing dependence on commodity price fluctuations. Poor governance, corruption and political instability also exacerbate this situation, preventing resources from being used effectively to promote sustainable growth.

In summary, several studies have examined the role of NR in economic growth, but few studies have examined the impact of NR on agriculture sector, particularly in Asia. Some studies have found that NR has a negative impact on AGP [10,19]. The authors will provide further empirical evidence on this relationship in Asian countries, thereby suggesting some policy implications for promoting sustainable AGP.

2.3 Information and communication technologies (ICT) and the agricultural productivity

The effective use of ICT to improve access to appropriate agricultural information and services holds great promise for transforming farmers' productivity and livelihoods. [20] examined the effect of ICT on agriculture sector performances (ASP) in sub-Saharan Africa (SSA) for the period of 1995–2017 by employing panel autoregressive distributed lag (ARDL) approach. The results show that ICT has a positive impact on ASP in the long run. However, there is no evidence that this effect persists in the short term. Similarly, [4] examined the effect of RE, ICT and agricultural credit (AC) for the sustainable AGP in developing nations for the 2000-2021 period by employing the Driscoll-Kraay and Quantile Regression to check robustness. They indicate that ICT have a positive impact on AGP. The moderating effect of the interaction between ICT and renewable energy is negative and weak. [6] investigated the mobile phones to support agricultural business management in Indonesia. The results show that mobile phone usage allows farmers to enhance sales, get market information, and acces to advanced agricultural technology. This helps increase profits for farmers. The study suggests encouraging farmers to use mobile phones to access information for agricultural business activities. According to [21], mobile networks are increasingly popular, making them easily accessible to users, which makes it easier for farmers to collect and share agricultural knowledge with each other. Similarly, according to [22], mobile phones enable farmers to easily exchange market information, receive weather updates, obtain agricultural extension advice, and learn about new farming technologies, thereby enhancing agricultural productivity. Similar results were also found in the study of [23, 24].

In short, several studies have investigated the impact of ICT on AGP, but this nexus still needs to be clarified in the Asian region. In addition, this study also examines the interaction effect of REC and ICT on AGP. Thereby, the authors will suggest some policy implications for the goal of sustainable AGP in the region for the coming time.

2.4 Financial development (FD) and the agricultural productivity

Investing financial resources for agricultural development is necessary, it allows farmers to convert to a more modern agricultural production model by applying modern technology, which helps the agricultural sector increase productivity and product quality. The important role of financial development in economic development has been proven by many studies, its role in the agricultural sector also needs to be clarified. [25] indicated that commercial loans to the farming sector significantly affect production in the country. Accordingly, the study suggests that commercial banks should support low-interest loans to farmers. Additionally, [26] examined the effect of financial development on AGP in South Asia and found the inverted U-shaped result of FD on agricultural production. Similar results were also found in the study of [9] for 4 ASEAN nations. They suggest that governments must adopt flexible financial and agricultural policies so that farmers can benefit and AGP can be increased. On the contrary, according to [27] digital inclusive finance reduces households' agricultural output. However, [28] examined the influence of global climate change, financial development (FD), and technical progress on cereal production in Pakistan from 1977 to 2014. They found that CO2 emissions negatively affect cereal production, whereas FD and technical progress have a positive influence on cereal production. Research suggests that financial development is necessary for sustainable AGP. Similar results were also found in the studies of [29, 30, 31], they showed that financial development enhances AGP.

In general, the important role of financial development in economic development has been proven by many studies, its role in the agricultural sector also needs to be clarified. Some studies show that financial development boosts AGP, however some studies claim that financial development reduces AGP [27]. The authors will provide further empirical evidence on this impact, in the case of Asian countries. Thereby, propose some policy implications to promote sustainable AGP through financial instruments.

2.5 Research gap

Although previous studies have assessed the relationship between AGP and its determinants for various regions. There is still a lack of research on the case of Asia. This study provides further empirical evidence on the impact of REC, NR, ICT, FD and IQ on AGP in this region. Specifically, studies have not yet emphasized the decisive role of REC and ICT in AGP. Therefore, this study will examine the role of ICT in using Mobile to share knowledge, access weather-related information to help farmers make accurate decisions to improve productivity, promote sustainable AGP [6]. The interaction between REC and ICT can have a positive impact on AGP, as they can help reduce environmental impacts, increase production efficiency and improve climate change adaptation. ICT plays a supporting role in the effective deployment and management of renewable energy, such as energy monitoring systems, while renewable energy provides clean electricity to operate smart agricultural technologies, creating a more modern and sustainable agricultural ecosystem. However, there is currently a lack of research examining this interaction on agricultural production in Asian regions. Through this study, we will provide more empirical evidence on this interaction. From there, the authors will suggest some policy implications to enhance AGP in the region. Moreover, financial resources are considered to be extremely important for farmers to invest in agricultural production activities, they allow farmers to invest in technological knowledge related to ICT and renewable energy, which is beneficial for the goal of sustainable agriculture. As the increasing consumption of fossil fuels and overexploitation of natural resources pose a threat to the environment, many studies have investigated the role of natural resources in promoting economic growth. However, few studies have examined the role of this factor in the agricultural sector. This study will provide further evidence on the direct impact of natural resources on AGP, thereby suggesting some policy implications towards sustainable development goals (SDGs), specifically target 2.4.

In terms of applied methods, there are also differences from previous studies, for example, [4] applied the ‘Driscoll-Kraay for empirical analysis and the Method of Moments Quantile Regression is used to check robustness. In our study, modern econometric methods were applied, namely, the fully modified least-squares (FMOLS) and dynamic least-squares (DOLS) methods. Because they are capable of solving the endogeneity problem and serial correlation errors in time series models, they help to estimate the long-run relationship reliably. In addition, the authors used The Driscoll-Kraay (DSK) estimator and the generalized method of moments (GMM) to check the robustness. The DSK estimate is appropriate for the empirical analysis in the case of large cross-sections (29) and small time periods [22], as in this study, and solves the problems of heteroscedasticity, autocorrelation, and the cross-sectional dependence (CSD) in empirical models [32]. Besides, in order to address the endogeneity problem in the model, the authors used GMM estimation to check the robustness. Furthermore, our study also test the causal relationship between variables using Pairwise Dumitrescu Hurlin Panel Causality Tests.

*(2) Enhance the Policy Implications with More Focused and Actionable Recommendations*

*The Policy Implications section is rich but overly broad. To improve its usefulness for policymakers and readers, the authors should refine this section to present more specific, actionable, and evidence-driven recommendations that directly correspond to the empirical findings. Prioritizing key policies (e.g., renewable energy adoption in farming, ICT-capacity building, targeted financial instruments) will make the implications more impactful and aligned with the study’s contributions.*

🡪Thank you for your comment, the authors have revised the manuscript according to your suggestions. The authors have revised the policy implications section according to your suggestion, it is highlighted in red in section 5.2. Policy implications.

5.2. Policy implications

Based on research results, the suggestions have been made to enhance AGP in Asian countries.

First, renewable energy is found to have a positive influence on AGP. Therefore, governments should encourage the use of renewable energy sources for agricultural production. For example, in some countries, with the potential to produce clean energy from wind power, such as Vietnam, local authorities implement the construction of clean energy production from wind power, which is the process of using wind power as a renewable energy source, converting the kinetic energy of wind into electricity through wind turbines, without emitting greenhouse gases, contributing to reducing pollution and dependence on fossil fuels. Accordingly, this energy source can be used to dry agricultural products, pump water, and provide lighting, such as irrigation and lighting systems for dragon fruit plants. In addition, countries should encourage the production of biomass energy from agricultural by-products and organic waste, which provides heat and electricity for farm operations at farmers' households.

Second, policy implications related to the application of ICT to enhance AGP. Governments should encourage farmers and cooperatives to apply artificial intelligence to diagnose crop diseases and optimize fertilizer and pesticide use. Because AI can help analyze data from the environment and crops, make predictions, and support decision-making, it can help farmers improve productivity, reduce costs, and promote sustainable agricultural development. For example, information about impending heavy rainfall could help farmers postpone pesticide spraying or harvest crops in a timely manner to minimize losses. Additionally, consideration should be given to using drones for spraying and sowing to optimize agricultural practices. In addition, local authorities should implement policies to encourage farmers to use agricultural robots for weeding, crop care, and harvesting, which could lead to increased productivity. These devices can be operated remotely or automatically, offering convenience and high efficiency for users.

Third, some policy suggestions regarding financial support. Governments and financial institutions should consider providing preferential financial support and loan packages for farmers. Simultaneously, collaboration between technology companies and farmers is also an effective solution to alleviate the financial burden on farmers, as the initial investment costs for technologies applied to agricultural production are quite high. Specifically, the banking system needs to have a policy to reduce interest rates for farmers. This allows farmers to switch to modern farming models, innovate production technology, and increase productivity. Policies to encourage cleaner, low-carbon production need to be considered, prioritizing capital for clean production models to enhance AGP and move towards sustainable agriculture. Moreover, supporting agricultural cooperatives with capital and infrastructure to implement the circular economy. Waste from agricultural production can produce clean energy, which contributes to increase agricultural productivity, while reducing environmental pollution.

Fourth, this result shows that the resource curse exists in Asia. Therefore, to break the "resource curse," governments should implement policies that shift from exploitation-based growth to efficiency- and knowledge-based growth. For example, applying tax incentives and green credit mechanisms for businesses investing in deep processing, precision agriculture, and seed technology, in order to escape the trap of exporting cheap raw materials.

Furthermore, the policy implications relate to institutional quality. Governments should refine the legal framework to encourage investment in wind, solar, and biomass energy for agricultural production and processing. Furthermore, governments should create transparent grid connection mechanisms to allow farmers to sell surplus electricity. In addition, it is necessary to develop rural digital infrastructure, standardize data systems and traceability, and encourage the application of IoT, AI, and blockchain in production management, thereby enhancing the efficiency and transparency of the value chain. Financially, governments should expand rural credit, develop agricultural insurance and green financial products, and encourage the mobilization of private and international capital through green bonds and public-private partnerships. These institutional reforms will lay the foundation for a modern agricultural ecosystem, moving towards green and sustainable agriculture.

**Reviewer: 4**

*My comments have been taken care of; however, the conclusion section is still too long. It reads more like a summary than a conclusion.*

🡪Thank you for your comment, the authors have revised the manuscript according to your suggestions. The authors have revised the conclusion, which is highlighted in red in section 5. Conclusion

5.1. Summary of findings

This study analyzes the effect of renewable energy, information and communication technologies, natural resources, financial development and institutional quality on AGP in 29 Asian countries for the period of 2000 - 2021. The empirical outcomes show that all variables are stationary at the first difference I(1), based on the results of the CADF tests**.** All variables are cointegrated in the long run by the Kao test. The FMOLS, DOLS, DSK and GMM estimation are used for the empirical analysis. The empirical results indicate that renewable energy positively affects AGP, which confirms hypothesis 1. Additionally, hypothesis 2 is also confirmed, the findings show that ICT enhances AGP. However, the finding rejected hypothesis 3, which assumes that the interaction between ICT and REC enhances AGP in Asian countries. The finding rejected hypothesis 4, which assumes that natural resources contribute to AGP. In contrast, the results confirm hypothesis 5, financial development promotes AGP. Similarly, the study`s finding validates the hypothesis 6, institutional quality enhances AGP.

5.2. Policy implications

Based on research results, the suggestions have been made to enhance AGP in Asian countries.

First, renewable energy is found to have a positive influence on AGP. Therefore, governments should encourage the use of renewable energy sources for agricultural production. For example, in some countries, with the potential to produce clean energy from wind power, such as Vietnam, local authorities implement the construction of clean energy production from wind power, which is the process of using wind power as a renewable energy source, converting the kinetic energy of wind into electricity through wind turbines, without emitting greenhouse gases, contributing to reducing pollution and dependence on fossil fuels. Accordingly, this energy source can be used to dry agricultural products, pump water, and provide lighting, such as irrigation and lighting systems for dragon fruit plants. In addition, countries should encourage the production of biomass energy from agricultural by-products and organic waste, which provides heat and electricity for farm operations at farmers' households.

Second, policy implications related to the application of ICT to enhance AGP. Governments should encourage farmers and cooperatives to apply artificial intelligence to diagnose crop diseases and optimize fertilizer and pesticide use. Because AI can help analyze data from the environment and crops, make predictions, and support decision-making, it can help farmers improve productivity, reduce costs, and promote sustainable agricultural development. For example, information about impending heavy rainfall could help farmers postpone pesticide spraying or harvest crops in a timely manner to minimize losses. Additionally, consideration should be given to using drones for spraying and sowing to optimize agricultural practices. In addition, local authorities should implement policies to encourage farmers to use agricultural robots for weeding, crop care, and harvesting, which could lead to increased productivity. These devices can be operated remotely or automatically, offering convenience and high efficiency for users.

Third, some policy suggestions regarding financial support. Governments and financial institutions should consider providing preferential financial support and loan packages for farmers. Simultaneously, collaboration between technology companies and farmers is also an effective solution to alleviate the financial burden on farmers, as the initial investment costs for technologies applied to agricultural production are quite high. Specifically, the banking system needs to have a policy to reduce interest rates for farmers. This allows farmers to switch to modern farming models, innovate production technology, and increase productivity. Policies to encourage cleaner, low-carbon production need to be considered, prioritizing capital for clean production models to enhance AGP and move towards sustainable agriculture. Moreover, supporting agricultural cooperatives with capital and infrastructure to implement the circular economy. Waste from agricultural production can produce clean energy, which contributes to increase agricultural productivity, while reducing environmental pollution.

Fourth, this result shows that the resource curse exists in Asia. Therefore, to break the "resource curse," governments should implement policies that shift from exploitation-based growth to efficiency- and knowledge-based growth. For example, applying tax incentives and green credit mechanisms for businesses investing in deep processing, precision agriculture, and seed technology, in order to escape the trap of exporting cheap raw materials.

Furthermore, the policy implications relate to institutional quality. Governments should refine the legal framework to encourage investment in wind, solar, and biomass energy for agricultural production and processing. Furthermore, governments should create transparent grid connection mechanisms to allow farmers to sell surplus electricity. In addition, it is necessary to develop rural digital infrastructure, standardize data systems and traceability, and encourage the application of IoT, AI, and blockchain in production management, thereby enhancing the efficiency and transparency of the value chain. Financially, governments should expand rural credit, develop agricultural insurance and green financial products, and encourage the mobilization of private and international capital through green bonds and public-private partnerships. These institutional reforms will lay the foundation for a modern agricultural ecosystem, moving towards green and sustainable agriculture.
