# Supplementary material for: Renewable energy, information and communication technology, financial development, natural resources, institutional quality, and agricultural productivity in Asian countries
Source: PLoS One. 2026 Aug 14;21(8):e0355560. doi: 10.1371/journal.pone.0355560 (PMC13475886; doi:10.1371/journal.pone.0355560)
Supplement: Supplementary file 7 [file pone.0355560.s007.docx]

**PONE-D-25-41598R2**

**The relationship between renewable energy, information and communication technologies, financial development and agricultural productivity: Evidence from Asian nations**

**Journal: PLOS ONE**

**Dear Editor and the Reviewers,**

We would like to thank you for your comments and suggestions. Following your recommendations, we have made several revisions in response to the comments, to strengthen the arguments throughout the paper. Our detailed responses are shown below, with your comments in *italics and* bold, while our responses are given in the normal text.

**Reviewer: 1**

*The author used sophisticated statistics, but my concern has not been addressed through out. No need to repeat, but to remind, the title of the manuscript is about "The relationship between renewable energy, information and communication technologies, financial development and agricultural productivity".*

*REVIEW SESSION EXPLAINS about*

*1. Renewable energy*

*2. Natural resource*

*3. Information and communication technologies, as agricultural productivity determining factors:*

*From Research Gap (2.5) one can read as if the title of the research is;*

*1. The role of ICT in using Mobile phone for information exchange*

*2. It also looks as “role of ICT in the effective deployment and management of renewable energy”*

*FINDING SESSION talks about the effect ….*

*The effect of renewable energy, information and communication technologies, natural resources, financial development and institutional quality on AGP.*

*Generally, I couldn't see any relationship between the title and the main part of the paper including statistical results. My final recommendation is to align the title-review-objectives, results-findings-recommendation.*

🡪 Thank you for your comment, the authors have revised the title and content of the manuscript according to your suggestions; this is highlighted in red in the manuscript.
