# Supplementary material for: Renewable energy, information and communication technology, financial development, natural resources, institutional quality, and agricultural productivity in Asian countries
Source: PLoS One. 2026 Aug 14;21(8):e0355560. doi: 10.1371/journal.pone.0355560 (PMC13475886; doi:10.1371/journal.pone.0355560)
Supplement: Peer review history

# Peer review: Decision Letter 0

**DOI:** 10.1371/journal.pone.0355560.r001

**Authors:** Hesham M.H. Zakaly

11 Sep 2025

Dear Dr. Thi,

Thank you for submitting your manuscript to PLOS ONE. After careful consideration, we feel that it has merit but does not fully meet PLOS ONE’s publication criteria as it currently stands. Therefore, we invite you to submit a revised version of the manuscript that addresses the points raised during the review process.

plosone@plos.org. When you're ready to submit your revision, log on to https://www.editorialmanager.com/pone/ and select the 'Submissions Needing Revision' folder to locate your manuscript file.

- A rebuttal letter that responds to each point raised by the academic editor and reviewer(s). You should upload this letter as a separate file labeled 'Response to Reviewers'.
- A marked-up copy of your manuscript that highlights changes made to the original version. You should upload this as a separate file labeled 'Revised Manuscript with Track Changes'.
- An unmarked version of your revised paper without tracked changes. You should upload this as a separate file labeled 'Manuscript'.

If you would like to make changes to your financial disclosure, please include your updated statement in your cover letter. Guidelines for resubmitting your figure files are available below the reviewer comments at the end of this letter.

If applicable, we recommend that you deposit your laboratory protocols in protocols.io to enhance the reproducibility of your results. Protocols.io assigns your protocol its own identifier (DOI) so that it can be cited independently in the future. For instructions see: https://journals.plos.org/plosone/s/submission-guidelines#loc-laboratory-protocols. Additionally, PLOS ONE offers an option for publishing peer-reviewed Lab Protocol articles, which describe protocols hosted on protocols.io. Read more information on sharing protocols at https://plos.org/protocols?utm_medium=editorial-email&utm_source=authorletters&utm_campaign=protocols.

We look forward to receiving your revised manuscript.

Kind regards,

Hesham M.H. Zakaly, Ph.D.

Academic Editor

PLOS ONE

Journal Requirements:

When submitting your revision, we need you to address these additional requirements.

1. Please ensure that your manuscript meets PLOS ONE's style requirements, including those for file naming. The PLOS ONE style templates can be found at

https://journals.plos.org/plosone/s/file?id=wjVg/PLOSOne_formatting_sample_main_body.pdf and

https://journals.plos.org/plosone/s/file?id=ba62/PLOSOne_formatting_sample_title_authors_affiliations.pdf

2.Thank you for stating the following financial disclosure: [This research was funded by Vietnam National University Ho Chi Minh City (VNU-HCM) under grant number B2025-34-03.].

Please state what role the funders took in the study.  If the funders had no role, please state: ""The funders had no role in study design, data collection and analysis, decision to publish, or preparation of the manuscript.""

If this statement is not correct you must amend it as needed.

Please include this amended Role of Funder statement in your cover letter; we will change the online submission form on your behalf.

3. Thank you for stating the following in the Acknowledgments Section of your manuscript: [This research was funded by Vietnam National University Ho Chi Minh City (VNU-HCM) under grant number B2025-34-03.]

We note that you have provided funding information that is not currently declared in your Funding Statement. However, funding information should not appear in the Acknowledgments section or other areas of your manuscript. We will only publish funding information present in the Funding Statement section of the online submission form.

Please remove any funding-related text from the manuscript and let us know how you would like to update your Funding Statement. Currently, your Funding Statement reads as follows: [This research was funded by Vietnam National University Ho Chi Minh City (VNU-HCM) under grant number B2025-34-03.].

Please include your amended statements within your cover letter; we will change the online submission form on your behalf.

4. We note that the grant information you provided in the ‘Funding Information’ and ‘Financial Disclosure’ sections do not match.

When you resubmit, please ensure that you provide the correct grant numbers for the awards you received for your study in the ‘Funding Information’ section.

5. When completing the data availability statement of the submission form, you indicated that you will make your data available on acceptance. We strongly recommend all authors decide on a data sharing plan before acceptance, as the process can be lengthy and hold up publication timelines. Please note that, though access restrictions are acceptable now, your entire data will need to be made freely accessible if your manuscript is accepted for publication. This policy applies to all data except where public deposition would breach compliance with the protocol approved by your research ethics board. If you are unable to adhere to our open data policy, please kindly revise your statement to explain your reasoning and we will seek the editor's input on an exemption. Please be assured that, once you have provided your new statement, the assessment of your exemption will not hold up the peer review process.

6. If the reviewer comments include a recommendation to cite specific previously published works, please review and evaluate these publications to determine whether they are relevant and should be cited. There is no requirement to cite these works unless the editor has indicated otherwise.

[Note: HTML markup is below. Please do not edit.]

Reviewers' comments:

Reviewer's Responses to Questions

**Comments to the Author**

1. Is the manuscript technically sound, and do the data support the conclusions?

Reviewer #1: Partly

Reviewer #2: Yes

Reviewer #3: Yes

Reviewer #4: Yes

**********

2. Has the statistical analysis been performed appropriately and rigorously?

Reviewer #1: Yes

Reviewer #2: Yes

Reviewer #3: Yes

Reviewer #4: Yes

**********

3. Have the authors made all data underlying the findings in their manuscript fully available?

The PLOS Data policy

Reviewer #1: No

Reviewer #2: Yes

Reviewer #3: Yes

Reviewer #4: Yes

**********

4. Is the manuscript presented in an intelligible fashion and written in standard English?

Reviewer #1: Yes

Reviewer #2: No

Reviewer #3: Yes

Reviewer #4: Yes

**********

Reviewer #1: Despite the statistics and econometric model applied to process data and produce result, the variables that were associated to have effect on one another seems unrelated. For instance, renewable energy is included in the natural resources. But both appeared to be two different variables. The relationship between the selected variables such as renewable energy, information and communication technologies, financial development and sustainable agriculture all seems to have no logical relationship. For example, the authors can see the effect of ICT on Agriculture production; or the effect of financial capacity of farmers on Agriculture productivity.....or the summative effect of "the three" variables on Agriculture Value Addition (if this is considered as dependent variable).

But now, the variables related seems to have spurious correlation unless split and stand alone by themselves to be regressed over "one selected dependent variable"

The title states "the relationship between renewable energy, information and communication technologies, financial development and sustainable agriculture" ---- Sustainable Agriculture

The Abstract says, "each variable has positive/ negative effect on Agriculture Value Addition" not sustainable agriculture like in the title.

The analysis (statistics) processed data to link each variable. But finally arrived on the conclusion that each variable has relationship on Agriculture. This is a vague conclusion

Here, the dependent variable is not clearly stated. Is it;

- Sustainable agriculture (as on the title) or

- Agriculture Value Addition (as in the abstract and in the result) or

- Agriculture (as in the conclusion)

In the theoretical framework, agriculture input and out put (in terms of production) was considered.

Whichever the dependent variable is, the three are different concepts that can't be synonymized. The question for the authors is, have they measured the dependent variable in terms of output (if agriculture production) or Value (if AVA) or made trend analysis (if Sustainable agriculture production was considered)

Generally, the variable relationships are not logically justified. The Literature review part doesn't support the logical relationship of the four variables and the theoretical framework was less related to the empirical studies in the review part.

Recommendation: If the authors have data with them, it is recommended to clearly define the variables, empirically justify the relationship between the variables, and reframe theoretical links between variable and re run the analysis.

Reviewer #2: Please apply the comments provided in the file and provide valid references for the formulas you have used in the text of the article.

Some comments are:

Edit:

Added value…

Use numerical information to express results.

Edit:

The climate….

Edit according to the in-text referencing format.

Replace with:

To make agriculture more sustainable, it is essential to ensure food security, enhance the value of agricultural products, and decrease carbon emissions from the agricultural sector.

Edit:

Added value…

Use numerical information to express results.

Edit:

The climate….

Edit according to the in-text referencing format.

Please provide a valid reference for this section.

Replace with:

In Asia, agriculture plays a vital role in the livelihoods of its people. Over 60% of the population, approximately 2.2 billion individuals, rely on agriculture for their income. However, the agricultural sector's contribution to the region's gross domestic product (GDP) has been declining, falling from an average of 11.1% in 2000 to 7.1% in 2023.

And edit the refrence formatting.

Reviewer #3: The manuscript examines the impact of renewable energy consumption, information and communication technologies, financial development, natural resources, and institutional quality on agricultural value added in 29 Asian countries between 2000 and 2021. Using advanced econometric models (FMOLS, DOLS, DSK, and GMM), the study finds that renewable energy, ICT, financial development, and institutional quality support sustainable agriculture, while natural resources exert a negative “resource curse” effect. The paper highlights policy recommendations to promote clean energy adoption, ICT integration, and financial incentives for green agricultural practices in Asia. Please check forsuggestions and recommendationsin the attachment.

Reviewer #4: Comments to the Author

For the manuscript PONE-D-25-41598 entitled ‘’ The relationship between renewable energy, information and communication technologies, financial development and sustainable agriculture: Evidence from Asia nations which has been submitted to PLOS ONE journal .

Thank you for submitting this manuscript to the PLOS ONE journal. Overall, the work is informative, well-organized, and clearly structured, though some sections require minor improvements.

Comment on the use of abbreviations: once you introduce an abbreviation, please use only the abbreviation thereafter. There is no need to repeat the full term with the abbreviation in parentheses each time. This issue appears throughout your manuscript, so please correct it.

2.2 Natural resources and agriculture sector

under this subtitle there is a sentence it says ‘’ Zhou et al. (2024) discovered a negative nexus between NR abundance and agricultural productivity (AP) in Asia-Pacific Economic Cooperation (APEC) countries, implying that natural resource decreases AP’’. How this could be true?

Methodology section

The Methodology section is written in the future tense, but since the work has already been completed, it should be presented in the past tense. Therefore, this section requires revision.

**********

what does this mean?). If published, this will include your full peer review and any attached files.

If you choose “no”, your identity will remain anonymous but your review may still be made public.

**Do you want your identity to be public for this peer review?**  For information about this choice, including consent withdrawal, please see our Privacy Policy

Reviewer #1: **Yes:**  Mathewos Belissa (Asst. Professor)

Reviewer #2: **Yes:**  Faezeh Behzadipour

Reviewer #3: No

Reviewer #4: No

**********

[NOTE: If reviewer comments were submitted as an attachment file, they will be attached to this email and accessible via the submission site. Please log into your account, locate the manuscript record, and check for the action link "View Attachments". If this link does not appear, there are no attachment files.]

While revising your submission, please upload your figure files to the Preflight Analysis and Conversion Engine (PACE) digital diagnostic tool, https://pacev2.apexcovantage.com/. PACE helps ensure that figures meet PLOS requirements. To use PACE, you must first register as a user. Registration is free. Then, login and navigate to the UPLOAD tab, where you will find detailed instructions on how to use the tool. If you encounter any issues or have any questions when using PACE, please email PLOS at figures@plos.org. Please note that Supporting Information files do not need this step.

Attachment Submitted filename: Manuscrip with author details.docx

Attachment Submitted filename: PONE-D-25-41598.docx

Attachment Submitted filename: Comments to the Author Sep.docx

# Author comment: Author response to Decision Letter 1

**DOI:** 10.1371/journal.pone.0355560.r002

26 Oct 2025

Dear Professor

The authors have responded fully to the Reviewers' comments, and the Response to Reviewers file is attached.

Best regards

Attachment Submitted filename: Response to Reviewers.docx

# Peer review: Decision Letter 1

**DOI:** 10.1371/journal.pone.0355560.r003

**Authors:** Hesham M.H. Zakaly

22 Dec 2025

Dear Dr. Thi,

Please submit your revised manuscript by Feb 05 2026 11:59PM. If you will need more time than this to complete your revisions, please reply to this message or contact the journal office at plosone@plos.org. When you're ready to submit your revision, log on to https://www.editorialmanager.com/pone/ and select the 'Submissions Needing Revision' folder to locate your manuscript file.

- A letter that responds to each point raised by the academic editor and reviewer(s). You should upload this letter as a separate file labeled 'Response to Reviewers'.
- A marked-up copy of your manuscript that highlights changes made to the original version. You should upload this as a separate file labeled 'Revised Manuscript with Track Changes'.
- An unmarked version of your revised paper without tracked changes. You should upload this as a separate file labeled 'Manuscript'.

If applicable, we recommend that you deposit your laboratory protocols in protocols.io to enhance the reproducibility of your results. Protocols.io assigns your protocol its own identifier (DOI) so that it can be cited independently in the future. For instructions see: https://journals.plos.org/plosone/s/submission-guidelines#loc-laboratory-protocols. Additionally, PLOS ONE offers an option for publishing peer-reviewed Lab Protocol articles, which describe protocols hosted on protocols.io. Read more information on sharing protocols at https://plos.org/protocols?utm_medium=editorial-email&utm_source=authorletters&utm_campaign=protocols.

We look forward to receiving your revised manuscript.

Kind regards,

Hesham M.H. Zakaly, Ph.D.

Academic Editor

PLOS One

Journal Requirements:

If the reviewer comments include a recommendation to cite specific previously published works, please review and evaluate these publications to determine whether they are relevant and should be cited. There is no requirement to cite these works unless the editor has indicated otherwise.

Please review your reference list to ensure that it is complete and correct. If you have cited papers that have been retracted, please include the rationale for doing so in the manuscript text, or remove these references and replace them with relevant current references. Any changes to the reference list should be mentioned in the rebuttal letter that accompanies your revised manuscript. If you need to cite a retracted article, indicate the article’s retracted status in the References list and also include a citation and full reference for the retraction notice.

[Note: HTML markup is below. Please do not edit.]

Reviewers' comments:

Reviewer's Responses to Questions

**Comments to the Author**

Reviewer #1: (No Response)

Reviewer #3: All comments have been addressed

Reviewer #4: (No Response)

**********

2. Is the manuscript technically sound, and do the data support the conclusions?

Reviewer #1: Yes

Reviewer #3: Yes

Reviewer #4: Yes

**********

3. Has the statistical analysis been performed appropriately and rigorously?

Reviewer #1: Yes

Reviewer #3: Yes

Reviewer #4: Yes

**********

4. Have the authors made all data underlying the findings in their manuscript fully available?

The PLOS Data policy

Reviewer #1: (No Response)

Reviewer #3: Yes

Reviewer #4: Yes

**********

5. Is the manuscript presented in an intelligible fashion and written in standard English?

Reviewer #1: No

Reviewer #3: Yes

Reviewer #4: Yes

**********

Reviewer #1: I am not satisfied with relating the variables and how each variable affects agriculture productivity. For instance the ICTs support can positively influence agriculture productivity through Accessing market information, Weather forecasts, digital agricultural extension advice, Early warning systems, Digital financial services. Similarly if we take Financial development as a factor to influence productivity, that is through improved access to credit, provision of affordable loans, use of digital banking, insurance and savings ....etc.

In the manuscript, the variables were not well defined to indicate correlation between the dependent and independent.

The points narrated as revised version over the earlier review comment is totally not needed. To describe renewable energy, the authors discussed about burning fossil fuels, carbon dioxide (CO2) releases greenhouse effect and rising global temperature; which are all less related to REC. What the authors should have explained was how renewable energy consumption contribute (directly or indirectly) to Agriculture productivity.

Anyways, the structure of the manuscript seems fine but its contribution to the body of knowledge remains limited.

Reviewer #3: The paper examines how renewable energy, ICT, natural resources, financial development, and institutional quality influence agricultural productivity in 29 Asian countries from 2000–2021. Please check for suggestions and recommendations in the attachment.

Reviewer #4: My comments have been taken care of; however, the conclusion section is still too long. It reads more like a summary than a conclusion.

**********

what does this mean?). If published, this will include your full peer review and any attached files.

If you choose “no”, your identity will remain anonymous but your review may still be made public.

**Do you want your identity to be public for this peer review?**  For information about this choice, including consent withdrawal, please see our Privacy Policy

Reviewer #1: **Yes:**  Mathewos Belissa

Reviewer #3: No

Reviewer #4: No

**********

[NOTE: If reviewer comments were submitted as an attachment file, they will be attached to this email and accessible via the submission site. Please log into your account, locate the manuscript record, and check for the action link "View Attachments". If this link does not appear, there are no attachment files.]

To ensure your figures meet our technical requirements, please review our figure guidelines: https://journals.plos.org/plosone/s/figures

You may also use PLOS’s free figure tool, NAAS, to help you prepare publication quality figures: https://journals.plos.org/plosone/s/figures#loc-tools-for-figure-preparation.

NAAS will assess whether your figures meet our technical requirements by comparing each figure against our figure specifications.

Attachment Submitted filename: PONE-D-25-41598_R1.docx

# Author comment: Author response to Decision Letter 2

**DOI:** 10.1371/journal.pone.0355560.r004

24 Jan 2026

Dear Professor,

The authors have responded to all comments from the Reviewers and Editor. We have attached the "Response to Reviewers" file and have revised the manuscript according to the suggestions of all Reviewers.

Sincerely,

Attachment Submitted filename: Response_to_Reviewers_auresp_2.docx

# Peer review: Decision Letter 2

**DOI:** 10.1371/journal.pone.0355560.r005

**Authors:** Hesham M.H. Zakaly

2 Feb 2026

Dear Dr. Thi,

Thank you for submitting your manuscript to PLOS ONE. After careful consideration, we feel that it has merit but does not fully meet PLOS ONE’s publication criteria as it currently stands. Therefore, we invite you to submit a revised version of the manuscript that addresses the points raised during the review process.

plosone@plos.org. When you're ready to submit your revision, log on to https://www.editorialmanager.com/pone/ and select the 'Submissions Needing Revision' folder to locate your manuscript file.

- A letter that responds to each point raised by the academic editor and reviewer(s). You should upload this letter as a separate file labeled 'Response to Reviewers'.
- A marked-up copy of your manuscript that highlights changes made to the original version. You should upload this as a separate file labeled 'Revised Manuscript with Track Changes'.
- An unmarked version of your revised paper without tracked changes. You should upload this as a separate file labeled 'Manuscript'.

If applicable, we recommend that you deposit your laboratory protocols in protocols.io to enhance the reproducibility of your results. Protocols.io assigns your protocol its own identifier (DOI) so that it can be cited independently in the future. For instructions see: https://journals.plos.org/plosone/s/submission-guidelines#loc-laboratory-protocols. Additionally, PLOS ONE offers an option for publishing peer-reviewed Lab Protocol articles, which describe protocols hosted on protocols.io. Read more information on sharing protocols at https://plos.org/protocols?utm_medium=editorial-email&utm_source=authorletters&utm_campaign=protocols.

We look forward to receiving your revised manuscript.

Kind regards,

Hesham M.H. Zakaly, Ph.D.

Academic Editor

PLOS One

Journal Requirements:

1. If the reviewer comments include a recommendation to cite specific previously published works, please review and evaluate these publications to determine whether they are relevant and should be cited. There is no requirement to cite these works unless the editor has indicated otherwise.

2. Please review your reference list to ensure that it is complete and correct. If you have cited papers that have been retracted, please include the rationale for doing so in the manuscript text, or remove these references and replace them with relevant current references. Any changes to the reference list should be mentioned in the rebuttal letter that accompanies your revised manuscript. If you need to cite a retracted article, indicate the article’s retracted status in the References list and also include a citation and full reference for the retraction notice.

[Note: HTML markup is below. Please do not edit.]

Reviewers' comments:

Reviewer's Responses to Questions

**Comments to the Author**

Reviewer #1: (No Response)

**********

2. Is the manuscript technically sound, and do the data support the conclusions?

Reviewer #1: Partly

**********

3. Has the statistical analysis been performed appropriately and rigorously?

Reviewer #1: N/A

**********

4. Have the authors made all data underlying the findings in their manuscript fully available?

The PLOS Data policy

Reviewer #1: No

**********

5. Is the manuscript presented in an intelligible fashion and written in standard English?

Reviewer #1: Yes

**********

Reviewer #1: The author used sophisticated statistics, but my concern has not been addressed through out. No need to repeat, but to remind, the title of the manuscript is about "The relationship between renewable energy, information and communication technologies, financial development and agricultural productivity".

REVIEW SESSION EXPLAINS about

1. Renewable energy

2. Natural resource

3. Information and communication technologies, as agricultural productivity determining factors:

From Research Gap (2.5) one can read as if the title of the research is;

1. The role of ICT in using Mobile phone for information exchange

2. It also looks as “role of ICT in the effective deployment and management of renewable energy”

FINDING SESSION talks about the effect ….

The effect of renewable energy, information and communication technologies, natural resources, financial development and institutional quality on AGP.

Generally, I couldn't see any relationship between the title and the main part of the paper including statistical results. My final recommendation is to align the title-review-objectives, results-findings-recommendation.

**********

what does this mean?). If published, this will include your full peer review and any attached files.

If you choose “no”, your identity will remain anonymous but your review may still be made public.

**Do you want your identity to be public for this peer review?**  For information about this choice, including consent withdrawal, please see our Privacy Policy

Reviewer #1: No

**********

[NOTE: If reviewer comments were submitted as an attachment file, they will be attached to this email and accessible via the submission site. Please log into your account, locate the manuscript record, and check for the action link "View Attachments". If this link does not appear, there are no attachment files.]

To ensure your figures meet our technical requirements, please review our figure guidelines: https://journals.plos.org/plosone/s/figures

You may also use PLOS’s free figure tool, NAAS, to help you prepare publication quality figures: https://journals.plos.org/plosone/s/figures#loc-tools-for-figure-preparation.

NAAS will assess whether your figures meet our technical requirements by comparing each figure against our figure specifications.

# Author comment: Author response to Decision Letter 3

**DOI:** 10.1371/journal.pone.0355560.r006

13 Mar 2026

Dear Professor,

We have revised the Manuscript according to your suggestions.

Sincerely,

Attachment Submitted filename: Response_to_Reviewers_auresp_3.docx

# Editor report: Decision Letter 3

**DOI:** 10.1371/journal.pone.0355560.r007

**Authors:** Hesham M.H. Zakaly

23 Jul 2026

Renewable energy, information and communication technology, financial development, natural resources, institutional quality, and agricultural productivity in Asian countries

PONE-D-25-41598R3

Dear Dr. Thi,

We’re pleased to inform you that your manuscript has been judged scientifically suitable for publication and will be formally accepted for publication once it meets all outstanding technical requirements.

Within one week, you’ll receive an e-mail detailing the required amendments. When these have been addressed, you’ll receive a formal acceptance letter and your manuscript will be scheduled for publication.

An invoice will be generated when your article is formally accepted. Please note, if your institution has a publishing partnership with PLOS and your article meets the relevant criteria, all or part of your publication costs will be covered. Please make sure your user information is up-to-date by logging into Editorial Manager at Editorial Manager® and clicking the ‘Update My Information' link at the top of the page. For questions related to billing, please contact billing support.

If your institution or institutions have a press office, please notify them about your upcoming paper to help maximize its impact. If they’ll be preparing press materials, please inform our press team as soon as possible -- no later than 48 hours after receiving the formal acceptance. Your manuscript will remain under strict press embargo until 2 pm Eastern Time on the date of publication. For more information, please contact onepress@plos.org.

Kind regards,

Hesham M.H. Zakaly, Ph.D.

Academic Editor

PLOS One

Additional Editor Comments (optional):

Reviewers' comments:

# Editor report: Acceptance letter

**DOI:** 10.1371/journal.pone.0355560.r008

**Authors:** Hesham M.H. Zakaly

PONE-D-25-41598R3

PLOS One

Dear Dr. Thi,

I'm pleased to inform you that your manuscript has been deemed suitable for publication in PLOS One. Congratulations! Your manuscript is now being handed over to our production team.

At this stage, our production department will prepare your paper for publication. This includes ensuring the following:

* All references, tables, and figures are properly cited

* All relevant supporting information is included in the manuscript submission,

* There are no issues that prevent the paper from being properly typeset

You will receive further instructions from the production team, including instructions on how to review your proof when it is ready. Please keep in mind that we are working through a large volume of accepted articles, so please give us a few days to review your paper and let you know the next and final steps.

Lastly, if your institution or institutions have a press office, please let them know about your upcoming paper now to help maximize its impact. If they'll be preparing press materials, please inform our press team within the next 48 hours. Your manuscript will remain under strict press embargo until 2 pm Eastern Time on the date of publication. For more information, please contact onepress@plos.org.

You will receive an invoice from PLOS for your publication fee after your manuscript has reached the completed accept phase. If you receive an email requesting payment before acceptance or for any other service, this may be a phishing scheme. Learn how to identify phishing emails and protect your accounts at https://explore.plos.org/phishing.

If we can help with anything else, please email us at customercare@plos.org.

Thank you for submitting your work to PLOS ONE and supporting open access.

Kind regards,

PLOS ONE Editorial Office Staff

on behalf of

Dr. Hesham M.H. Zakaly

Academic Editor

PLOS One
